# Urban form datasets of 194 cities delineated based on the contiguous urban fabric for 1990 and 2015

**DOI:** 10.1016/j.dib.2020.106369

**Published:** 2020-10-06

**Authors:** Richard Lemoine-Rodriguez, Luis Inostroza, Harald Zepp

**Affiliations:** a Institute of Geography, Ruhr University, Universitätsstr. 150, 44780 Bochum, Germany; b Universidad Autónoma de Chile, Avda. Pedro de Valdivia 420, Providencia, Santiago, Chile

**Keywords:** Urban fabric, Urban form, Land cover, Urban expansion, Landscape change

## Abstract

The present research datasets were processed for the article “The global homogenization of urban form. An assessment of 194 cities across time” [1]. They consist of land cover spatial layers, longitude and latitude point data and tabulated data with computed landscape metrics and the characterization of urban form of 194 cities for 1990 and 2015. Contiguous urban fabric at 30 m spatial resolution was derived from the Atlas of Urban Expansion database for 1990 and 2015 [2]. Landscape metrics were computed as quantitative measures of composition and spatial arrangement of each city and dimensions of the database were reduced employing correlation and principal components analysis. Hierarchical clustering was employed to group cities according to the similarity of their urban form and analysis of variance was applied to test for significant differences between them. The spatial layers contained in this article can be complemented with past and future land cover data to model urban form change at broader temporal scales. The landscape metrics are useful for cross-city comparisons at regional, national and global levels in combination with other complementary indicators. The datasets are valuable for urban planners, urban ecologists, NGO's, decision makers and other with interest on local and global landscape change in urban areas, particularly urban expansion and its impacts.

## Specifications Table

**Table:** 

Subject	Environmental science
Specific subject area	Land cover change, urban form, urban sprawl
Type of data	Table Raster Vector
How data were acquired	Primary data corresponds to land cover raster layers of 200 cities contained in the Atlas of Urban Expansion of the Lincoln Institute of Land Policy [2], available at: http://www.atlasofurbanexpansion.org/data Secondary data was derived of raster processing steps on the environment for statistical analysis R
Data format	Raw data (tif, shapefile, csv)
Parameters for data collection	We exclusively employed cities with urban pixels. Therefore, we excluded six cities which were covered only by suburban pixels in 1990 according to the low density of their built-up
Description of data collection	Spatially adjacent urban pixels and embedded suburban areas, open space and water of each city were extracted from the Atlas of Urban Expansion database, taking as starting point the central business district. When two clusters of urban pixels were divided by rivers, both were retained
Data source location	194 cities (world-wide)
Data accessibility	Secondary data are included in this paper
Related research article	R. Lemoine-Rodríguez, L. Inostroza, H. Zepp. The global homogenization of urban form. An assessment of 194 cities across time, Landscape and Urban Planning, 204103949, (2020). https://doi.org/10.1016/j.landurbplan.2020.103949

## Value of the Data

• The data shows how the urban form of the contiguous urban fabric of 194 cities has changed from 1990 to 2015.
• This datasets are of interest for urban planners, urban ecologists, NGO's, decision makers and other with interest on local and global landscape change in urban areas.
• The spatial layers can be complemented with past and future land cover data to model urban form change at broader temporal scales.
• The landscape metrics can be employed for cross-city comparisons at regional, national and global levels in combination with other complementary indicators.
• The spatial layers include small (<10 km^2^) to very large cities (>5000 km^2^), representing the wide variability of cities’ sizes.

## 1 Data Description

**Fig. 1:**
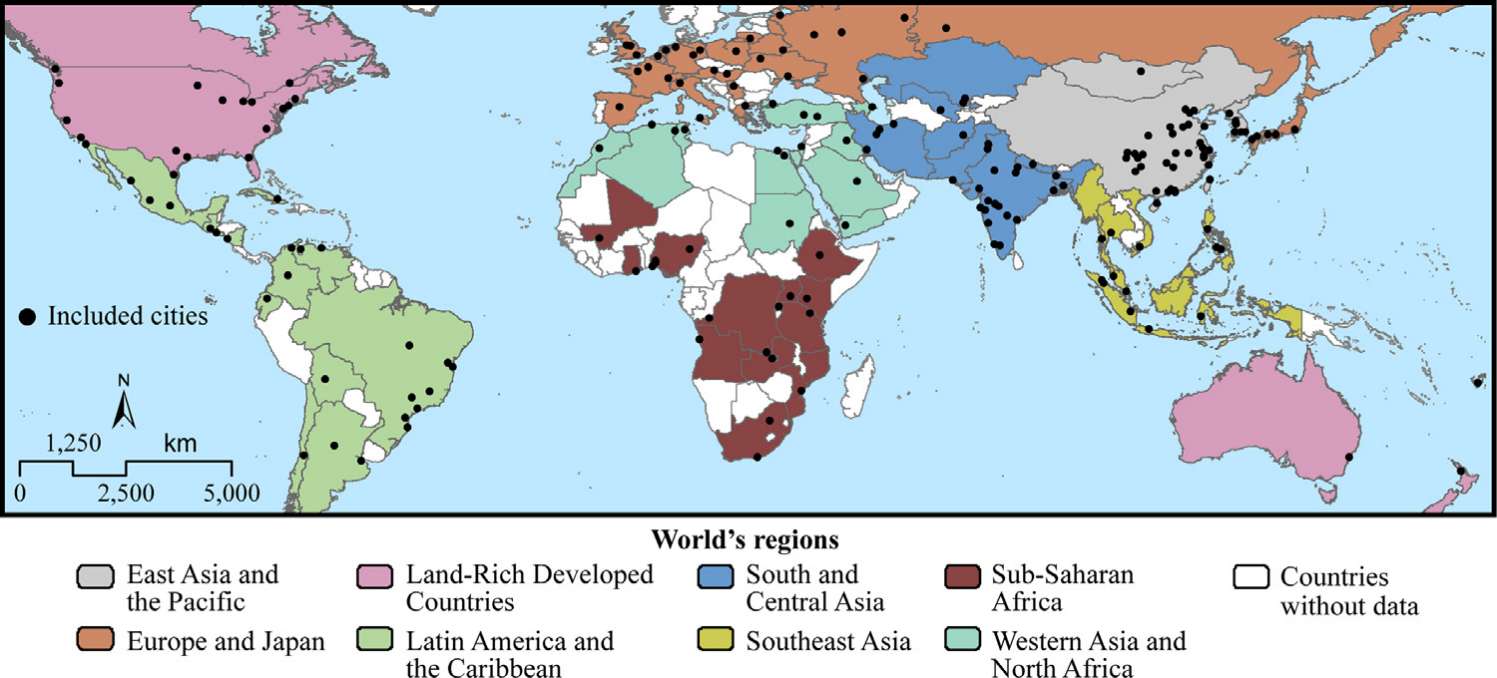
Location of the 194 cities included in the database.

**Fig. 2:**
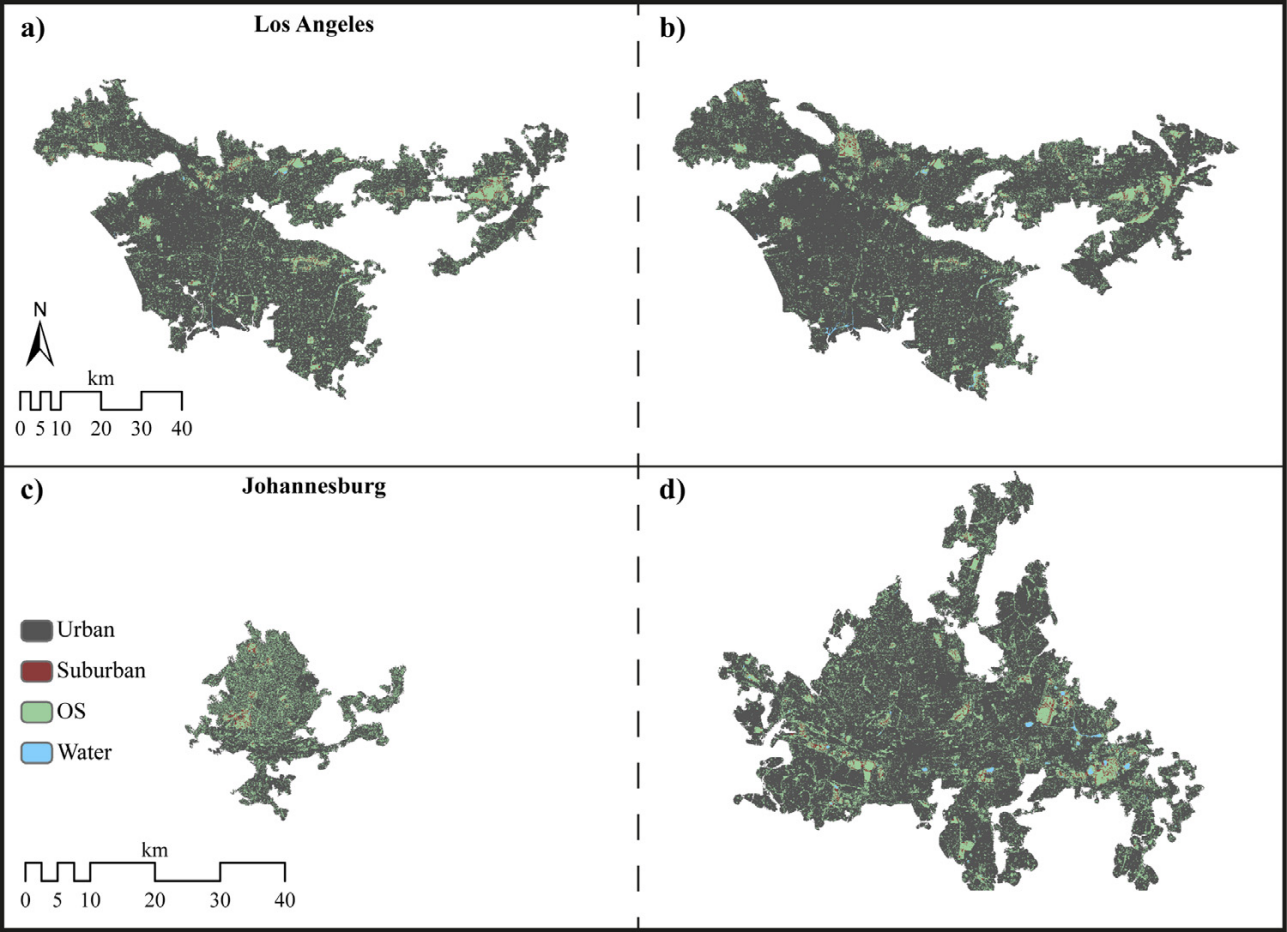
Examples of the extracted contiguous urban fabric. Panels (a) and (c) represent 1990 and (b) and (d) 2015 for Los Angeles (upper) and Johannesburg (lower panel).
• A vector spatial points layer (shapefile) corresponding to the inner centroid of each city is also included as “Cities_centroids” supplementary file (Fig. 1).
• To complement this, tabulated data (.csv table) with the corresponding region and continent, computed landscape metrics, date, and cluster number and description (i.e., type of cluster) for each city is added with the file name “Landscape_metrics” as supplementary data.

The data described in this data paper are of three different types related to land cover spatial layers corresponding to the contiguous urban fabric of 194 cities for 1990 and 2015:

• Raster (.tif) GIS spatial layers of 194 cities selected based on the eight world's regions according to Unites Nations’ classification [3] are included as supplementary “Landcover” files (Fig. 1). Each spatial layer consists of the land cover classes: urban, suburban, open space (OS) and water (Fig. 2). Data is derived from cloud-free Landsat images with a spatial resolution of 30 m for ∼1990 and ∼2015.

## 2 Experimental Design, Materials and Methods

### 2.1 Reference database

**Fig. 3:**
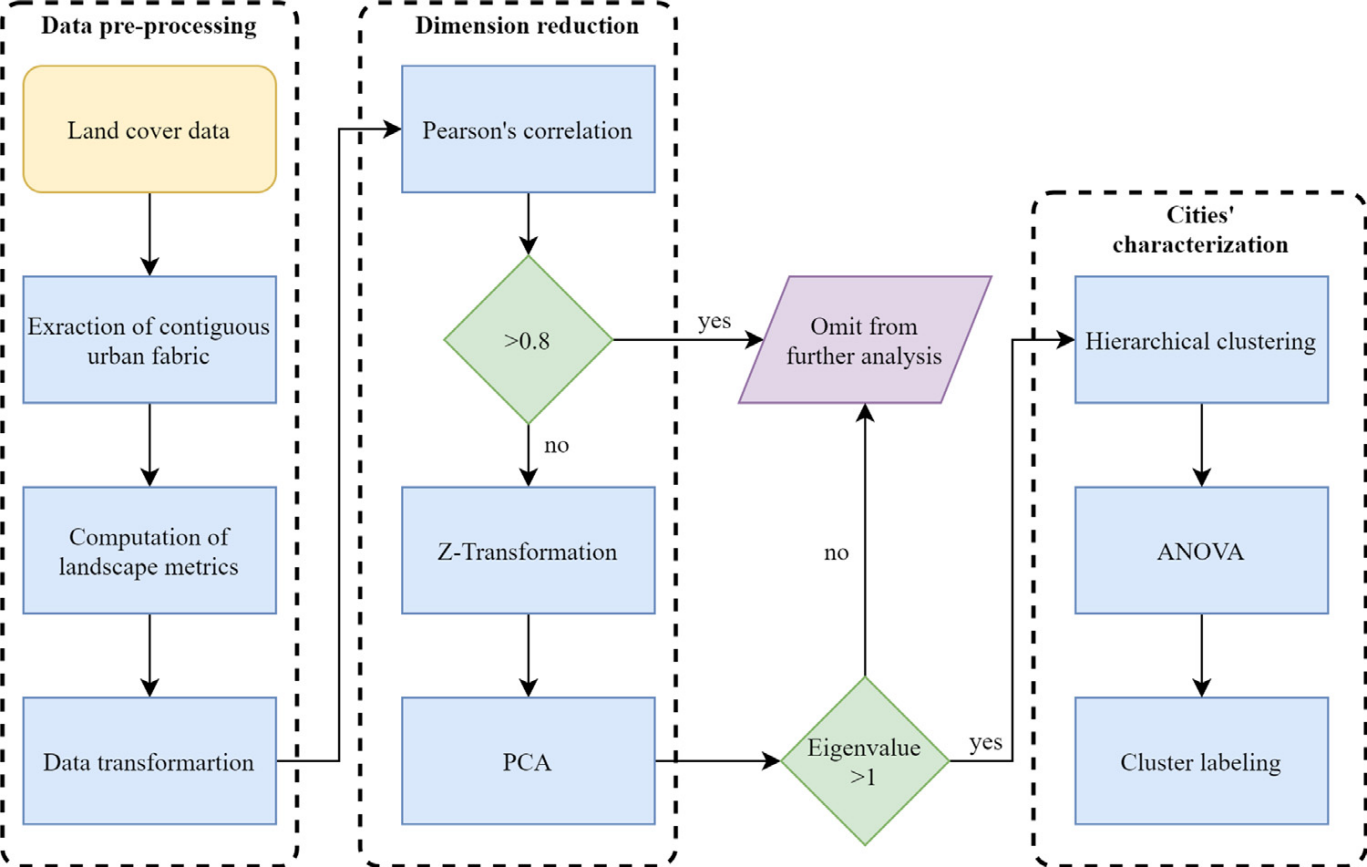
Flow diagram of the conducted steps to generate our datasets. The work flow included image pre and processing, data dimension reduction, cluster analysis and ANOVA.

In order to characterize global urban form for 1990 and 2015, we carried out steps of image pre and processing, data dimension reduction, cluster analysis and ANOVA (Fig. 3). We employed land cover data from the Atlas of Urban Expansion [2]. This database is comprised by a sample of 200 cities, selected following a stratified random sampling, taking into account: (1) eight different geographical regions based on the stratification presented in the World Urbanization Prospects [3] (i.e., East Asia and the Pacific, Southeast Asia, South and Central Asia, Western Asia and North Africa, Sub-Saharan Africa, Latin America and the Caribbean, Europe and Japan and land-rich developed Countries: Canada, USA, Australia and New Zealand), (2) the number of inhabitants in four different ranges (i.e., 100,000–427,000, 427,001–1570,000, 1570,001–5715,000 and 5715,001 or more), and (3) the number of cities in the country (i.e., 1–9, 10–19, 20 or more). Based on these criteria, 56 small cities, 50 medium-sized ones, 54 large ones, and 40 very large ones are part of the database of the Atlas of Urban Expansion according to their population number.

### 2.2 Urban delineation

In order to delineate the contiguous urban fabric for further analysis, we extracted the contiguous urban pixels and embedded suburban and OS and water pixels for each city for 1990 and 2015, employing the central business district as starting point. When two patches were divided by rivers, all polygons adjacent to these features were included in the final city footprint. Hindupur, Rajshahi, Rawang, Singrauli, Vinh Long and Xucheng were excluded from the analysis since they were composed by suburban pixels in 1990, according to the low density of their built-up.

### 2.3 Computing of landscape metrics

We computed 11 landscape metrics at class level that describe different characteristics of urban form. Class Area of urban pixels (CA), class area of Suburban pixels (SU), Ratio of Open Space (ROS), and Area-Weighted Mean Patch Size (AWMPS) were included as measures of composition. Patch Density (PD), Edge Density (ED), and Aggregation Index (AI) were employed as descriptors of fragmentation of the urban tissue. Landscape Shape Index (LSI), Area-Weighted Mean Patch Fractal Dimension (AWMPFD), Area-Weighted Mean Patch Shape Index (AWMSI), and Area-Weighted Mean Perimeter-Area Ratio (AWMPAR) accounted for the cities’ shape complexity. All metrics except ROS and SU were computed for the urban class in the Spatial Pattern Analysis Program for Categorical Maps Fragstats [4] (Table 1).

**Table 1:** List of computed Landscape metrics.

Metric	Description
Class Area of urban pixels (CA)	Spatial extent covered by urban class.
Class area of suburban pixels (SU)	Spatial extent covered by suburban class.
Ratio of Open Space (ROS)	Proportion of the city covered by OS.
Area-Weighted Mean Patch Size (AWMPS)	Area weighted average patch size of patches of the urban class.
Patch Density (PD)	Number of patches per city area.
Edge Density (ED)	Edge of the length relative to the area of the patch.
Aggregation Index (AI)	Measures the number of like adjacencies of corresponding class from 0 (no adjacencies) to 100.
Landscape Shape Index (LSI)	Measure of aggregation or clumpiness.
Area Weighted Mean Patch Fractal Dimension (AWMPFD)	Indicates the form of the urban patch. It equals 1 for circular features and increases with irregularity.
Area Weighted Mean Patch Shape Index (AWMSI)	Indicates the regularity of the patches. Equals 1 for circular features or square cells and increases with irregularity.
Area-Weighted Mean Perimeter-Area Ratio (AWMPAR)	Indicates the complexity of the shape weighted by the area of the patch.

### 2.4 Data transformation

To meet the assumptions of further parametric statistical procedures, we normalize distributions and reduce skewness and kurtosis of the indicators. CA, SU, PD, LSI, AWMPS, AWMSI and AWMPAR were transformed to log base e. The square root of ED was employed, while ROS and AI were box-cox transformed.

### 2.5 Data dimension reduction

We computed a Pearson correlation matrix to avoid collinearity in our data. For combinations with a correlation coefficient >0.80, one of the metrics was excluded from further analysis (Fig. 3). All metrics were centered and scaled with z-transformation to make them comparable. We integrated a single database containing the final set of indicators for 1990 and 2015, keeping their correspondent date label and performed a Principal Components Analysis (PCA) to reduce the dimension of our metrics. We employed the Kaiser's criterion for the selection of Principal Components (PCs), only retaining components with eigenvalues >1 (Fig. 3). We employed varimax orthogonal rotation of eigenvectors (factors), using factor loadings after rotation.

### 2.6 Data clustering

We carried out a hierarchical clustering to group the cities according to their urban form. We computed a distance matrix between values of the retained PCs (z-scores) employing euclidian distance and Ward's agglomeration method. To define the number of clusters different indices of cluster validity were computed (for the list of indices see Table 2 in [5]) employing the NbClust package [5], in the environment for statistical computing R [6]. After computing the results for all indices, the package applied a majority rule of agreement between indices to define the optimal number of clusters. Once our database contained a cluster label for each city by date, we split the data for 1990 and 2015.

### 2.7 Cities’ characterization

To characterize the different types of urban form and test for differences between clusters for each date, we carried out a one-way analysis of variance (ANOVA), using the z-scores of the set of metrics derived after Pearson's correlation test as response variables and the urban form clusters as factors.

## Declaration of Competing Interest

The authors declare that they have no known competing financial interests or personal relationships which have, or could be perceived to have, influenced the work reported in this article.

## Appendix Supplementary materials

Image, application 1
