# Supplementary figures and images for: Urban form datasets of 194 cities delineated based on the contiguous urban fabric for 1990 and 2015 (part 2 of 2)
Source: Data Brief. 2020 Oct 6;33:106369. doi: 10.1016/j.dib.2020.106369 (PMC7569292; doi:10.1016/j.dib.2020.106369)

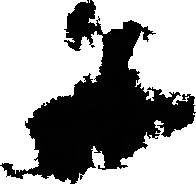

Supplement: Supplementary file 1 [file mmc1.zip › Supplementary/Landcover/Gainesville_FL_1990.tif]

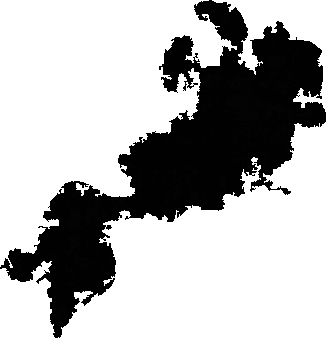

Supplement: Supplementary file 1 [file mmc1.zip › Supplementary/Landcover/Gainesville_FL_2015.tif]

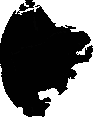

Supplement: Supplementary file 1 [file mmc1.zip › Supplementary/Landcover/Gaoyou_Jiangsu_1990.tif]

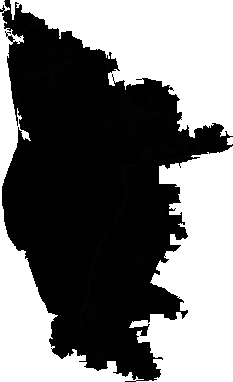

Supplement: Supplementary file 1 [file mmc1.zip › Supplementary/Landcover/Gaoyou_Jiangsu_2015.tif]

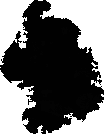

Supplement: Supplementary file 1 [file mmc1.zip › Supplementary/Landcover/Gombe_1990.tif]

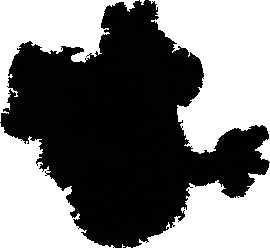

Supplement: Supplementary file 1 [file mmc1.zip › Supplementary/Landcover/Gombe_2015.tif]

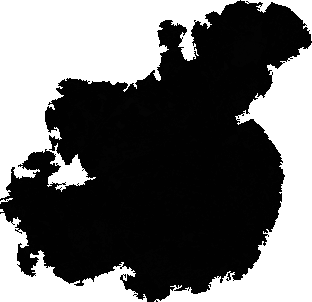

Supplement: Supplementary file 1 [file mmc1.zip › Supplementary/Landcover/Gomel_1990.tif]

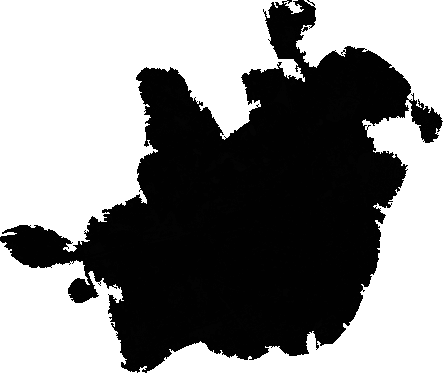

Supplement: Supplementary file 1 [file mmc1.zip › Supplementary/Landcover/Gomel_2015.tif]

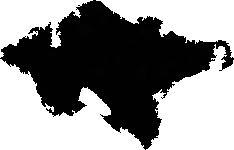

Supplement: Supplementary file 1 [file mmc1.zip › Supplementary/Landcover/Gorgan_1990.tif]

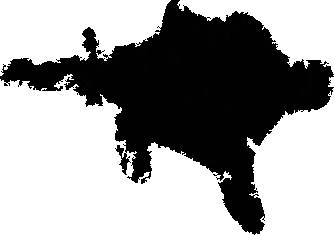

Supplement: Supplementary file 1 [file mmc1.zip › Supplementary/Landcover/Gorgan_2015.tif]

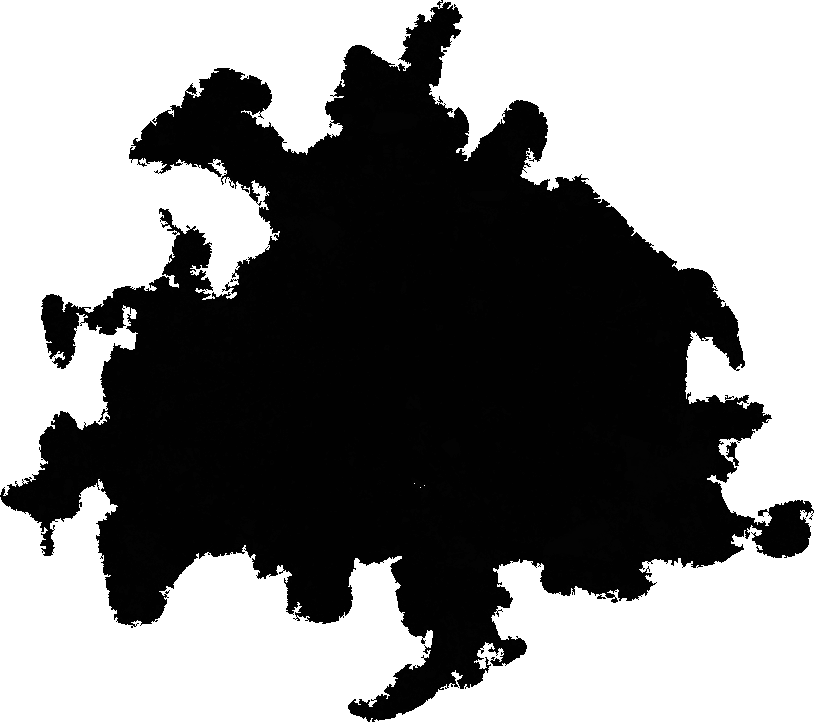

Supplement: Supplementary file 1 [file mmc1.zip › Supplementary/Landcover/Guadalajara_1990.tif]

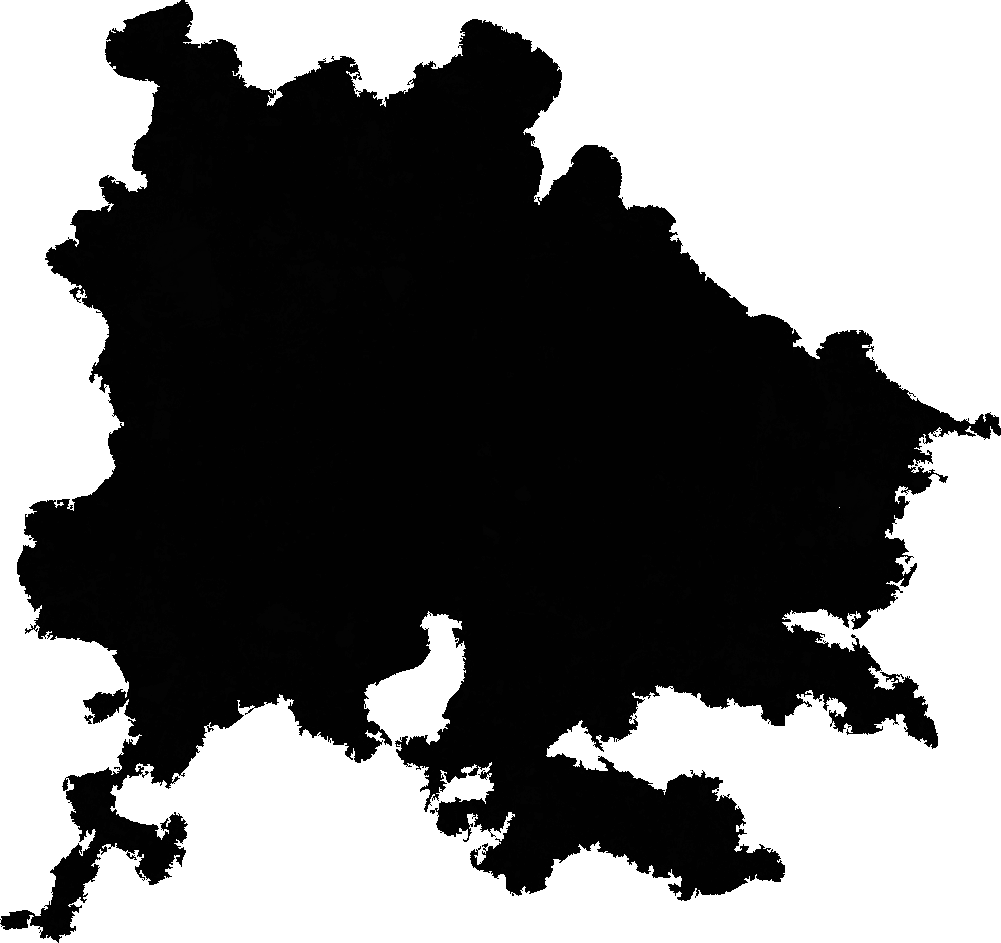

Supplement: Supplementary file 1 [file mmc1.zip › Supplementary/Landcover/Guadalajara_2015.tif]

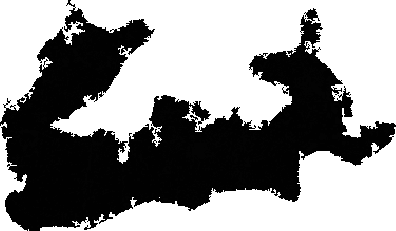

Supplement: Supplementary file 1 [file mmc1.zip › Supplementary/Landcover/Guangzhou_Guangdong_1990.tif]

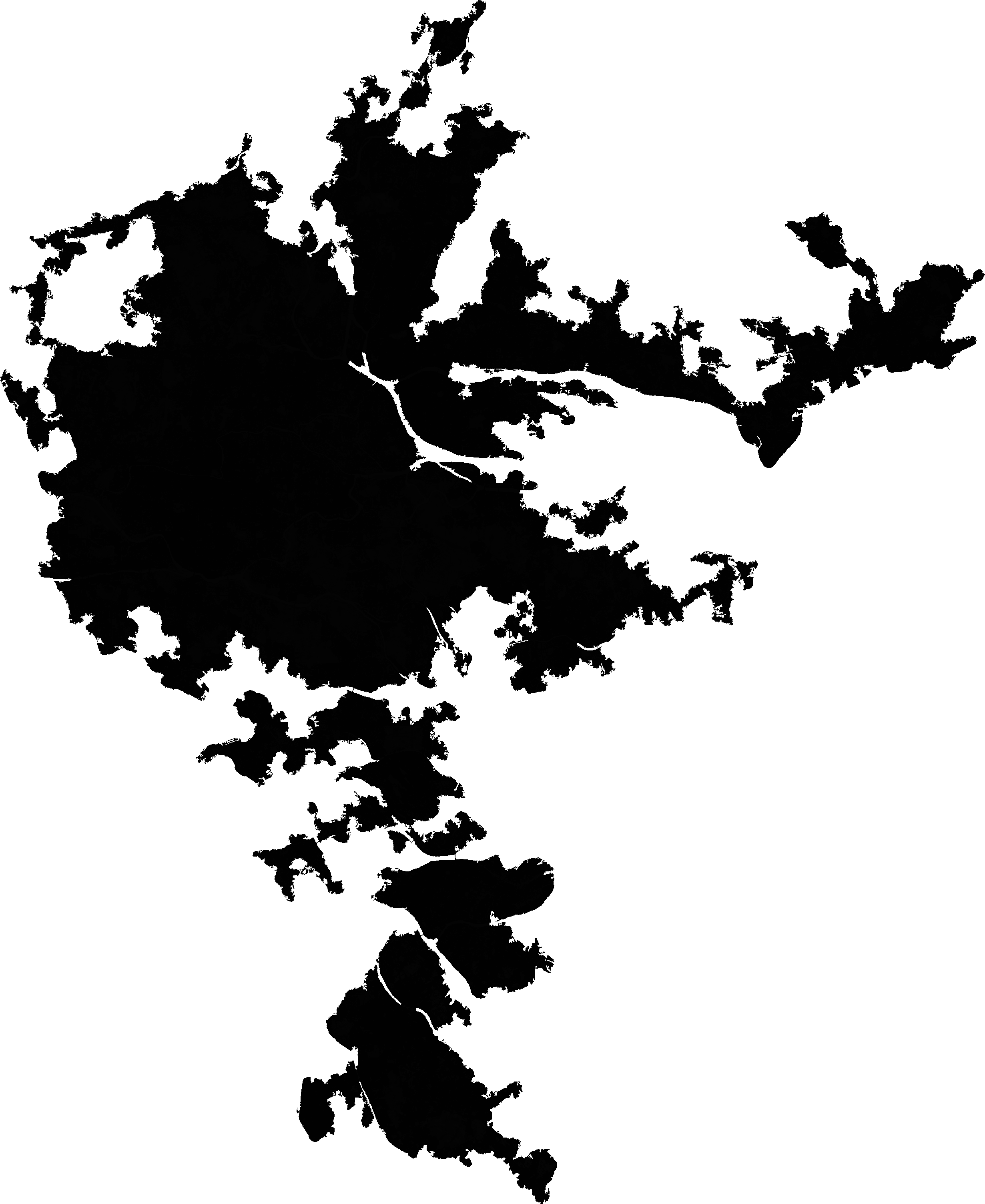

Supplement: Supplementary file 1 [file mmc1.zip › Supplementary/Landcover/Guangzhou_Guangdong_2015.tif]

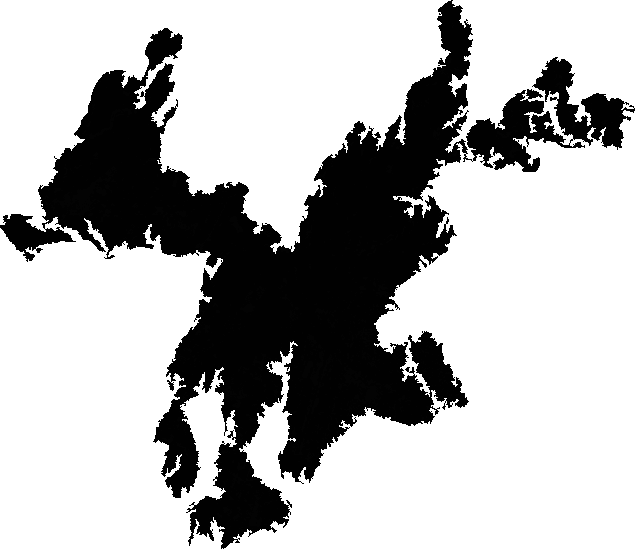

Supplement: Supplementary file 1 [file mmc1.zip › Supplementary/Landcover/Guatemala_City_1990.tif]

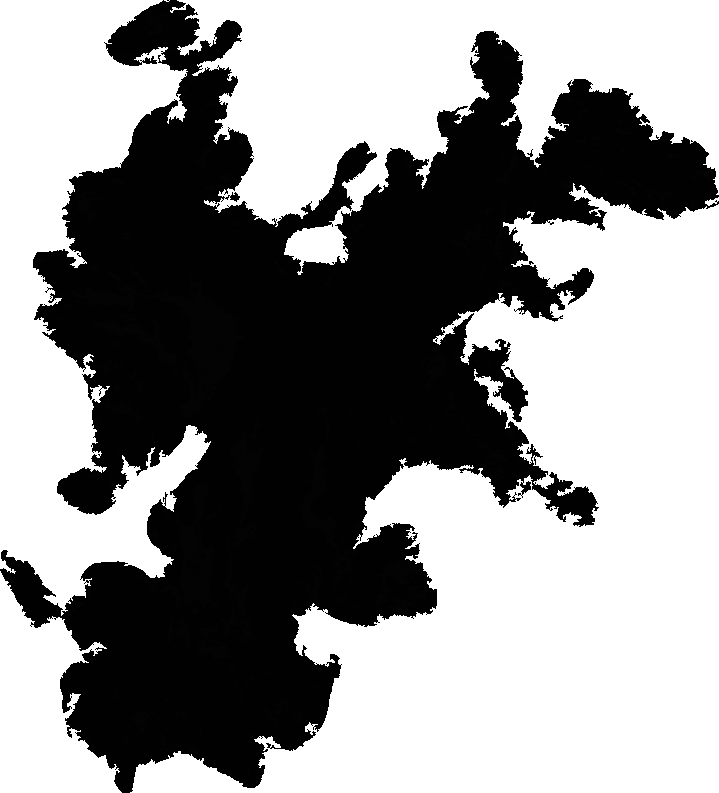

Supplement: Supplementary file 1 [file mmc1.zip › Supplementary/Landcover/Guatemala_City_2015.tif]

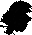

Supplement: Supplementary file 1 [file mmc1.zip › Supplementary/Landcover/Guixi_Chongqing_1990.tif]

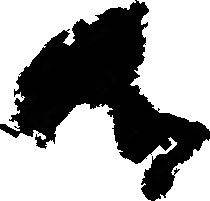

Supplement: Supplementary file 1 [file mmc1.zip › Supplementary/Landcover/Guixi_Chongqing_2015.tif]

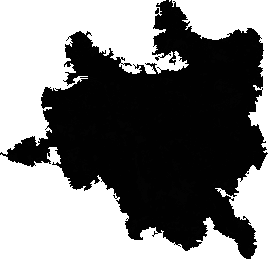

Supplement: Supplementary file 1 [file mmc1.zip › Supplementary/Landcover/Gwangju_1990.tif]

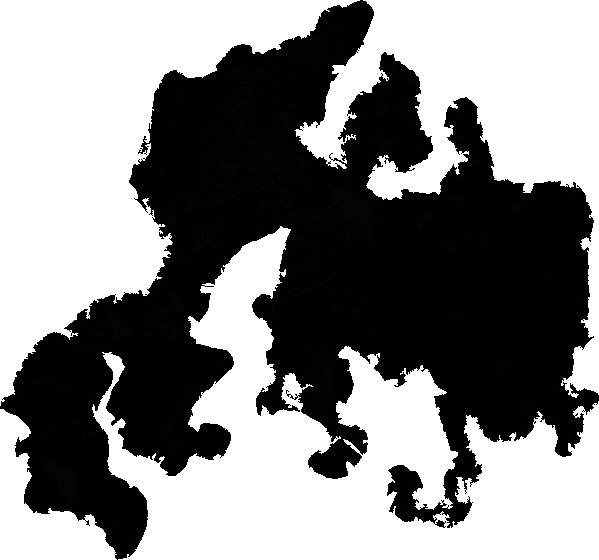

Supplement: Supplementary file 1 [file mmc1.zip › Supplementary/Landcover/Gwangju_2015.tif]

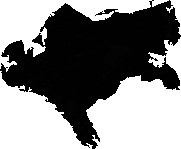

Supplement: Supplementary file 1 [file mmc1.zip › Supplementary/Landcover/Haikou_Hainan_1990.tif]

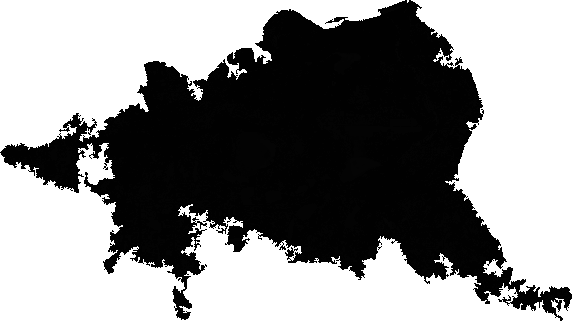

Supplement: Supplementary file 1 [file mmc1.zip › Supplementary/Landcover/Haikou_Hainan_2015.tif]

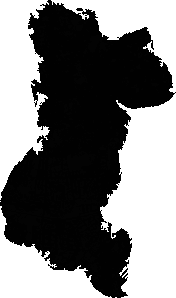

Supplement: Supplementary file 1 [file mmc1.zip › Supplementary/Landcover/Halle_1990.tif]

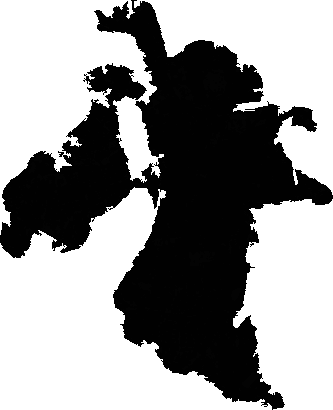

Supplement: Supplementary file 1 [file mmc1.zip › Supplementary/Landcover/Halle_2015.tif]

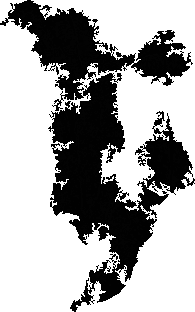

Supplement: Supplementary file 1 [file mmc1.zip › Supplementary/Landcover/Hangzhou_Zhejiang_1990.tif]

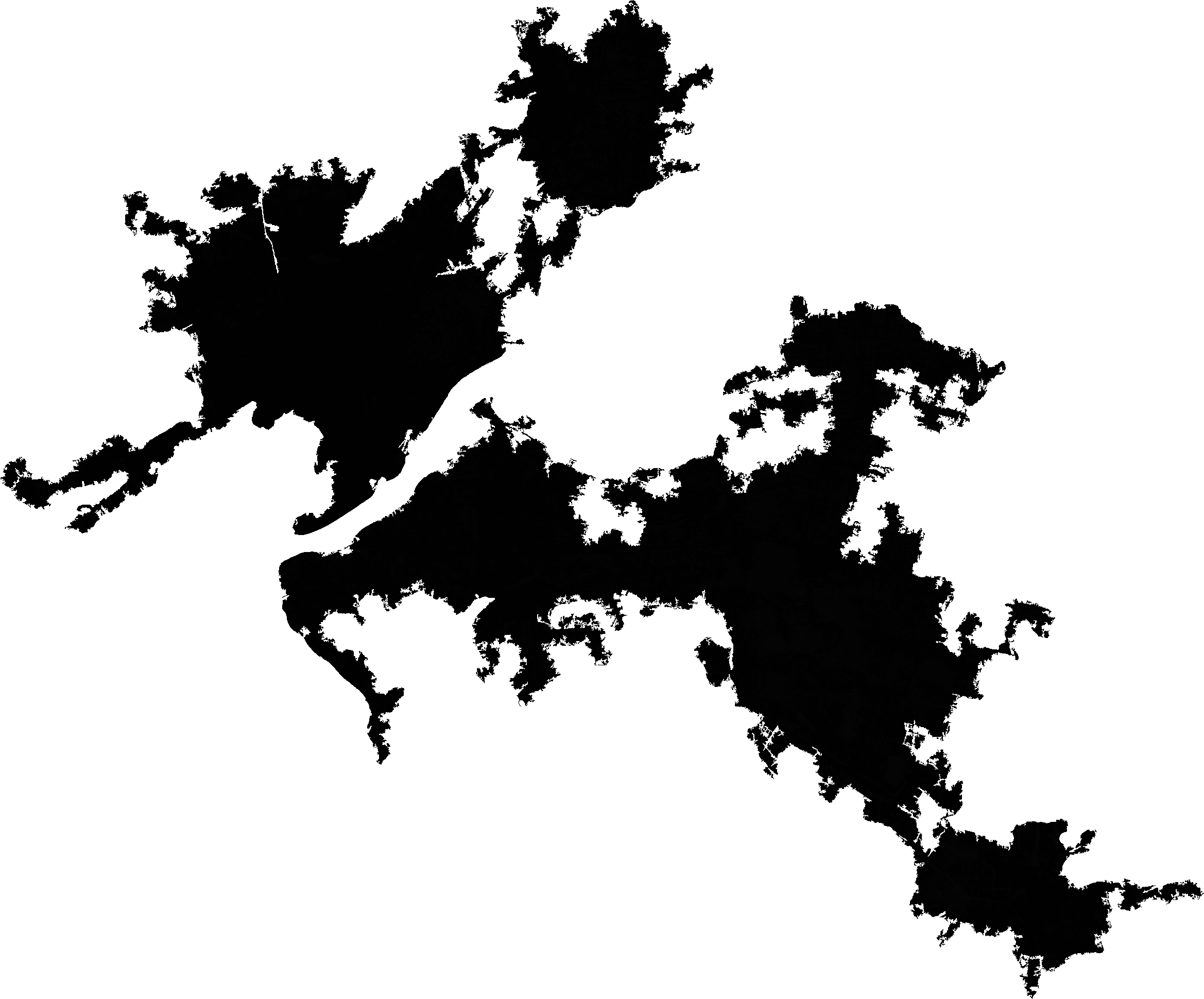

Supplement: Supplementary file 1 [file mmc1.zip › Supplementary/Landcover/Hangzhou_Zhejiang_2015.tif]

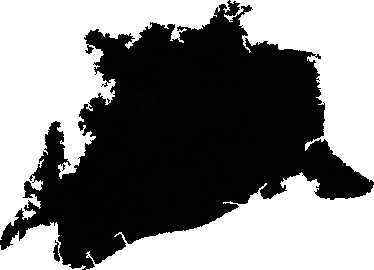

Supplement: Supplementary file 1 [file mmc1.zip › Supplementary/Landcover/Ho_Chi_Minh_City_1990.tif]

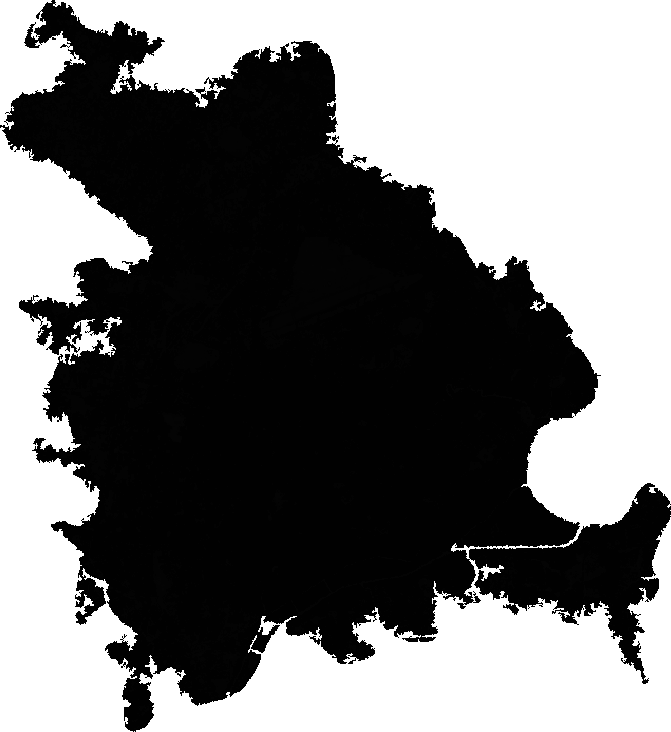

Supplement: Supplementary file 1 [file mmc1.zip › Supplementary/Landcover/Ho_Chi_Minh_City_2015.tif]

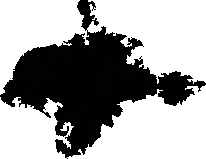

Supplement: Supplementary file 1 [file mmc1.zip › Supplementary/Landcover/Holguin_1990.tif]

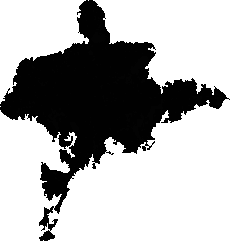

Supplement: Supplementary file 1 [file mmc1.zip › Supplementary/Landcover/Holguin_2015.tif]

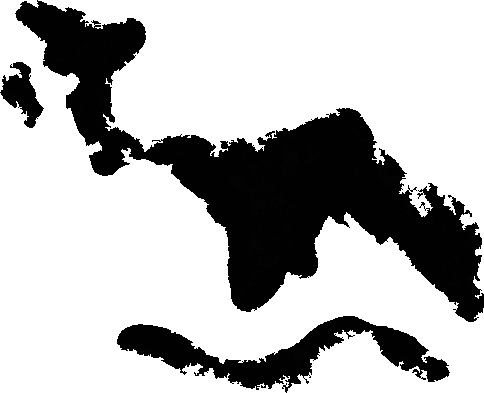

Supplement: Supplementary file 1 [file mmc1.zip › Supplementary/Landcover/Hong_Kong_Hong_Kong_1990.tif]

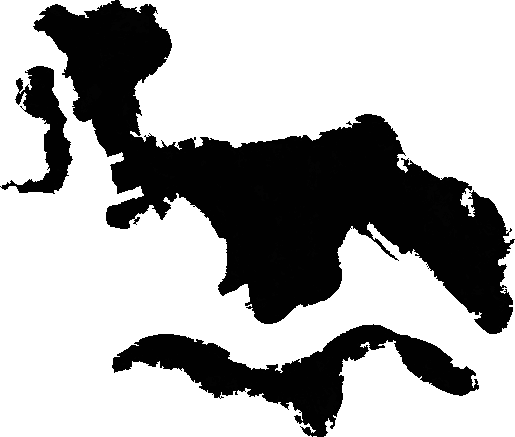

Supplement: Supplementary file 1 [file mmc1.zip › Supplementary/Landcover/Hong_Kong_Hong_Kong_2015.tif]

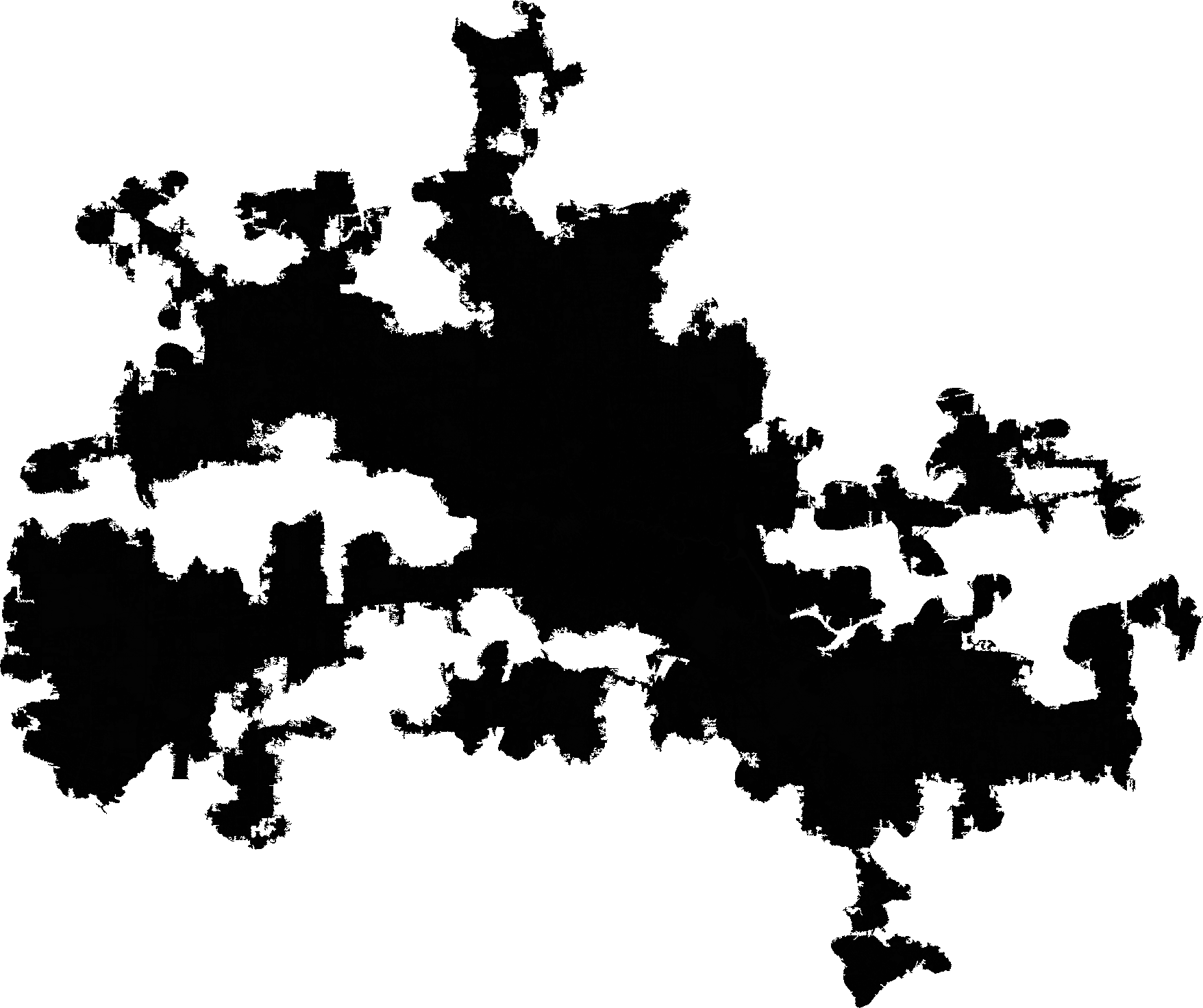

Supplement: Supplementary file 1 [file mmc1.zip › Supplementary/Landcover/Houston_1990.tif]

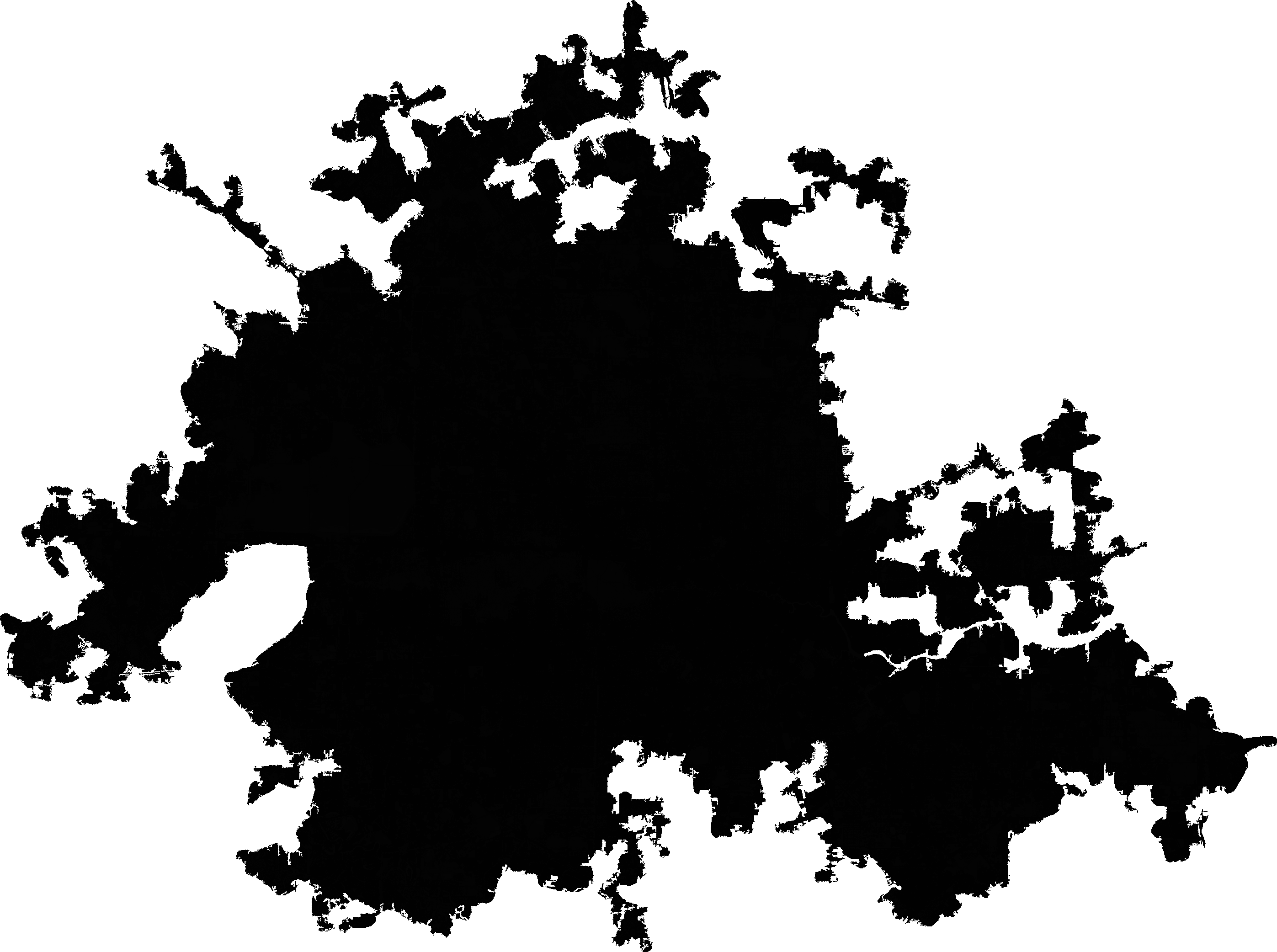

Supplement: Supplementary file 1 [file mmc1.zip › Supplementary/Landcover/Houston_2015.tif]

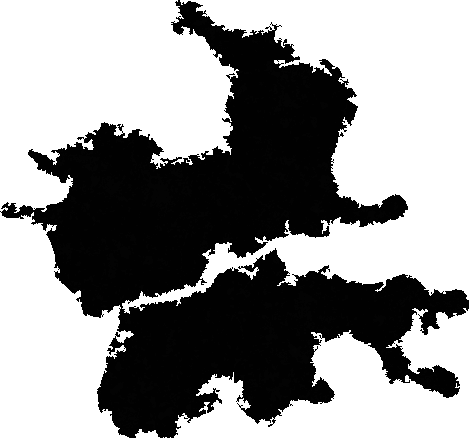

Supplement: Supplementary file 1 [file mmc1.zip › Supplementary/Landcover/Hyderabad_1990.tif]

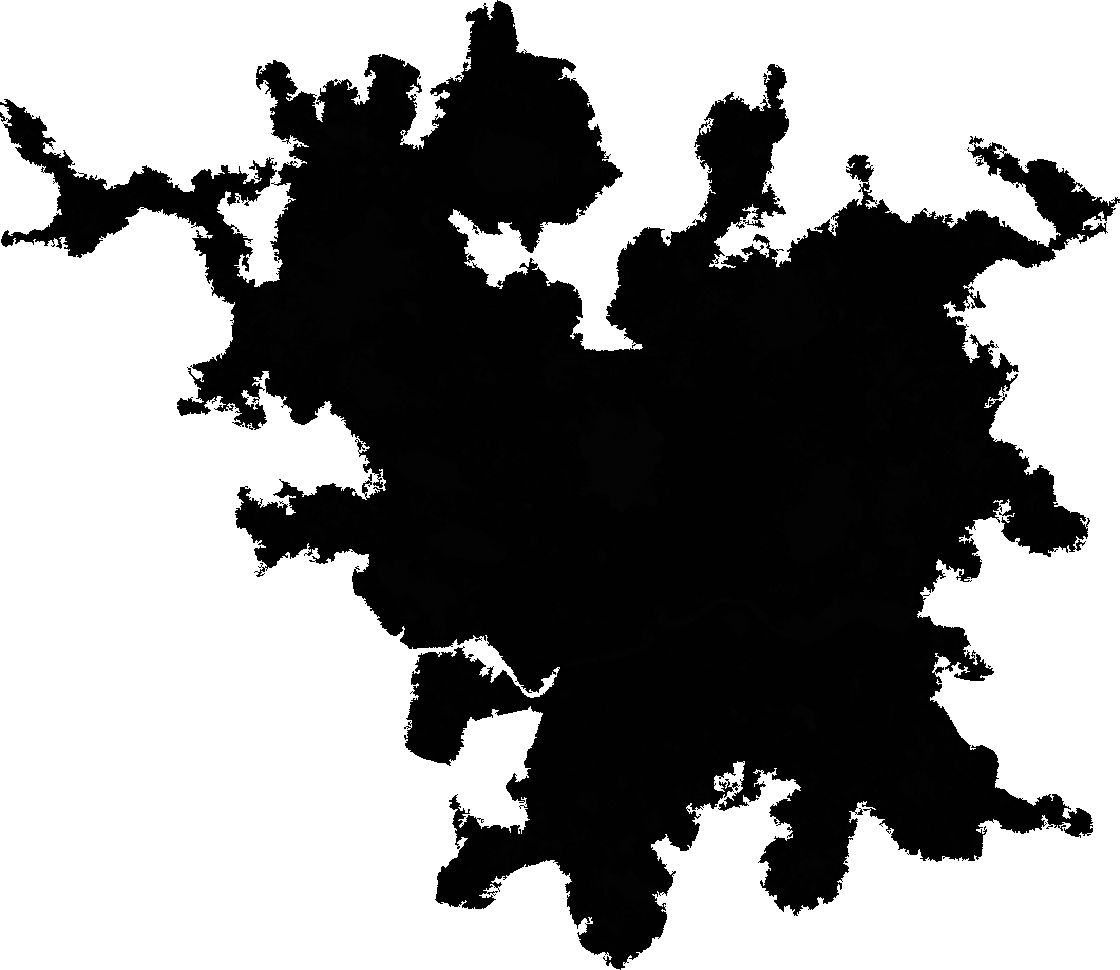

Supplement: Supplementary file 1 [file mmc1.zip › Supplementary/Landcover/Hyderabad_2015.tif]

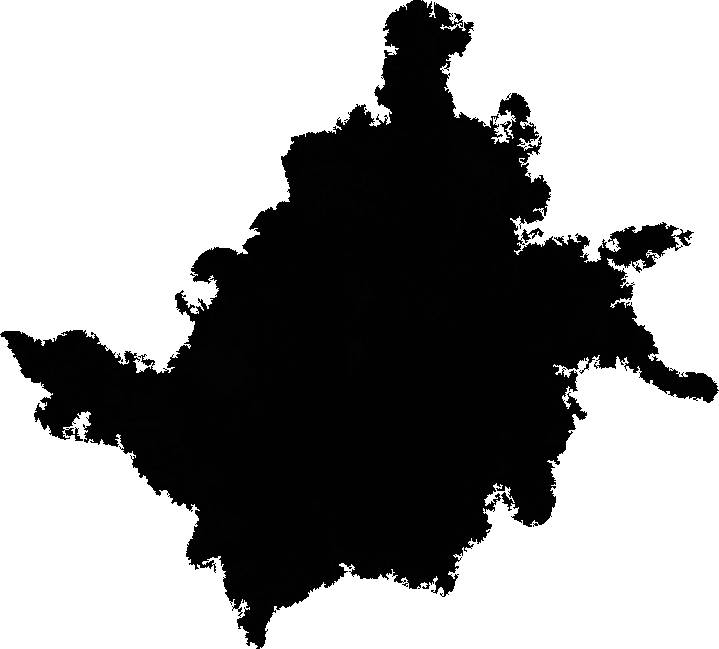

Supplement: Supplementary file 1 [file mmc1.zip › Supplementary/Landcover/Ibadan_1990.tif]

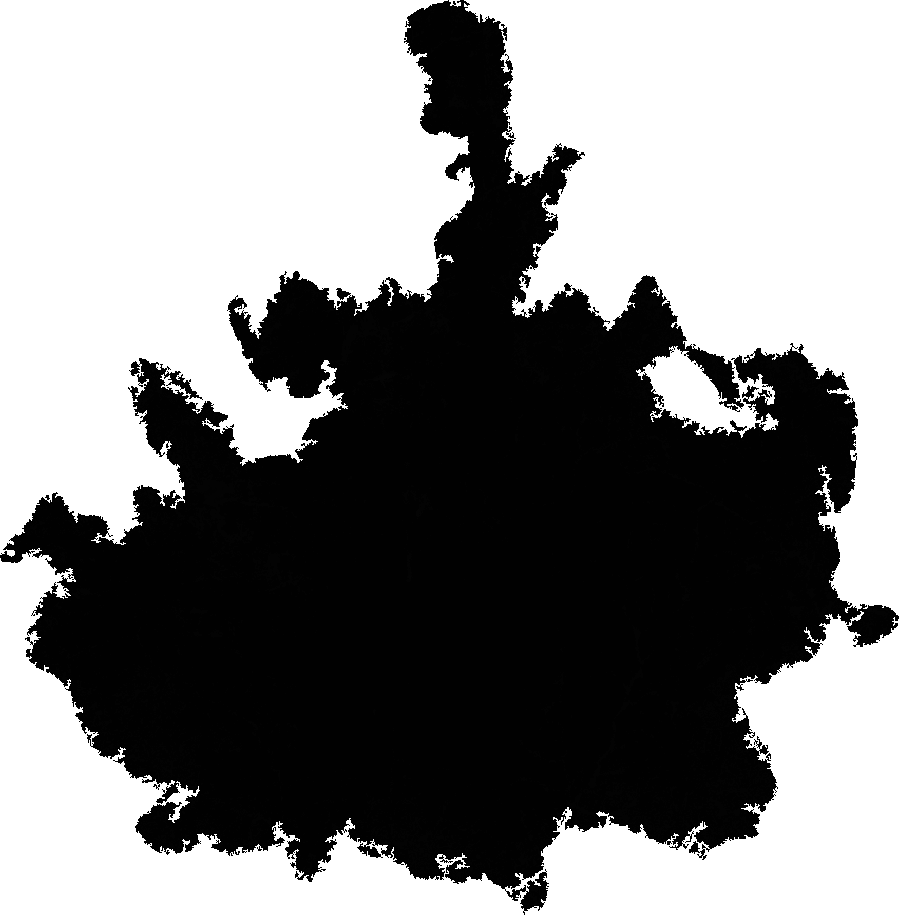

Supplement: Supplementary file 1 [file mmc1.zip › Supplementary/Landcover/Ibadan_2015.tif]

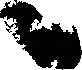

Supplement: Supplementary file 1 [file mmc1.zip › Supplementary/Landcover/Ilheus_1990.tif]

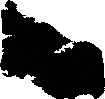

Supplement: Supplementary file 1 [file mmc1.zip › Supplementary/Landcover/Ilheus_2015.tif]

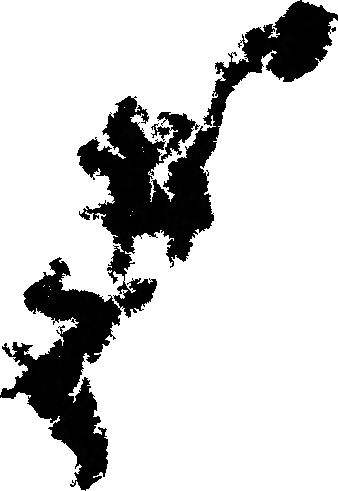

Supplement: Supplementary file 1 [file mmc1.zip › Supplementary/Landcover/Ipoh_1990.tif]

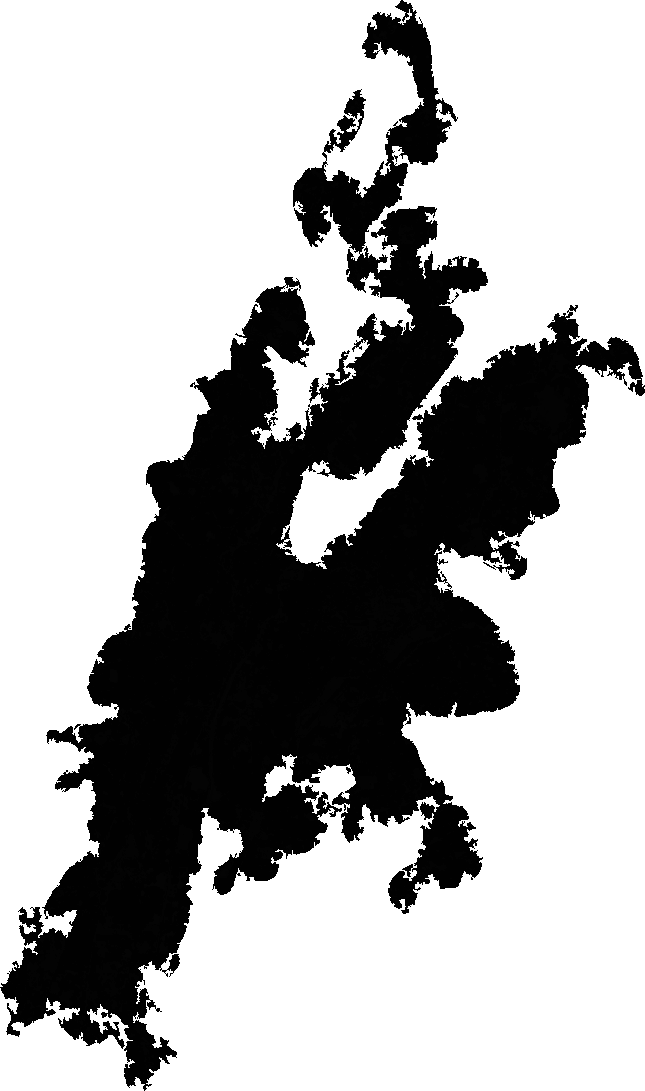

Supplement: Supplementary file 1 [file mmc1.zip › Supplementary/Landcover/Ipoh_2015.tif]

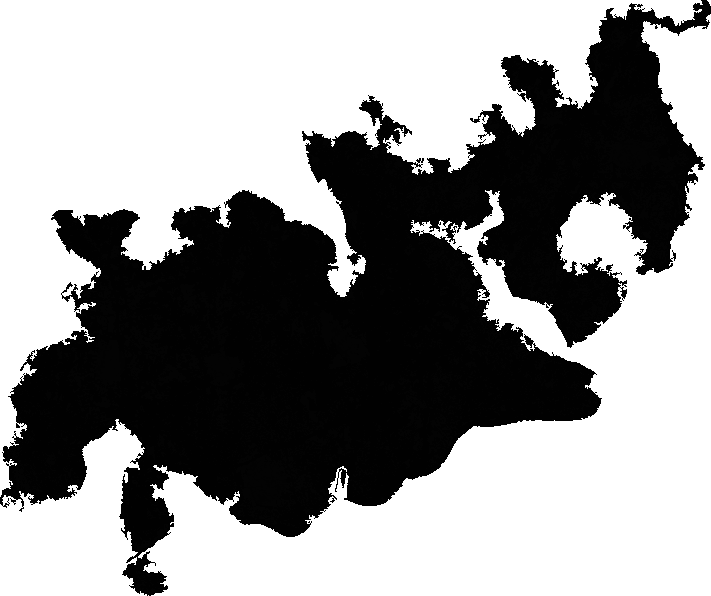

Supplement: Supplementary file 1 [file mmc1.zip › Supplementary/Landcover/Istanbul_1990.tif]

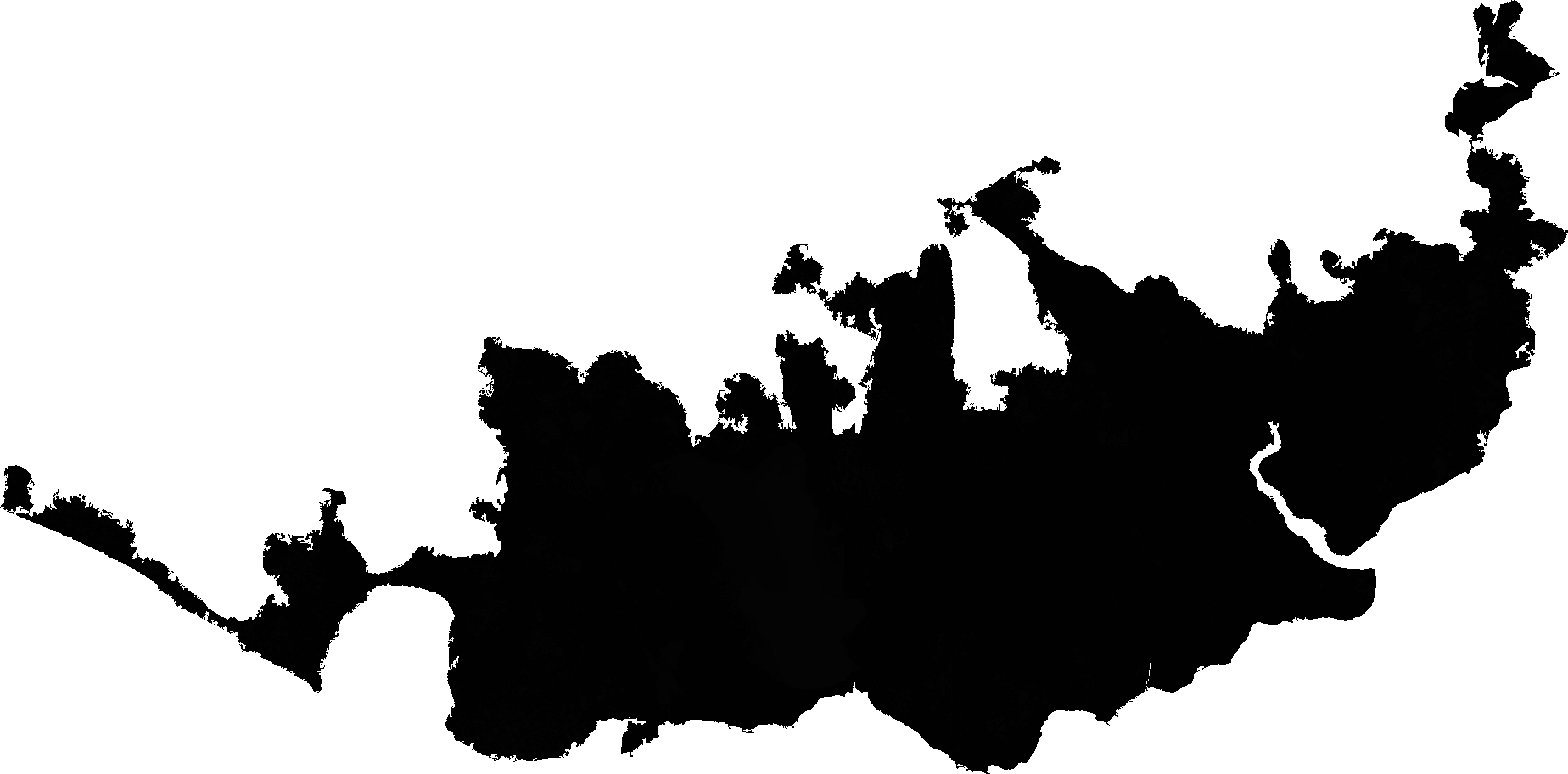

Supplement: Supplementary file 1 [file mmc1.zip › Supplementary/Landcover/Istanbul_2015.tif]

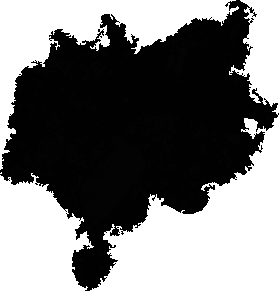

Supplement: Supplementary file 1 [file mmc1.zip › Supplementary/Landcover/Jaipur_1990.tif]

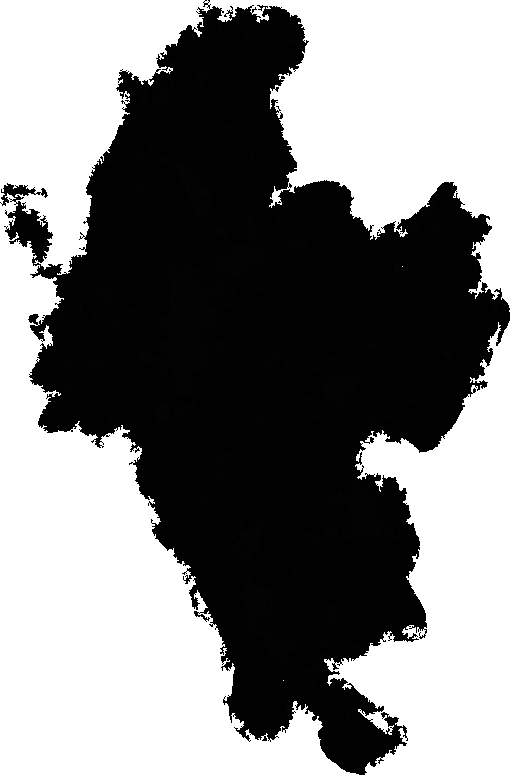

Supplement: Supplementary file 1 [file mmc1.zip › Supplementary/Landcover/Jaipur_2015.tif]

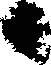

Supplement: Supplementary file 1 [file mmc1.zip › Supplementary/Landcover/Jalna_1990.tif]

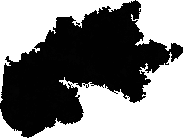

Supplement: Supplementary file 1 [file mmc1.zip › Supplementary/Landcover/Jalna_2015.tif]

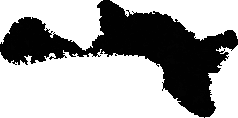

Supplement: Supplementary file 1 [file mmc1.zip › Supplementary/Landcover/Jequie_1990.tif]

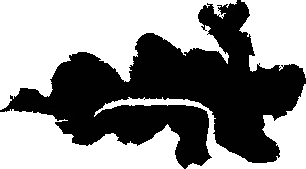

Supplement: Supplementary file 1 [file mmc1.zip › Supplementary/Landcover/Jequie_2015.tif]

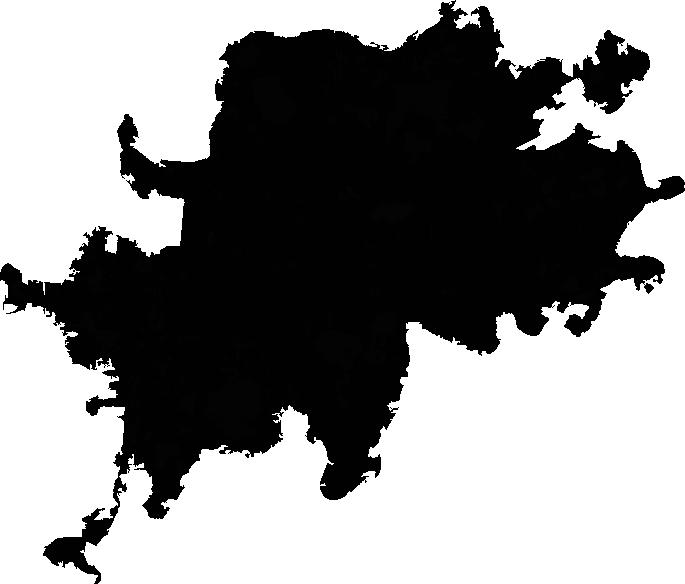

Supplement: Supplementary file 1 [file mmc1.zip › Supplementary/Landcover/Jinan_Shandong_1990.tif]

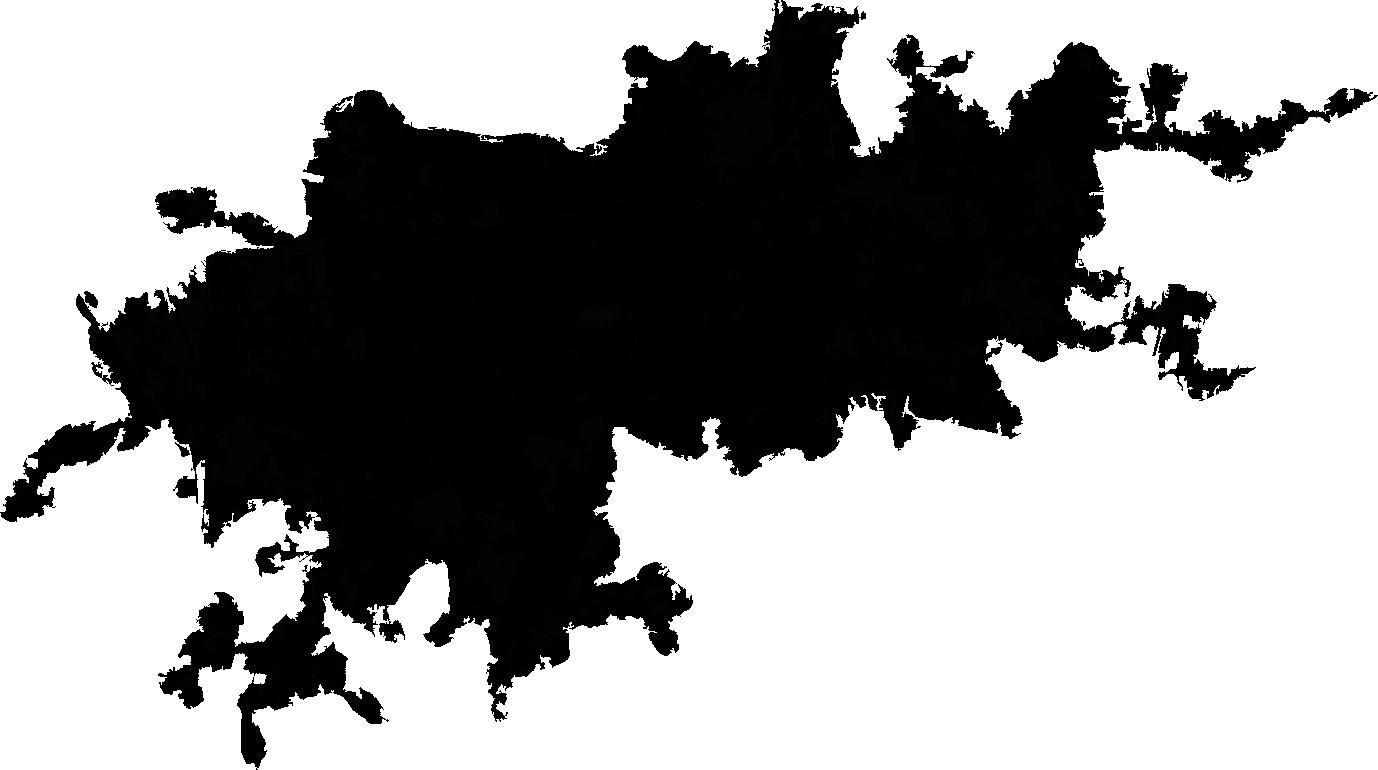

Supplement: Supplementary file 1 [file mmc1.zip › Supplementary/Landcover/Jinan_Shandong_2015.tif]

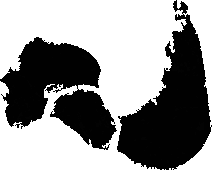

Supplement: Supplementary file 1 [file mmc1.zip › Supplementary/Landcover/Jinju_1990.tif]

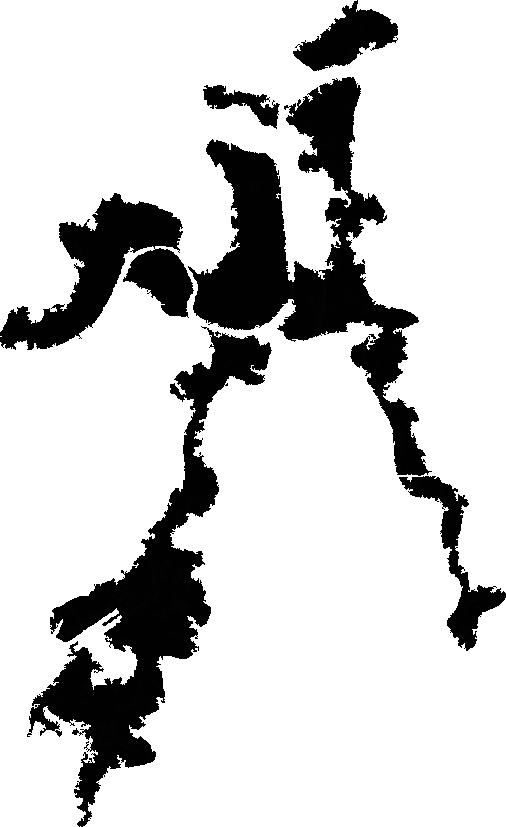

Supplement: Supplementary file 1 [file mmc1.zip › Supplementary/Landcover/Jinju_2015.tif]

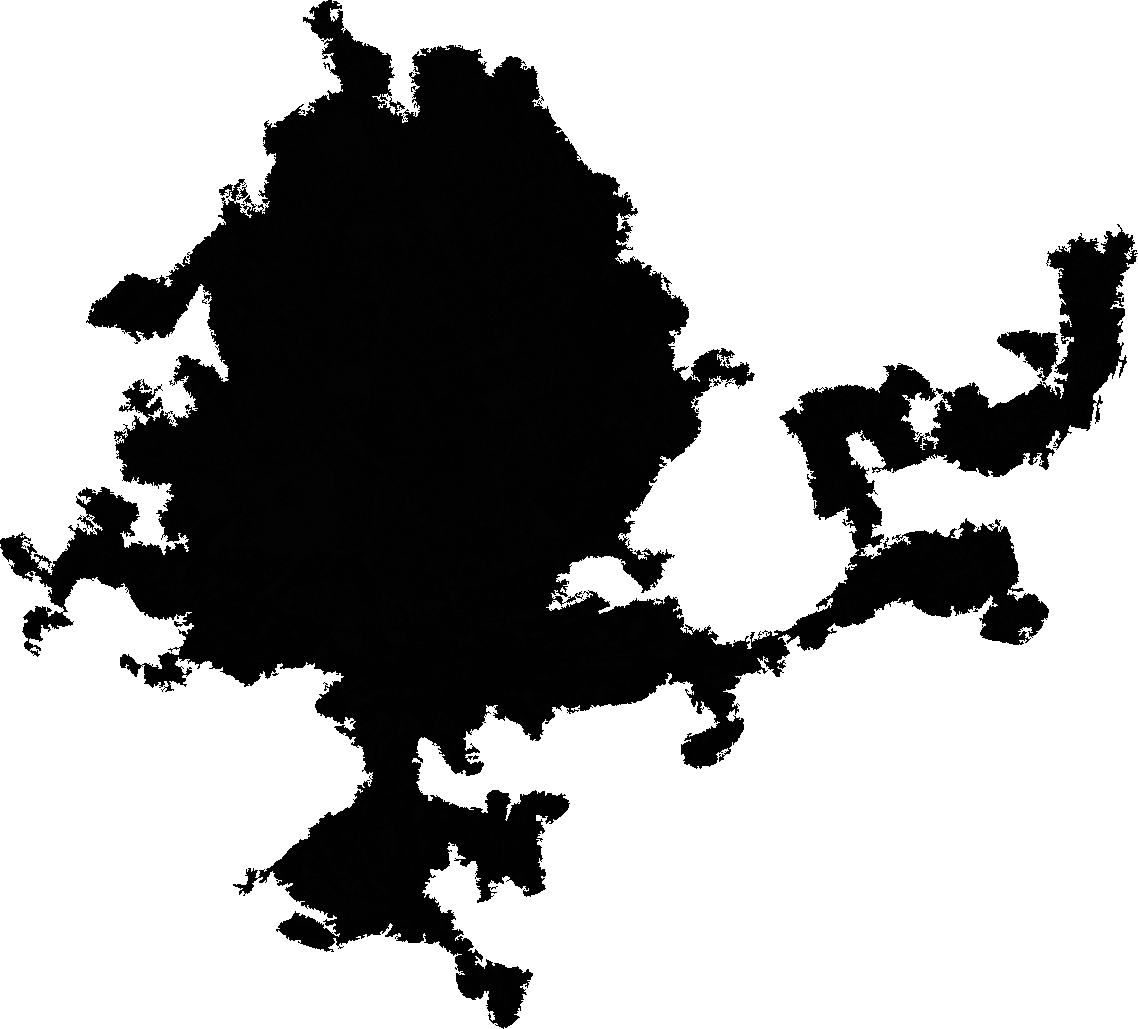

Supplement: Supplementary file 1 [file mmc1.zip › Supplementary/Landcover/Johannesburg_1990.tif]

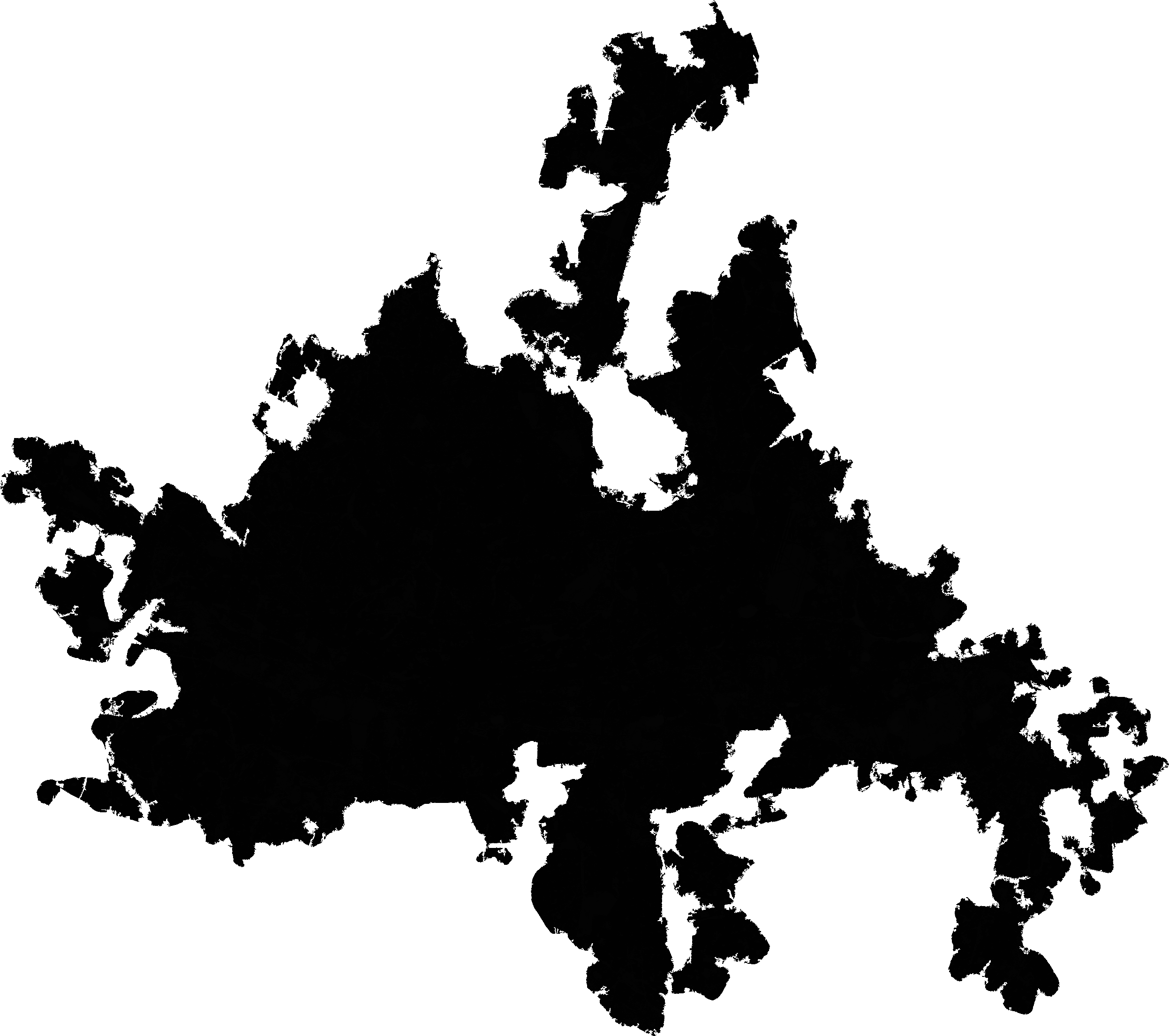

Supplement: Supplementary file 1 [file mmc1.zip › Supplementary/Landcover/Johannesburg_2015.tif]

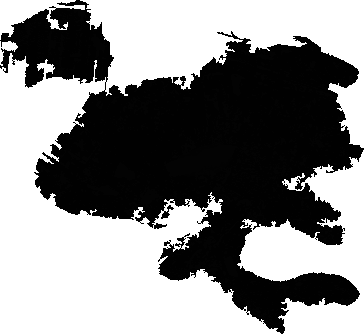

Supplement: Supplementary file 1 [file mmc1.zip › Supplementary/Landcover/Kabul_1990.tif]

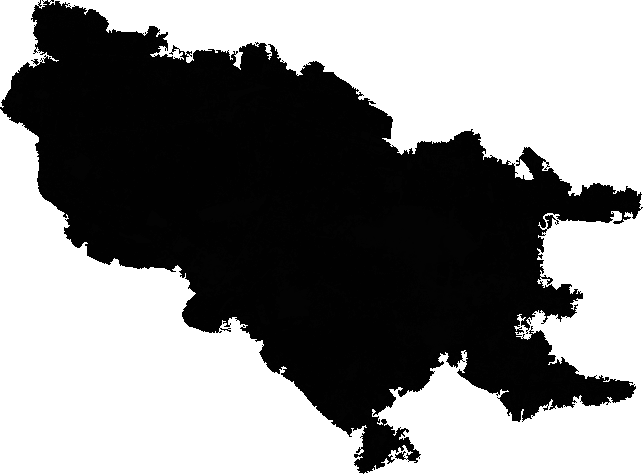

Supplement: Supplementary file 1 [file mmc1.zip › Supplementary/Landcover/Kabul_2015.tif]

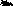

Supplement: Supplementary file 1 [file mmc1.zip › Supplementary/Landcover/Kaiping_Guangdong_1990.tif]

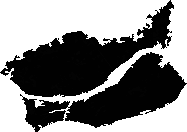

Supplement: Supplementary file 1 [file mmc1.zip › Supplementary/Landcover/Kaiping_Guangdong_2015.tif]

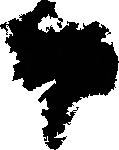

Supplement: Supplementary file 1 [file mmc1.zip › Supplementary/Landcover/Kairouan_1990.tif]

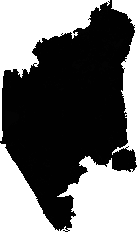

Supplement: Supplementary file 1 [file mmc1.zip › Supplementary/Landcover/Kairouan_2015.tif]

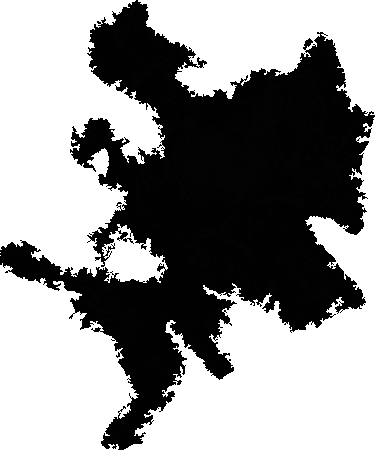

Supplement: Supplementary file 1 [file mmc1.zip › Supplementary/Landcover/Kampala_1990.tif]

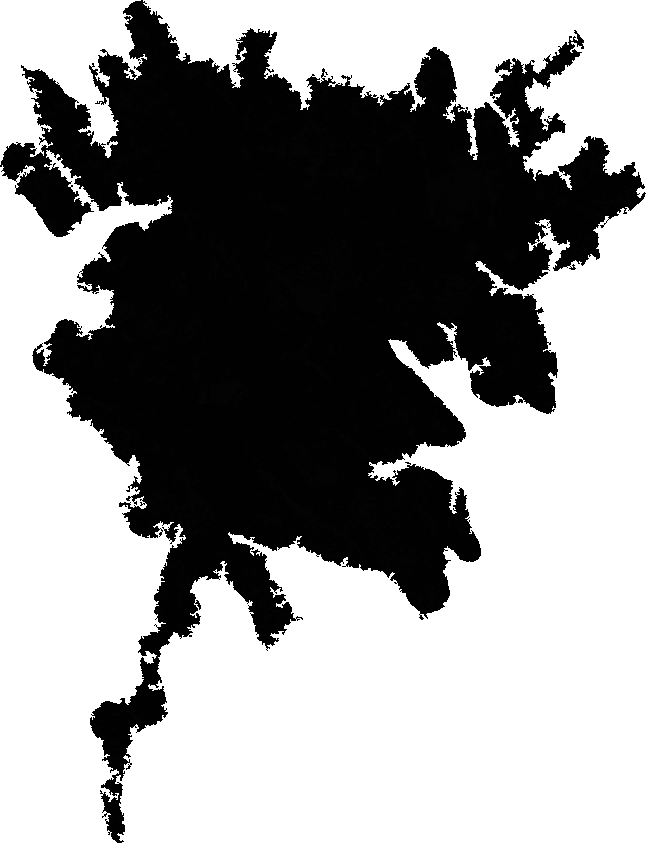

Supplement: Supplementary file 1 [file mmc1.zip › Supplementary/Landcover/Kampala_2015.tif]

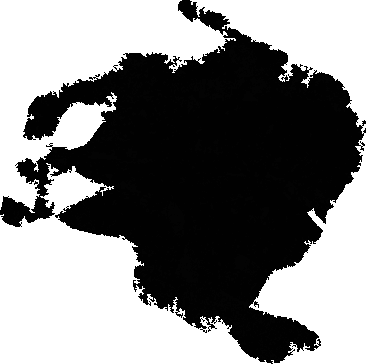

Supplement: Supplementary file 1 [file mmc1.zip › Supplementary/Landcover/Kanpur_1990.tif]

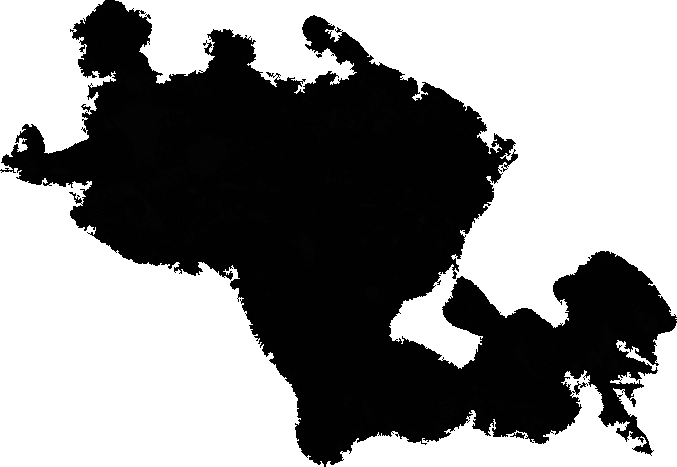

Supplement: Supplementary file 1 [file mmc1.zip › Supplementary/Landcover/Kanpur_2015.tif]

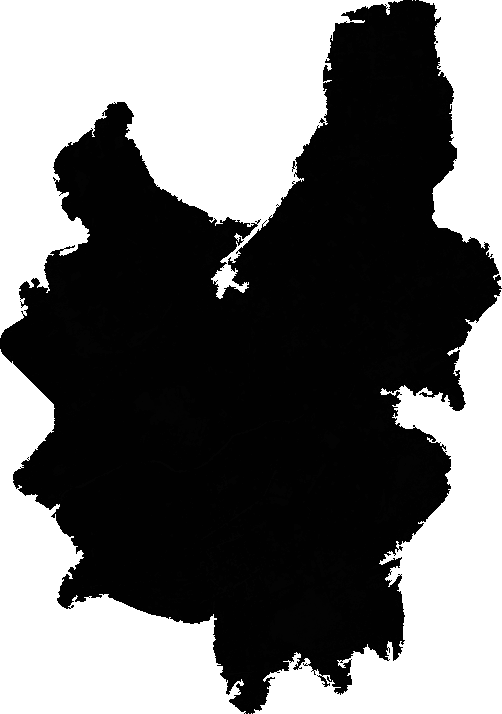

Supplement: Supplementary file 1 [file mmc1.zip › Supplementary/Landcover/Karachi_1990.tif]

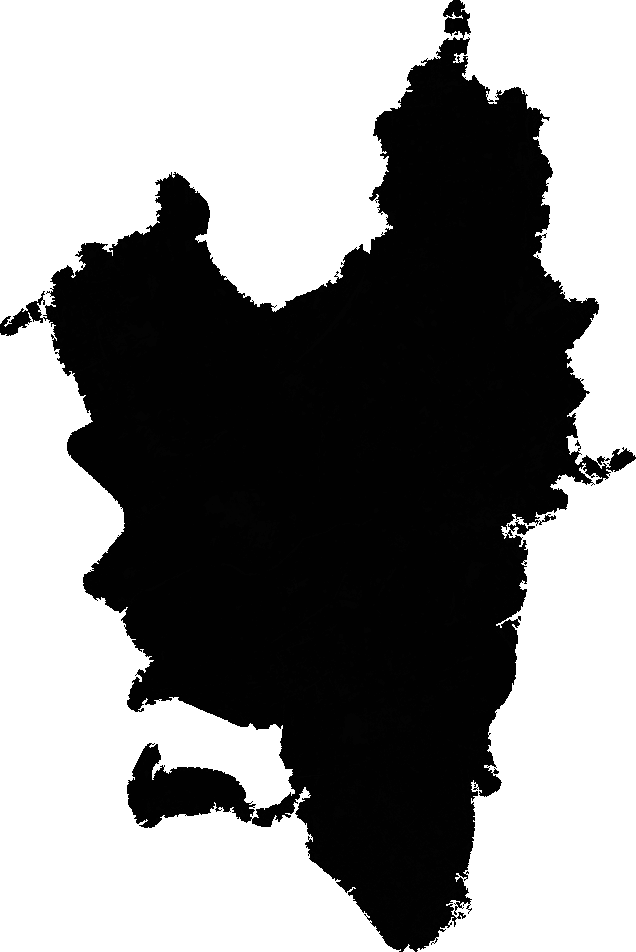

Supplement: Supplementary file 1 [file mmc1.zip › Supplementary/Landcover/Karachi_2015.tif]

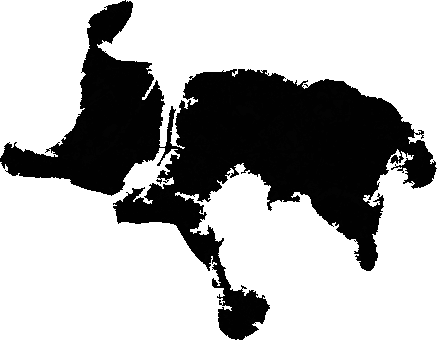

Supplement: Supplementary file 1 [file mmc1.zip › Supplementary/Landcover/Kaunas_1990.tif]

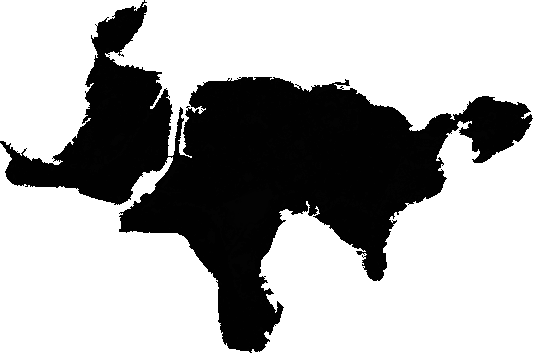

Supplement: Supplementary file 1 [file mmc1.zip › Supplementary/Landcover/Kaunas_2015.tif]

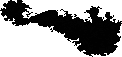

Supplement: Supplementary file 1 [file mmc1.zip › Supplementary/Landcover/Kayseri_1990.tif]

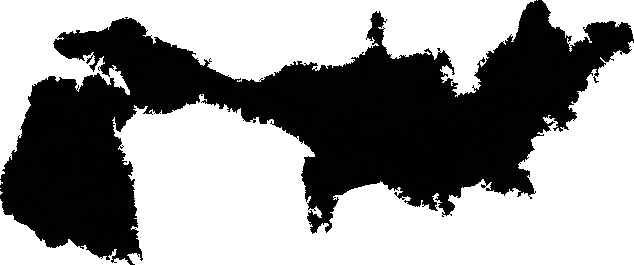

Supplement: Supplementary file 1 [file mmc1.zip › Supplementary/Landcover/Kayseri_2015.tif]

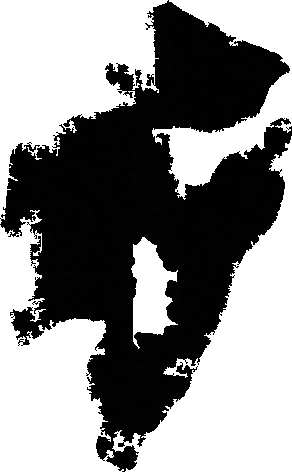

Supplement: Supplementary file 1 [file mmc1.zip › Supplementary/Landcover/Khartoum_1990.tif]

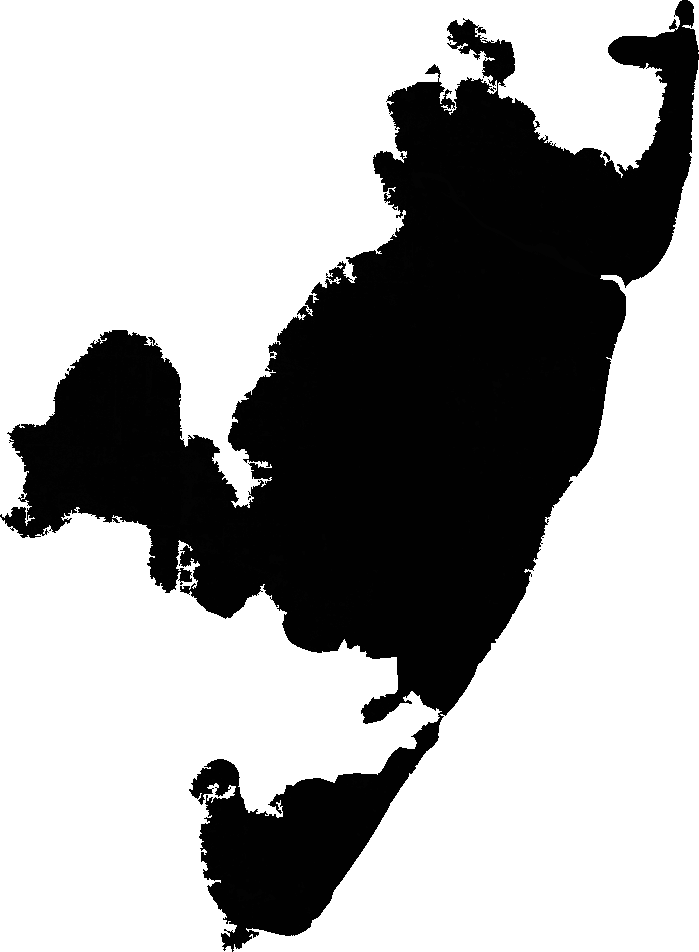

Supplement: Supplementary file 1 [file mmc1.zip › Supplementary/Landcover/Khartoum_2015.tif]

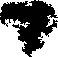

Supplement: Supplementary file 1 [file mmc1.zip › Supplementary/Landcover/Kigali_1990.tif]

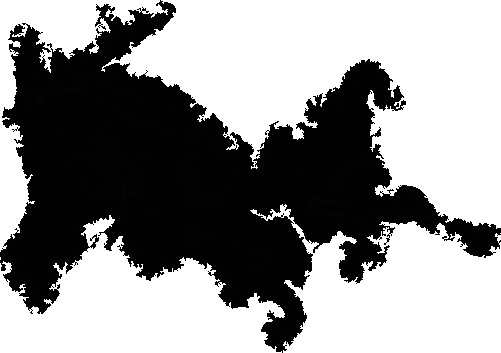

Supplement: Supplementary file 1 [file mmc1.zip › Supplementary/Landcover/Kigali_2015.tif]

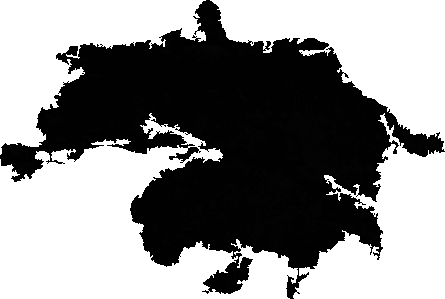

Supplement: Supplementary file 1 [file mmc1.zip › Supplementary/Landcover/Killeen_1990.tif]

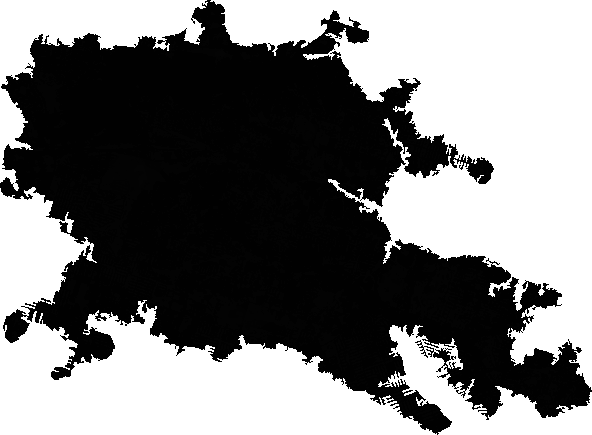

Supplement: Supplementary file 1 [file mmc1.zip › Supplementary/Landcover/Killeen_2015.tif]

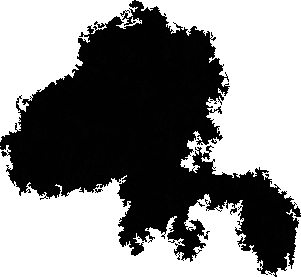

Supplement: Supplementary file 1 [file mmc1.zip › Supplementary/Landcover/Kinshasa_1990.tif]

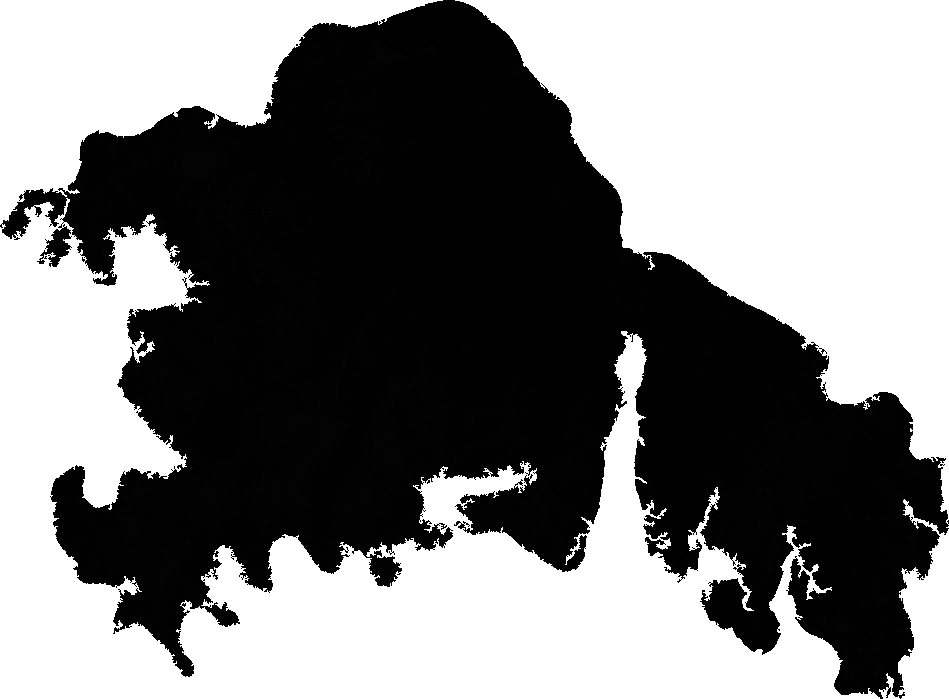

Supplement: Supplementary file 1 [file mmc1.zip › Supplementary/Landcover/Kinshasa_2015.tif]

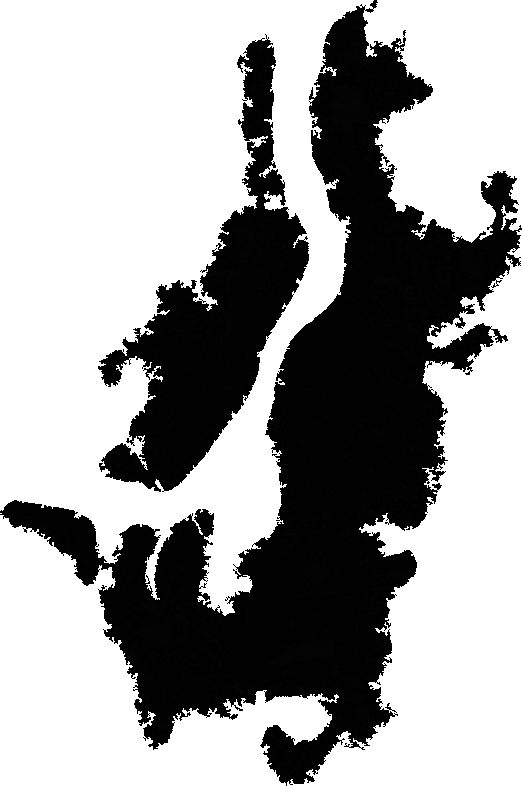

Supplement: Supplementary file 1 [file mmc1.zip › Supplementary/Landcover/Kolkata_1990.tif]

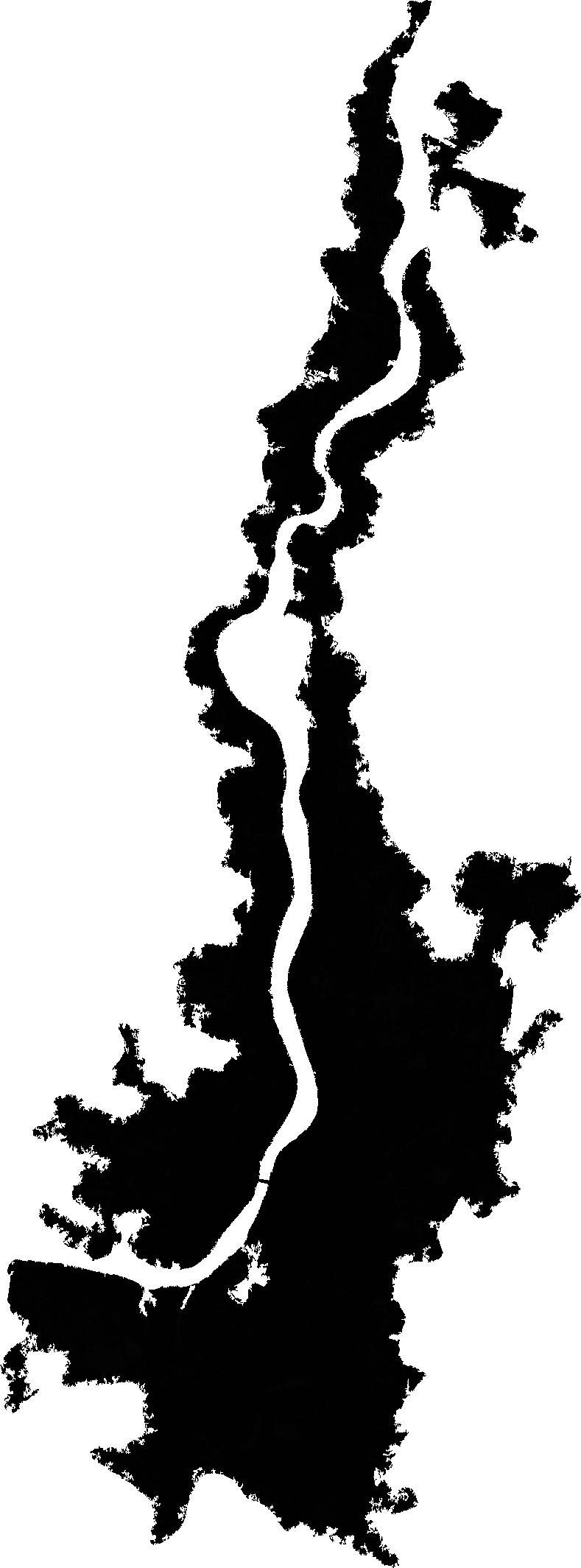

Supplement: Supplementary file 1 [file mmc1.zip › Supplementary/Landcover/Kolkata_2015.tif]

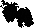

Supplement: Supplementary file 1 [file mmc1.zip › Supplementary/Landcover/Kozhikode_1990.tif]

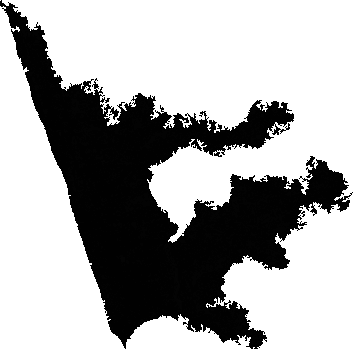

Supplement: Supplementary file 1 [file mmc1.zip › Supplementary/Landcover/Kozhikode_2015.tif]

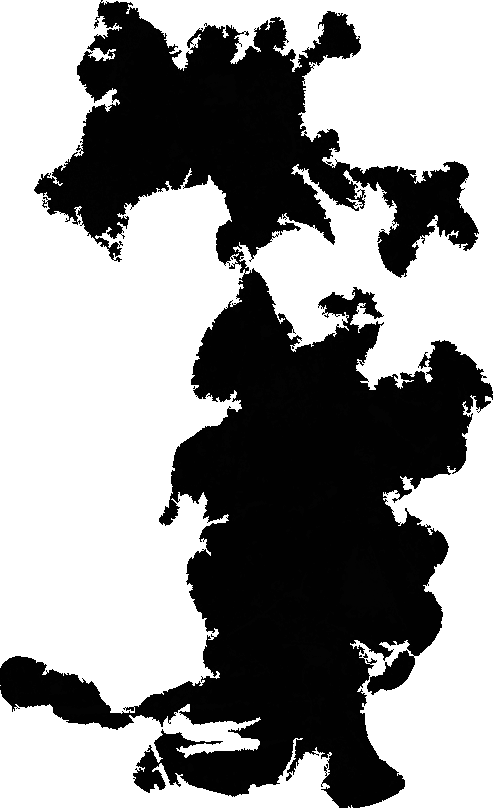

Supplement: Supplementary file 1 [file mmc1.zip › Supplementary/Landcover/Lagos_1990.tif]

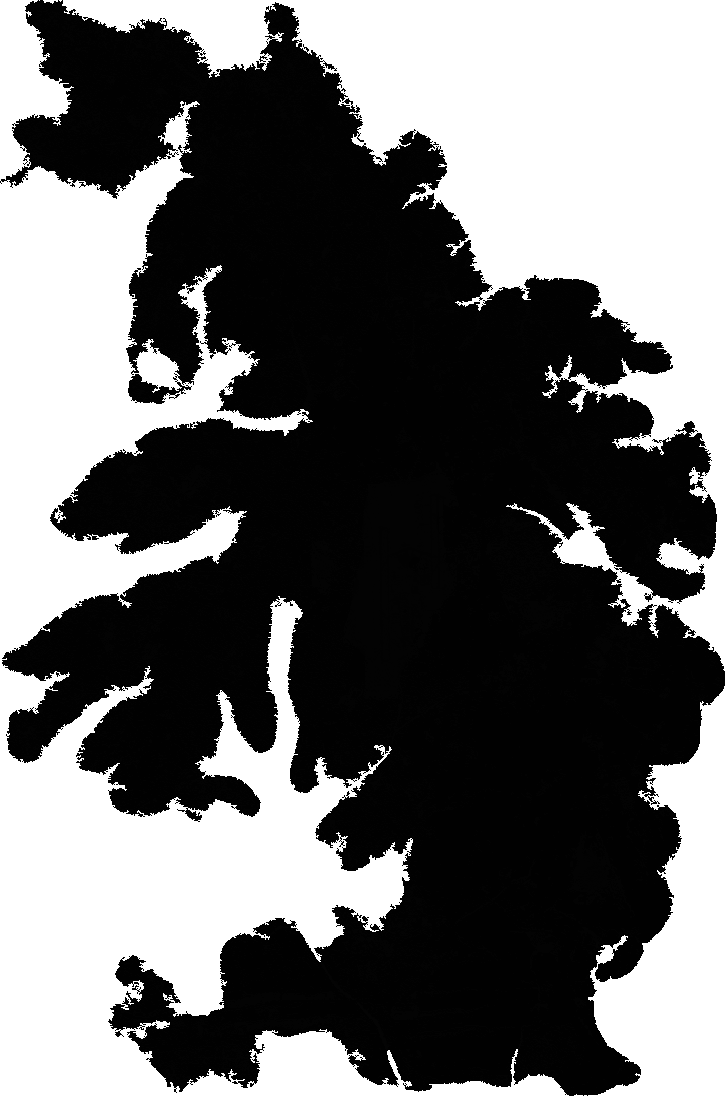

Supplement: Supplementary file 1 [file mmc1.zip › Supplementary/Landcover/Lagos_2015.tif]

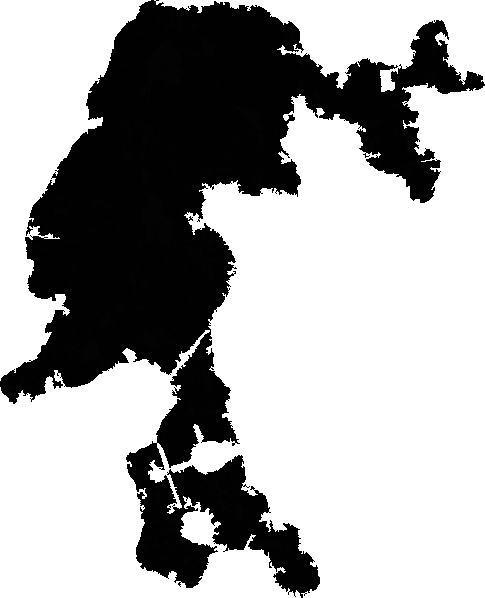

Supplement: Supplementary file 1 [file mmc1.zip › Supplementary/Landcover/Lahore_1990.tif]

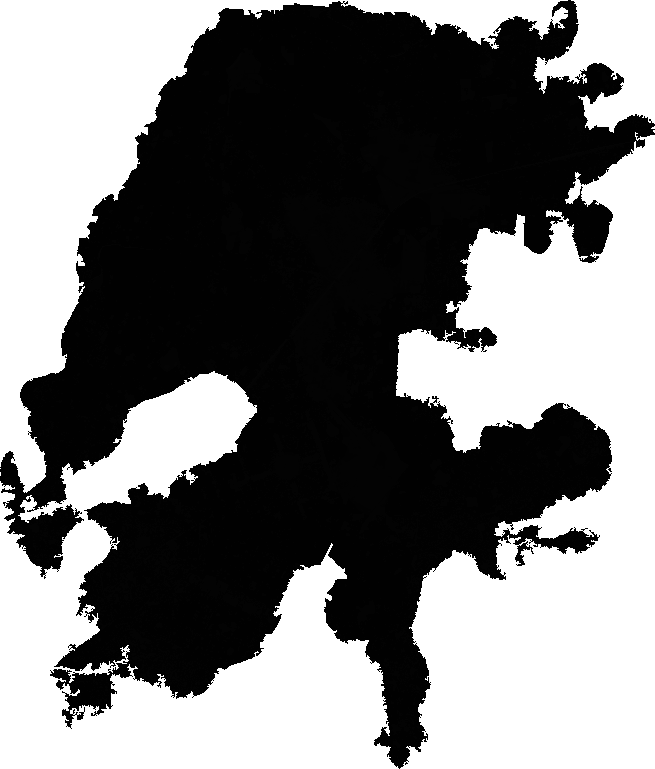

Supplement: Supplementary file 1 [file mmc1.zip › Supplementary/Landcover/Lahore_2015.tif]

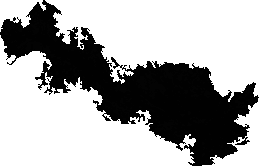

Supplement: Supplementary file 1 [file mmc1.zip › Supplementary/Landcover/Lausanne_1990.tif]

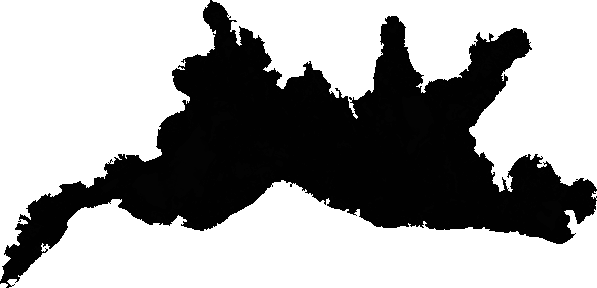

Supplement: Supplementary file 1 [file mmc1.zip › Supplementary/Landcover/Lausanne_2015.tif]

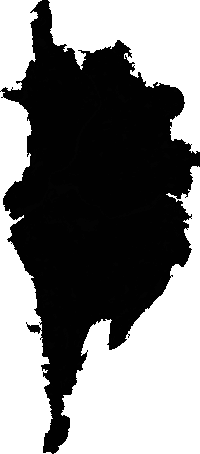

Supplement: Supplementary file 1 [file mmc1.zip › Supplementary/Landcover/Le_Mans_1990.tif]

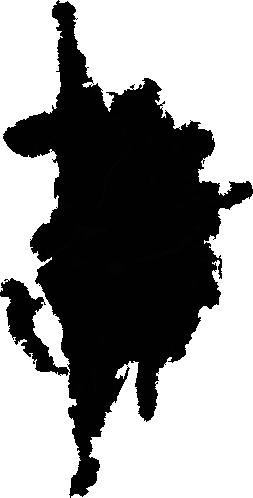

Supplement: Supplementary file 1 [file mmc1.zip › Supplementary/Landcover/Le_Mans_2015.tif]

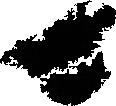

Supplement: Supplementary file 1 [file mmc1.zip › Supplementary/Landcover/Leon_1990.tif]

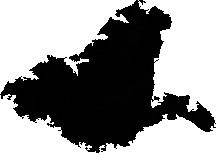

Supplement: Supplementary file 1 [file mmc1.zip › Supplementary/Landcover/Leon_2015.tif]

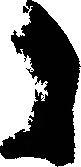

Supplement: Supplementary file 1 [file mmc1.zip › Supplementary/Landcover/Leshan_Sichuan_1990.tif]

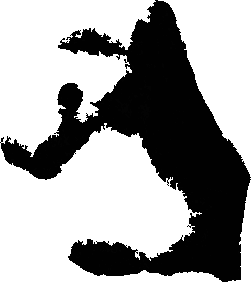

Supplement: Supplementary file 1 [file mmc1.zip › Supplementary/Landcover/Leshan_Sichuan_2015.tif]

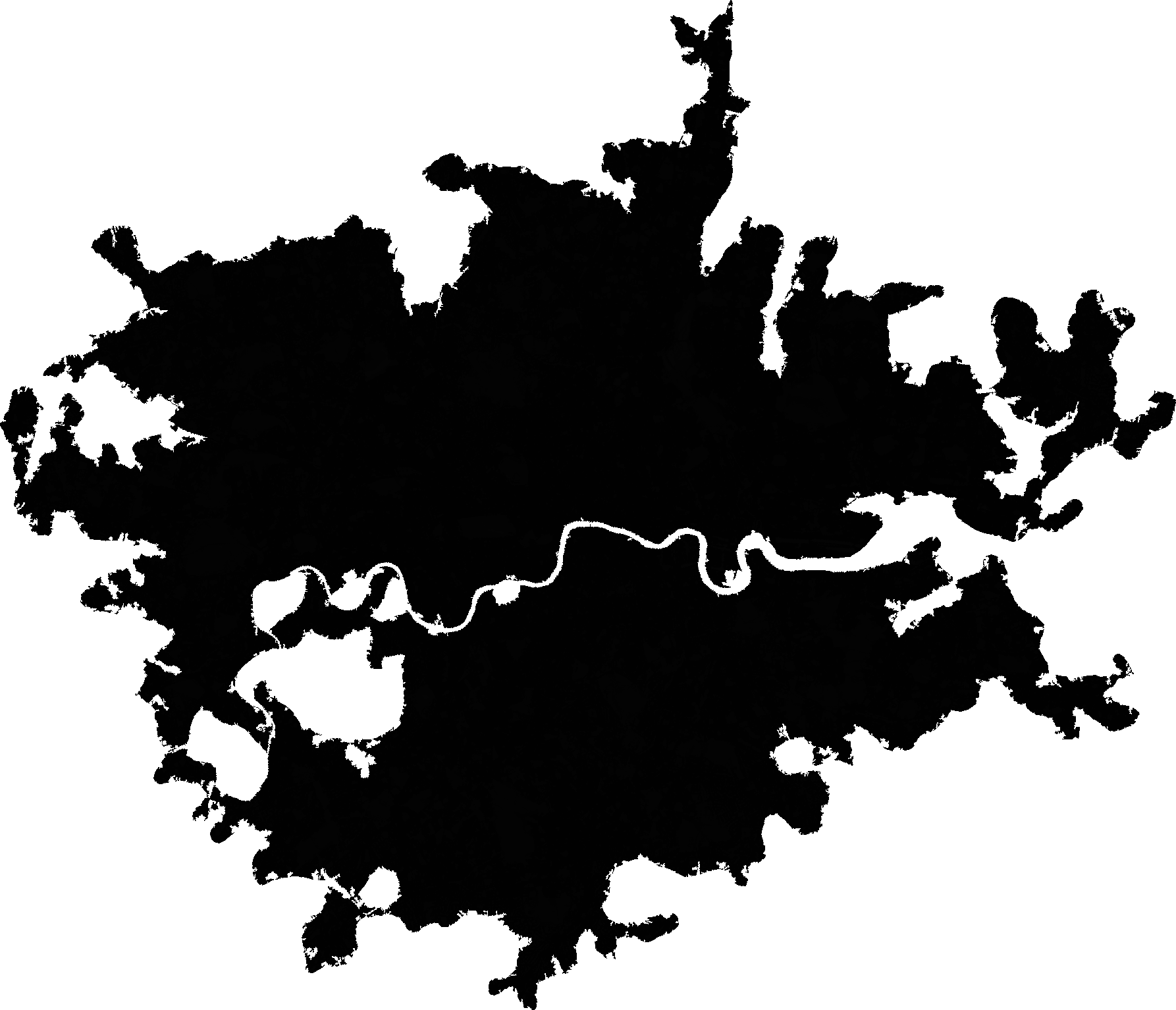

Supplement: Supplementary file 1 [file mmc1.zip › Supplementary/Landcover/London_1990.tif]
