# Supplementary figures and images for: Urban form datasets of 194 cities delineated based on the contiguous urban fabric for 1990 and 2015 (part 1 of 2)
Source: Data Brief. 2020 Oct 6;33:106369. doi: 10.1016/j.dib.2020.106369 (PMC7569292; doi:10.1016/j.dib.2020.106369)

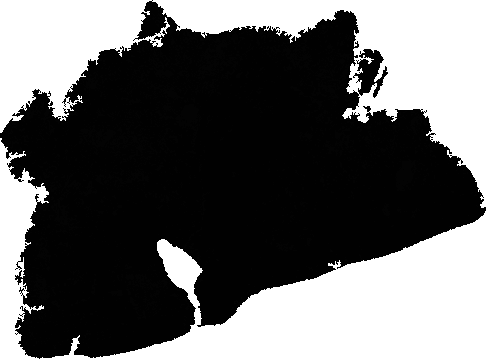

Supplement: Supplementary file 1 [file mmc1.zip › Supplementary/Landcover/Accra_1990.tif]

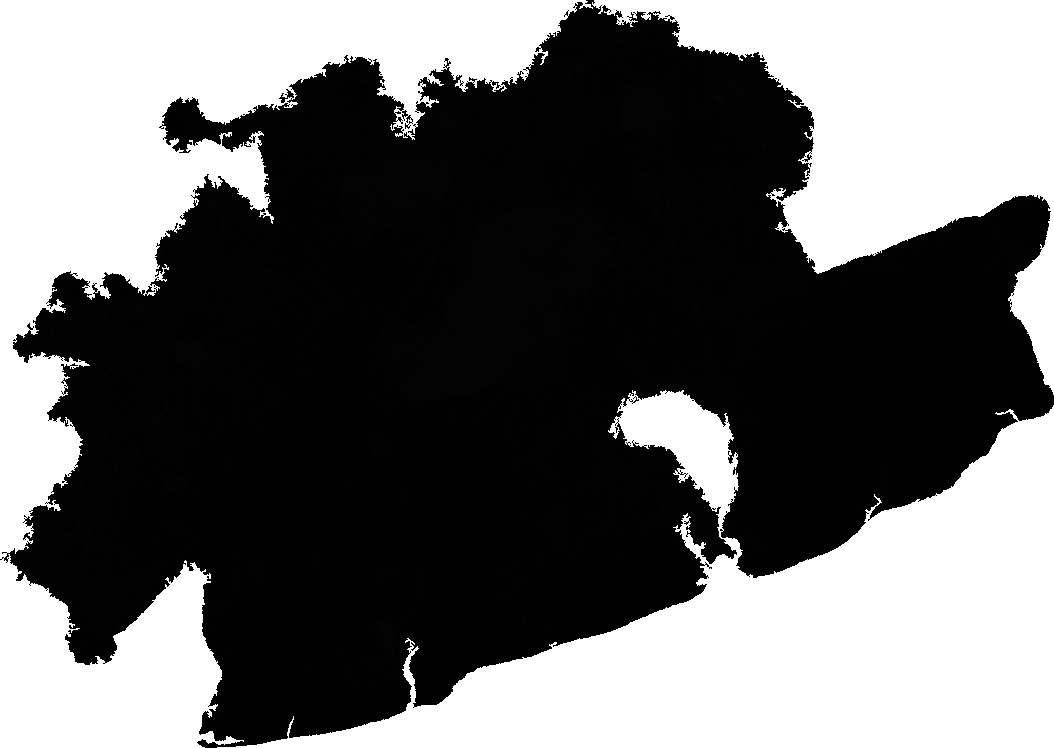

Supplement: Supplementary file 1 [file mmc1.zip › Supplementary/Landcover/Accra_2015.tif]

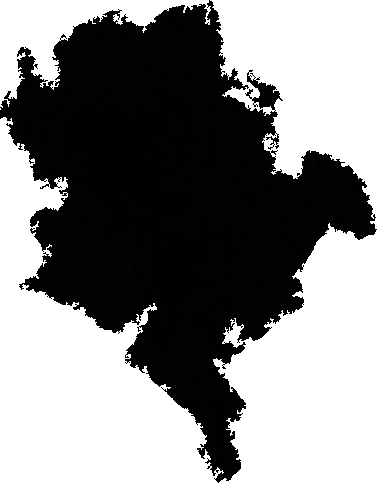

Supplement: Supplementary file 1 [file mmc1.zip › Supplementary/Landcover/Addis_Ababa_1990.tif]

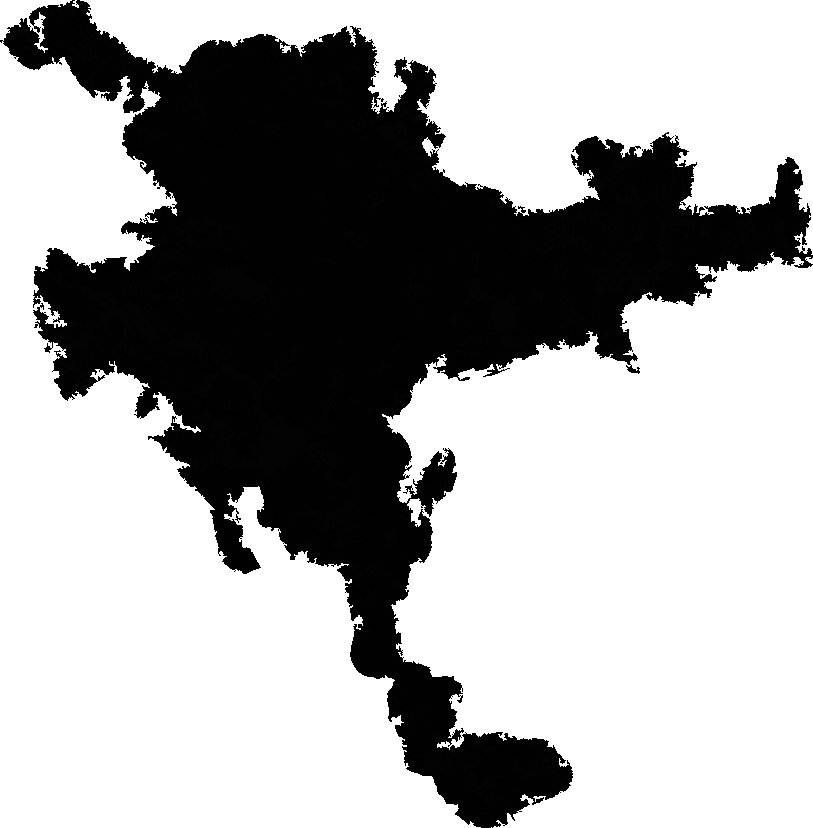

Supplement: Supplementary file 1 [file mmc1.zip › Supplementary/Landcover/Addis_Ababa_2015.tif]

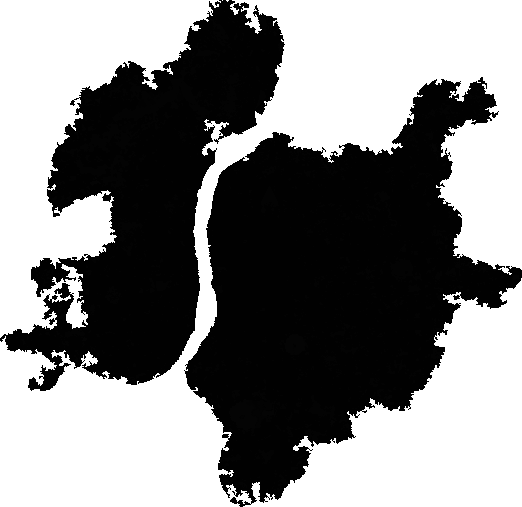

Supplement: Supplementary file 1 [file mmc1.zip › Supplementary/Landcover/Ahmedabad_1990.tif]

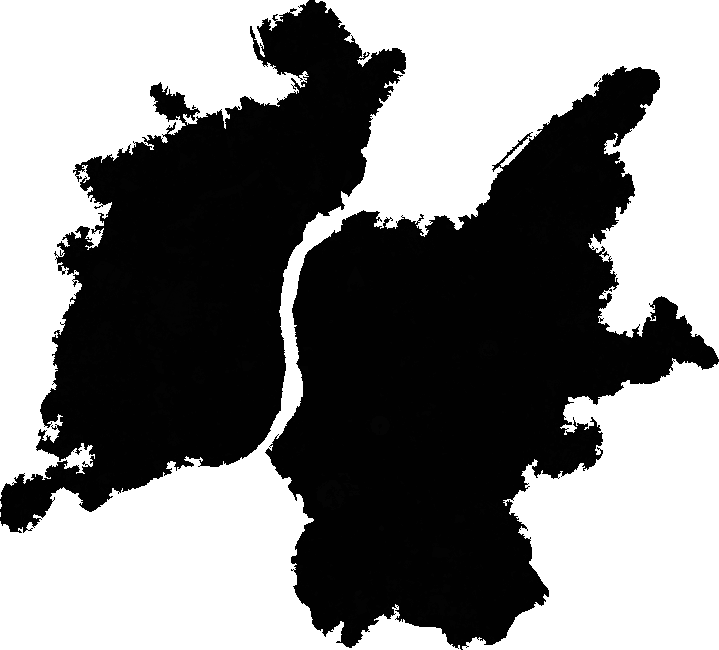

Supplement: Supplementary file 1 [file mmc1.zip › Supplementary/Landcover/Ahmedabad_2015.tif]

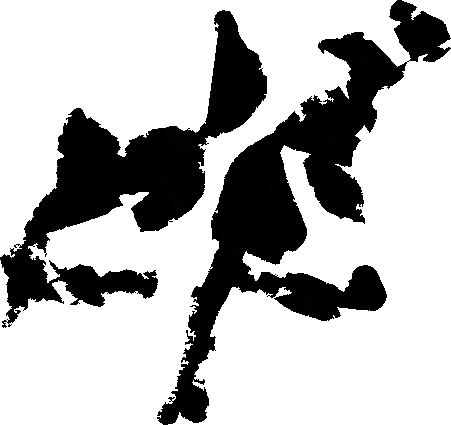

Supplement: Supplementary file 1 [file mmc1.zip › Supplementary/Landcover/Ahvaz_1990.tif]

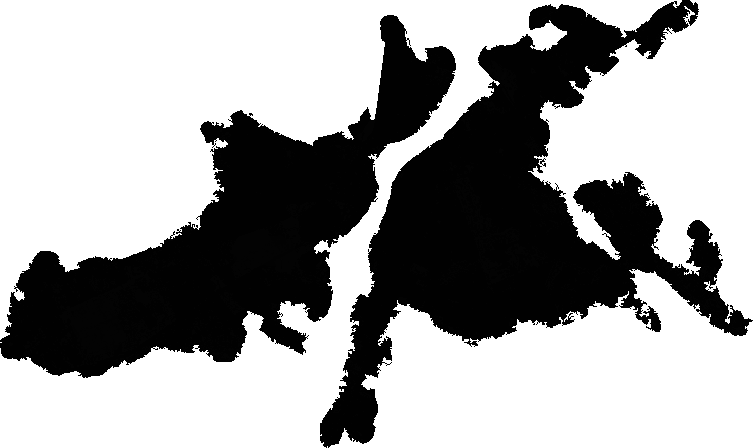

Supplement: Supplementary file 1 [file mmc1.zip › Supplementary/Landcover/Ahvaz_2015.tif]

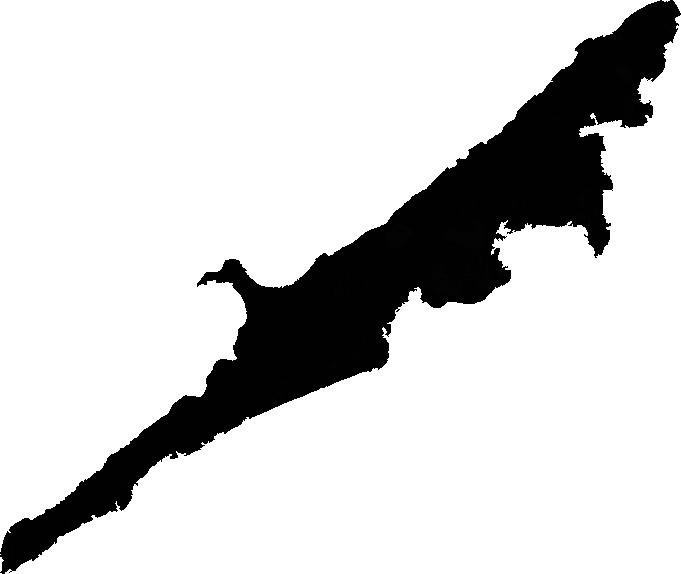

Supplement: Supplementary file 1 [file mmc1.zip › Supplementary/Landcover/Alexandria_1990.tif]

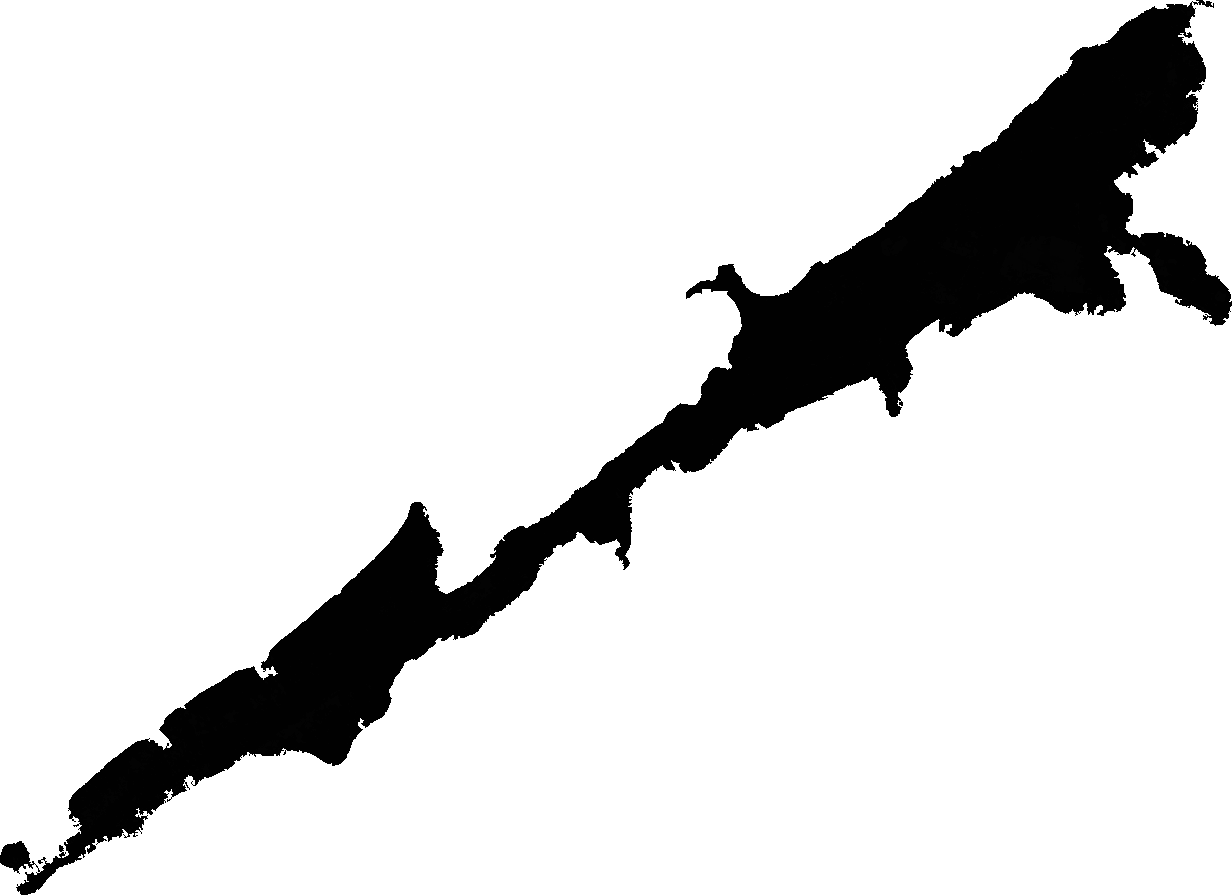

Supplement: Supplementary file 1 [file mmc1.zip › Supplementary/Landcover/Alexandria_2015.tif]

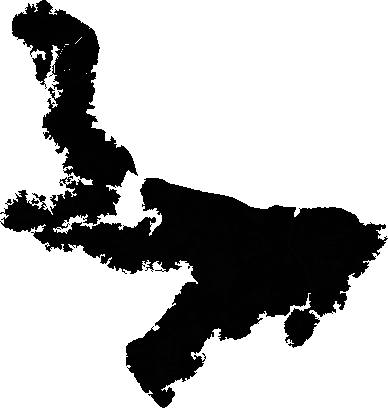

Supplement: Supplementary file 1 [file mmc1.zip › Supplementary/Landcover/Algiers_1990.tif]

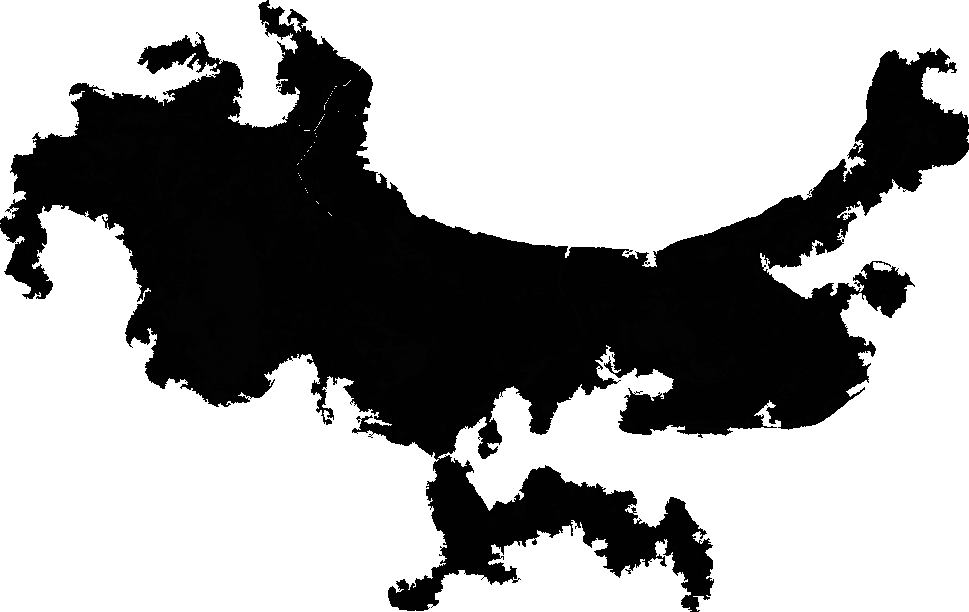

Supplement: Supplementary file 1 [file mmc1.zip › Supplementary/Landcover/Algiers_2015.tif]

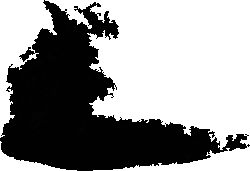

Supplement: Supplementary file 1 [file mmc1.zip › Supplementary/Landcover/Anqing_Anhui_1990.tif]

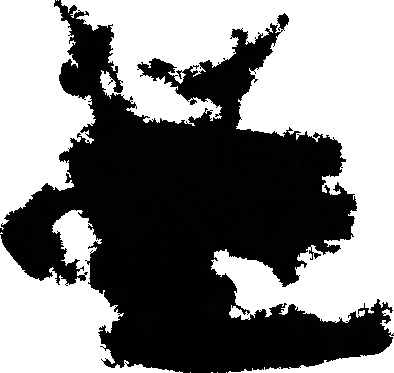

Supplement: Supplementary file 1 [file mmc1.zip › Supplementary/Landcover/Anqing_Anhui_2015.tif]

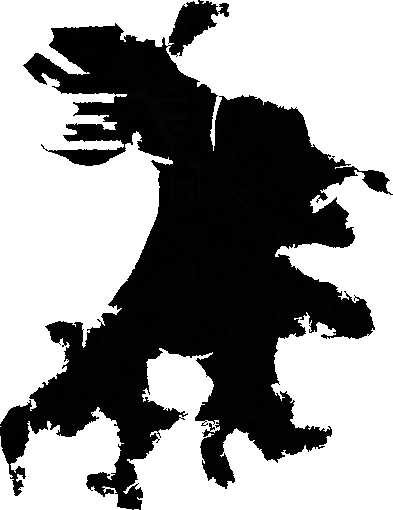

Supplement: Supplementary file 1 [file mmc1.zip › Supplementary/Landcover/Antwerp_1990.tif]

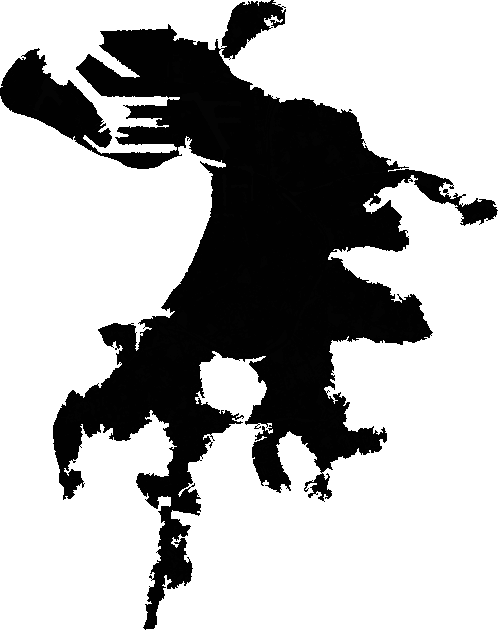

Supplement: Supplementary file 1 [file mmc1.zip › Supplementary/Landcover/Antwerp_2015.tif]

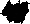

Supplement: Supplementary file 1 [file mmc1.zip › Supplementary/Landcover/Arusha_1990.tif]

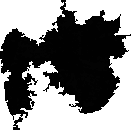

Supplement: Supplementary file 1 [file mmc1.zip › Supplementary/Landcover/Arusha_2015.tif]

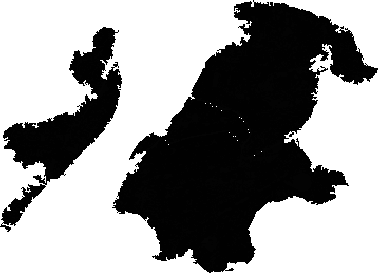

Supplement: Supplementary file 1 [file mmc1.zip › Supplementary/Landcover/Astrakhan_1990.tif]

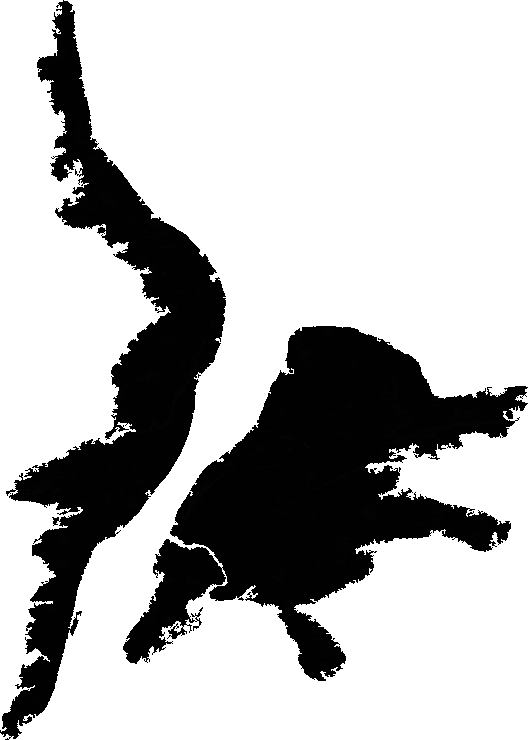

Supplement: Supplementary file 1 [file mmc1.zip › Supplementary/Landcover/Astrakhan_2015.tif]

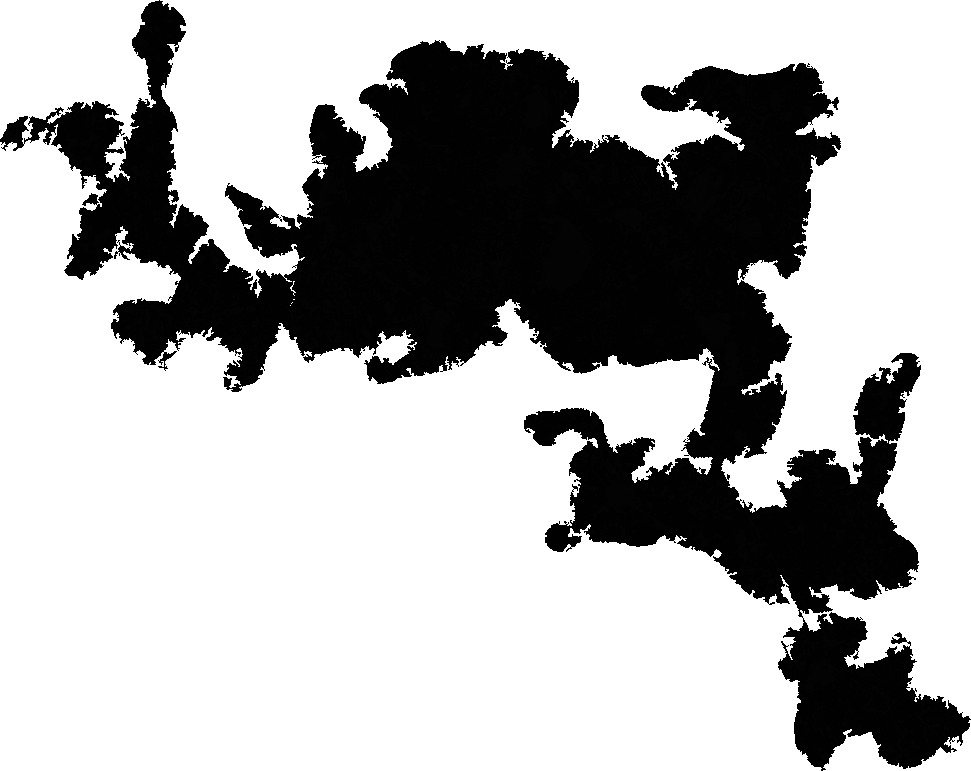

Supplement: Supplementary file 1 [file mmc1.zip › Supplementary/Landcover/Auckland_1990.tif]

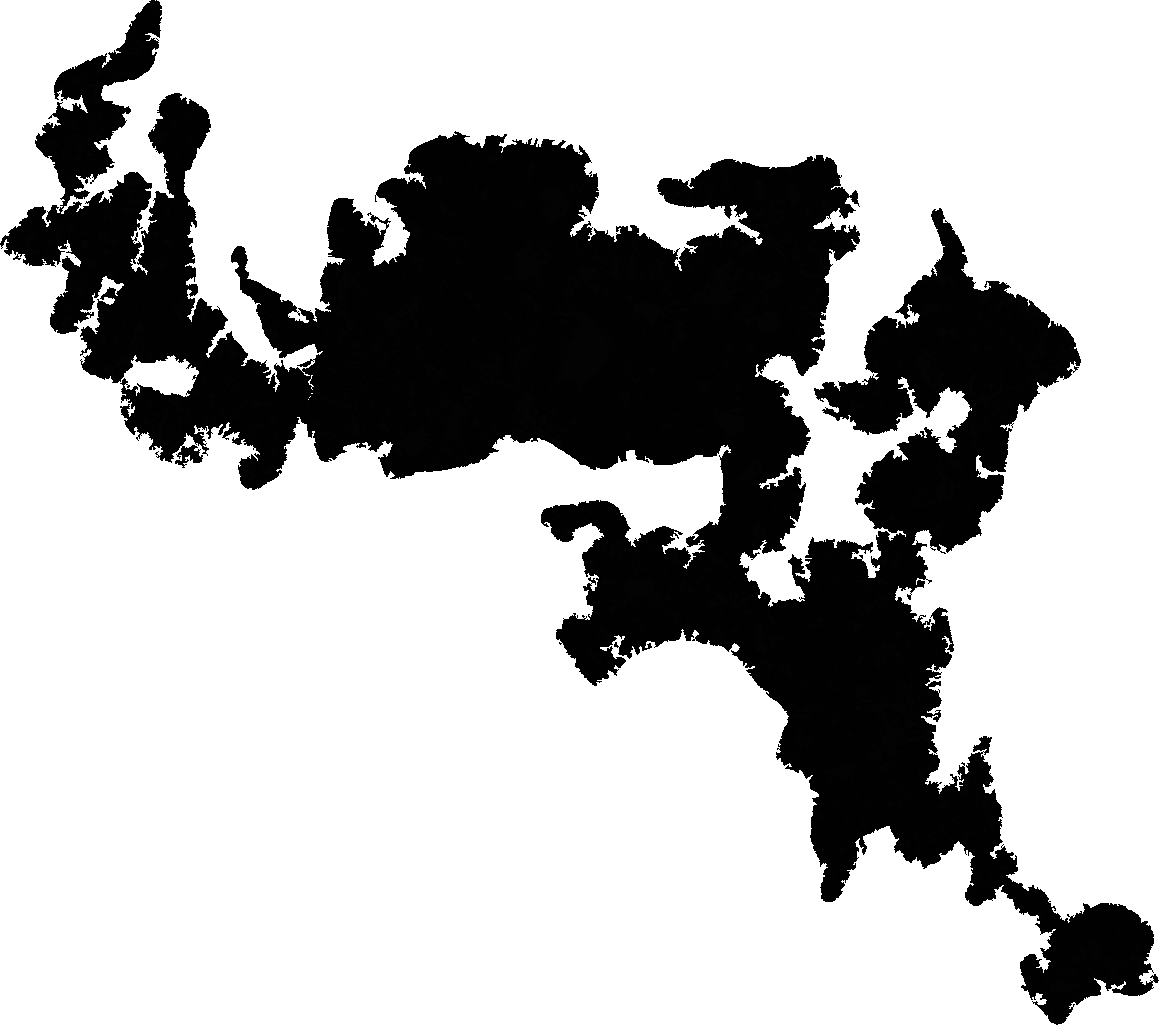

Supplement: Supplementary file 1 [file mmc1.zip › Supplementary/Landcover/Auckland_2015.tif]

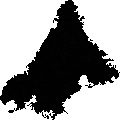

Supplement: Supplementary file 1 [file mmc1.zip › Supplementary/Landcover/Bacolod_1990.tif]

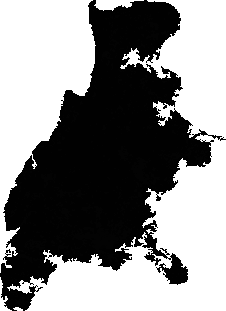

Supplement: Supplementary file 1 [file mmc1.zip › Supplementary/Landcover/Bacolod_2015.tif]

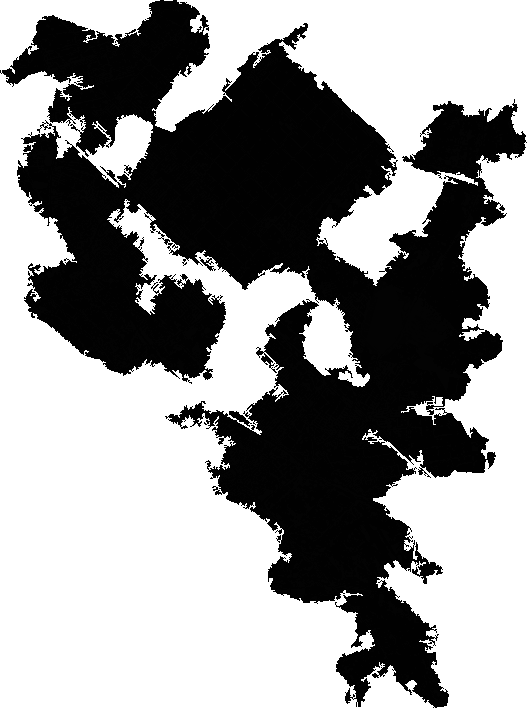

Supplement: Supplementary file 1 [file mmc1.zip › Supplementary/Landcover/Baghdad_1990.tif]

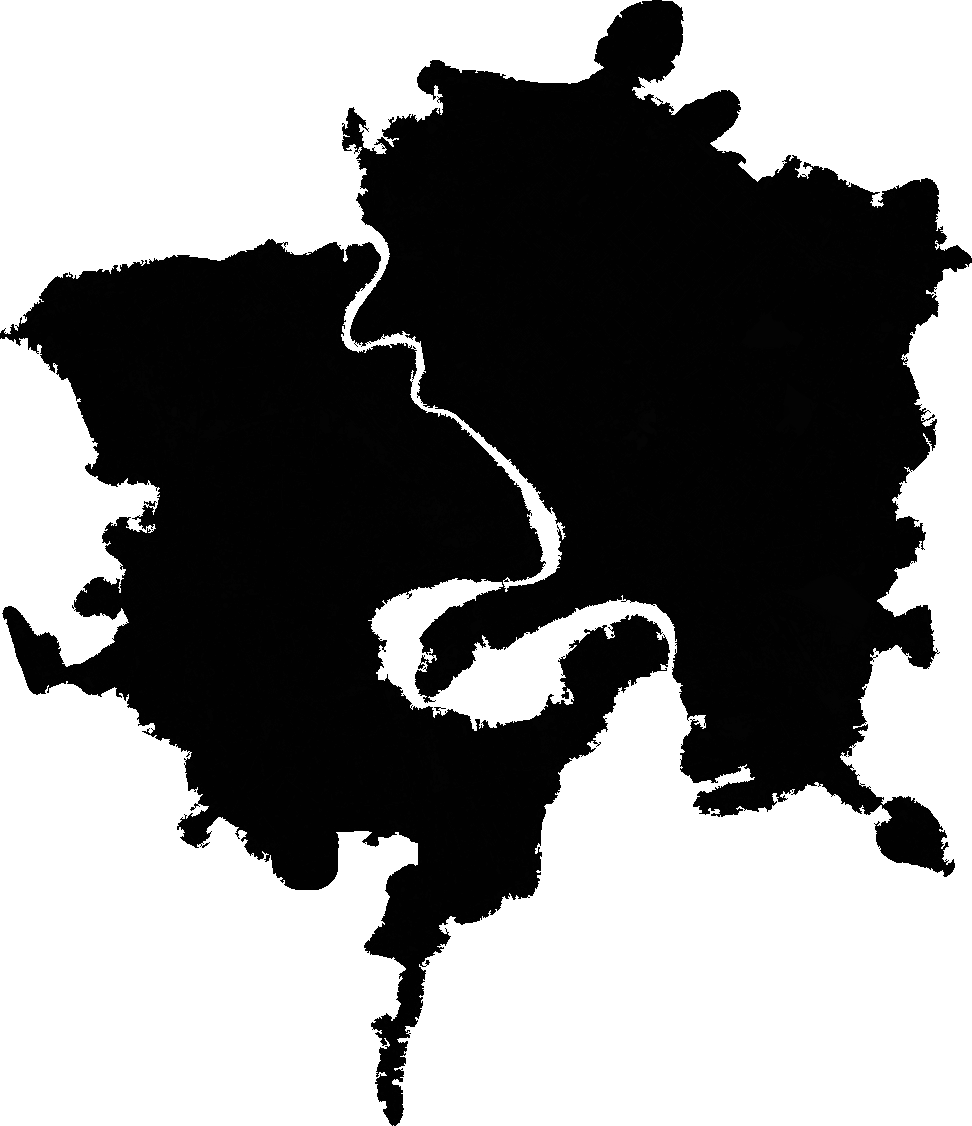

Supplement: Supplementary file 1 [file mmc1.zip › Supplementary/Landcover/Baghdad_2015.tif]

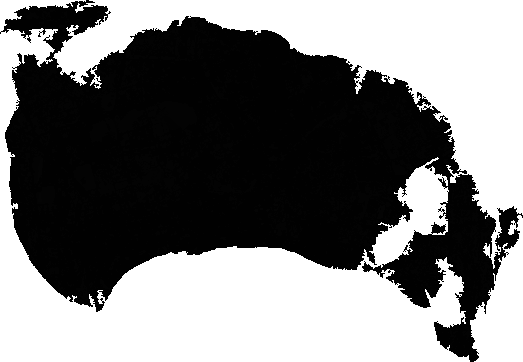

Supplement: Supplementary file 1 [file mmc1.zip › Supplementary/Landcover/Baku_1990.tif]

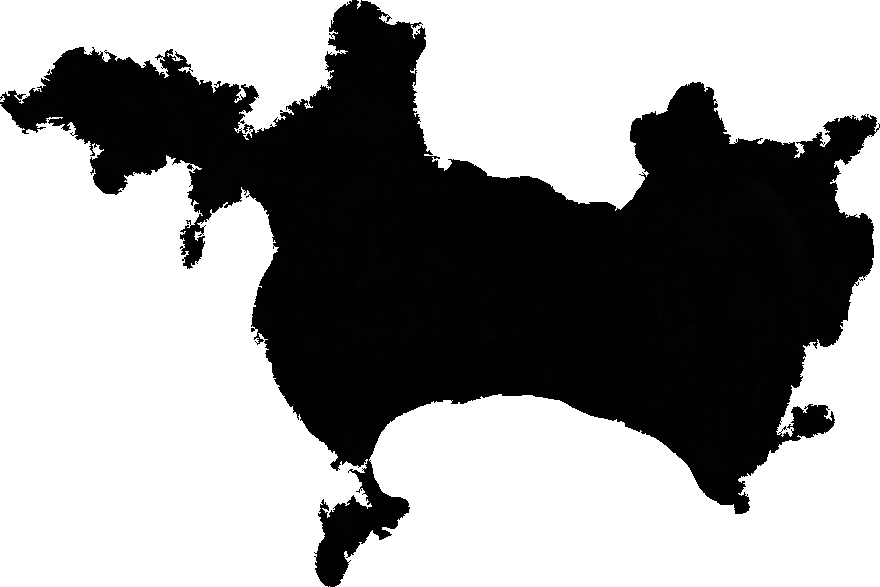

Supplement: Supplementary file 1 [file mmc1.zip › Supplementary/Landcover/Baku_2015.tif]

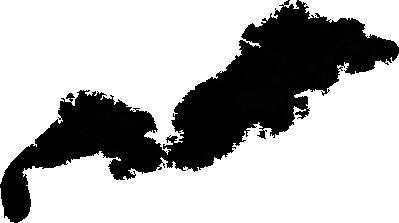

Supplement: Supplementary file 1 [file mmc1.zip › Supplementary/Landcover/Bamako_1990.tif]

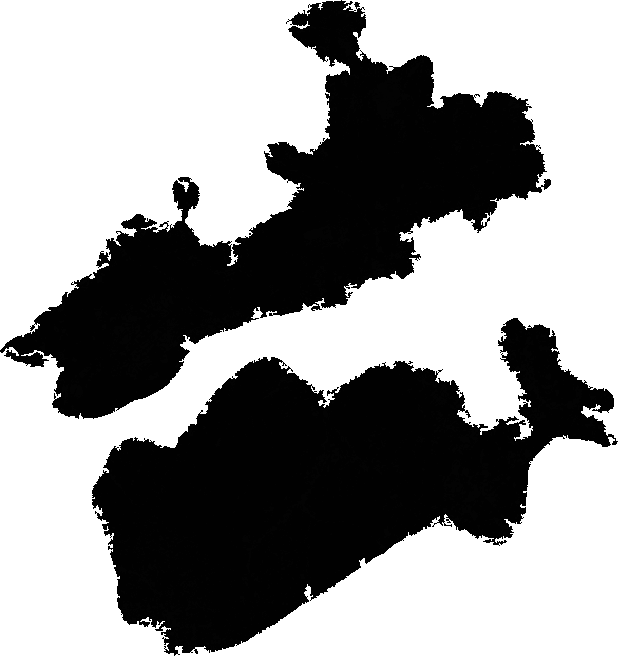

Supplement: Supplementary file 1 [file mmc1.zip › Supplementary/Landcover/Bamako_2015.tif]

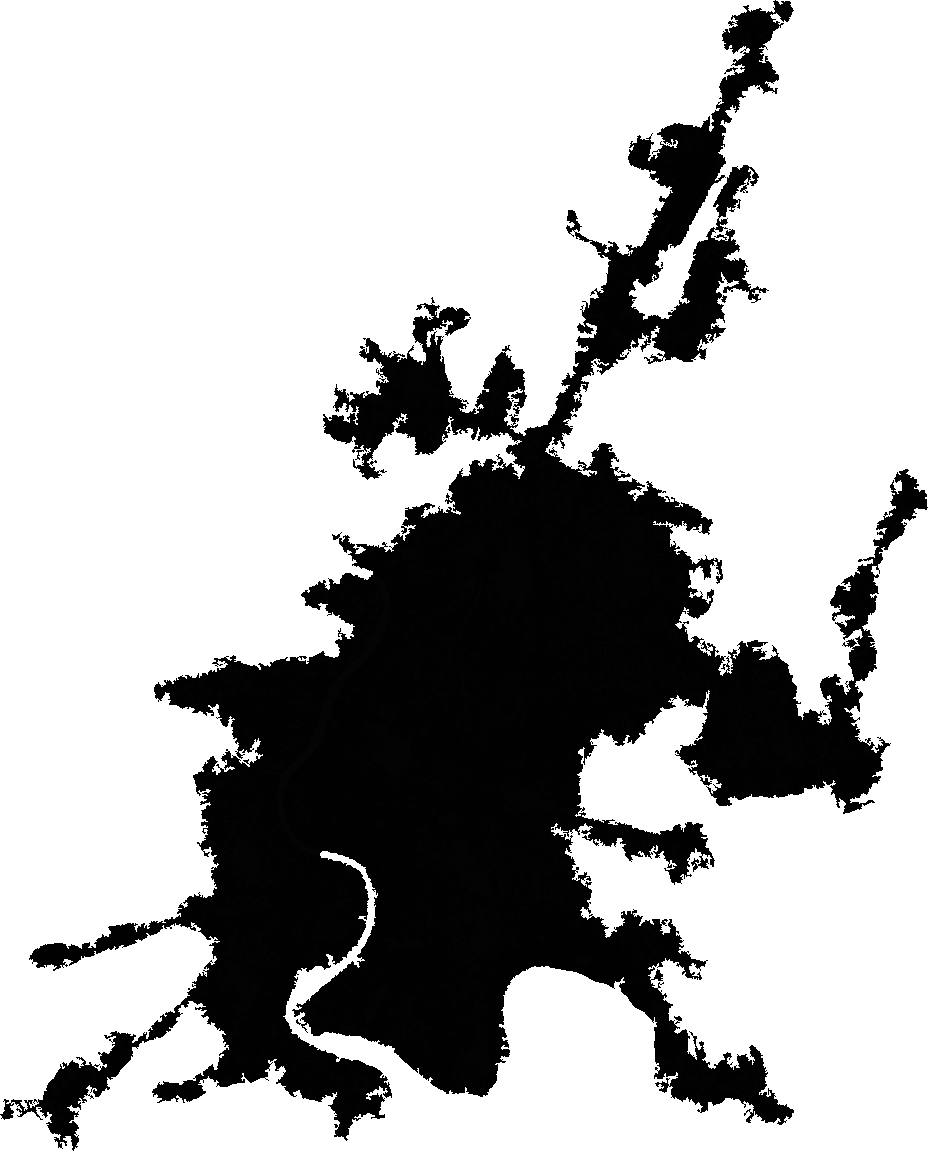

Supplement: Supplementary file 1 [file mmc1.zip › Supplementary/Landcover/Bangkok_1990.tif]

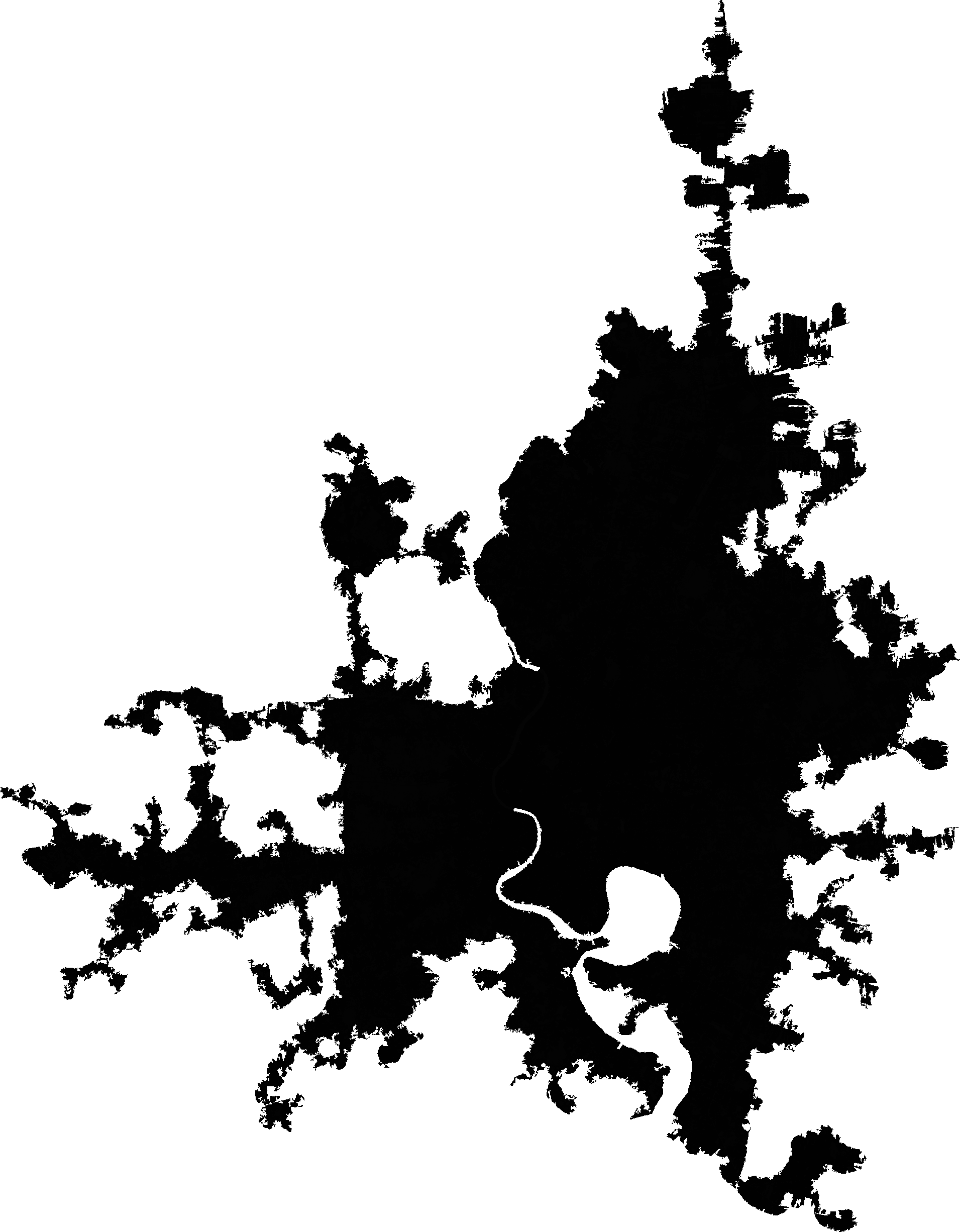

Supplement: Supplementary file 1 [file mmc1.zip › Supplementary/Landcover/Bangkok_2015.tif]

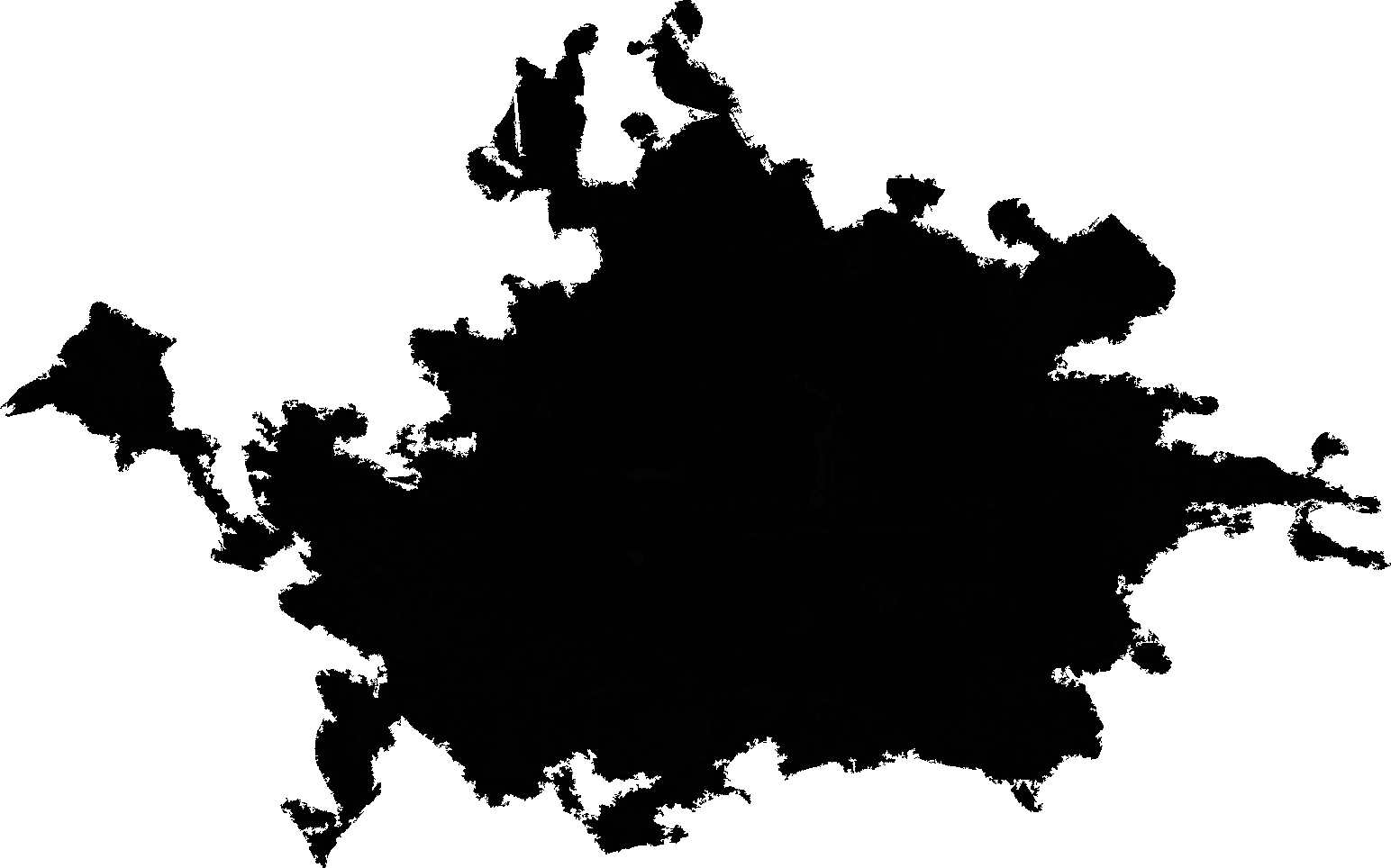

Supplement: Supplementary file 1 [file mmc1.zip › Supplementary/Landcover/Beijing_Beijing_1990.tif]

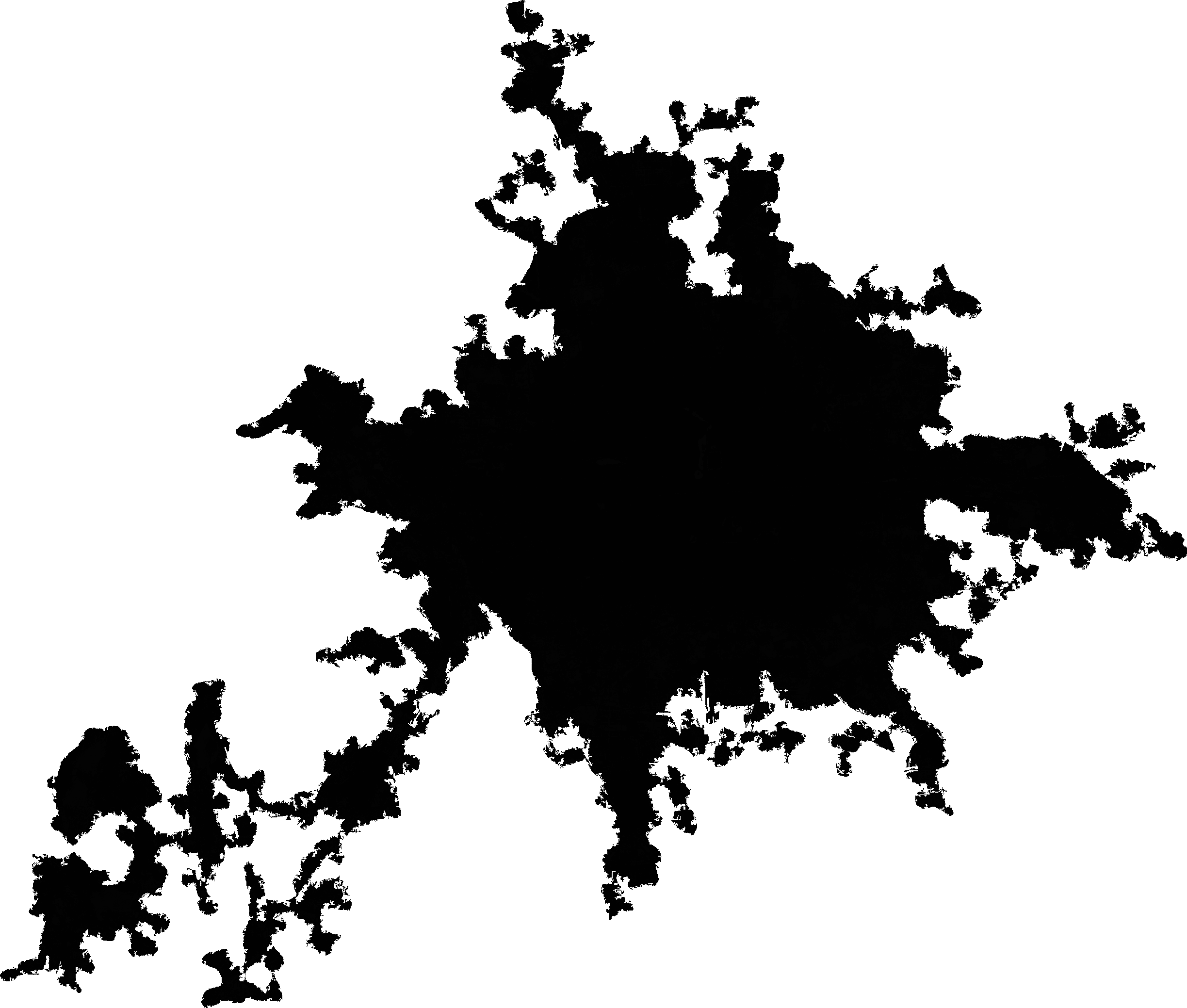

Supplement: Supplementary file 1 [file mmc1.zip › Supplementary/Landcover/Beijing_Beijing_2015.tif]

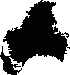

Supplement: Supplementary file 1 [file mmc1.zip › Supplementary/Landcover/Beira_1990.tif]

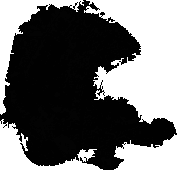

Supplement: Supplementary file 1 [file mmc1.zip › Supplementary/Landcover/Beira_2015.tif]

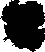

Supplement: Supplementary file 1 [file mmc1.zip › Supplementary/Landcover/Belgaum_1990.tif]

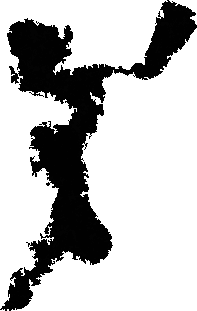

Supplement: Supplementary file 1 [file mmc1.zip › Supplementary/Landcover/Belgaum_2015.tif]

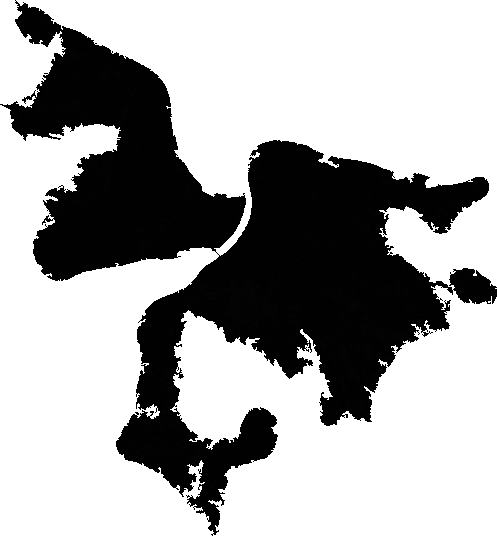

Supplement: Supplementary file 1 [file mmc1.zip › Supplementary/Landcover/Belgrade_1990.tif]

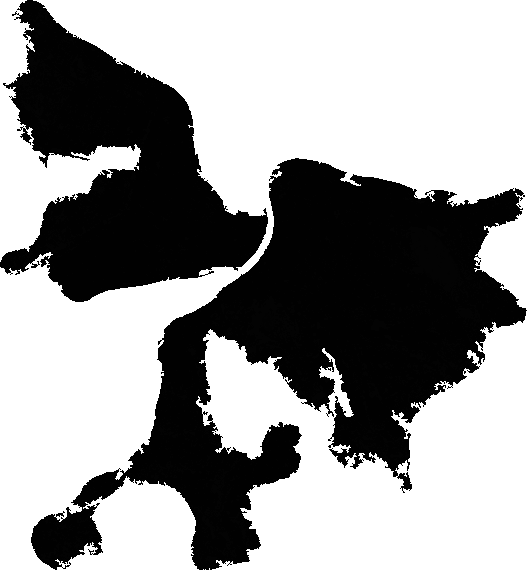

Supplement: Supplementary file 1 [file mmc1.zip › Supplementary/Landcover/Belgrade_2015.tif]

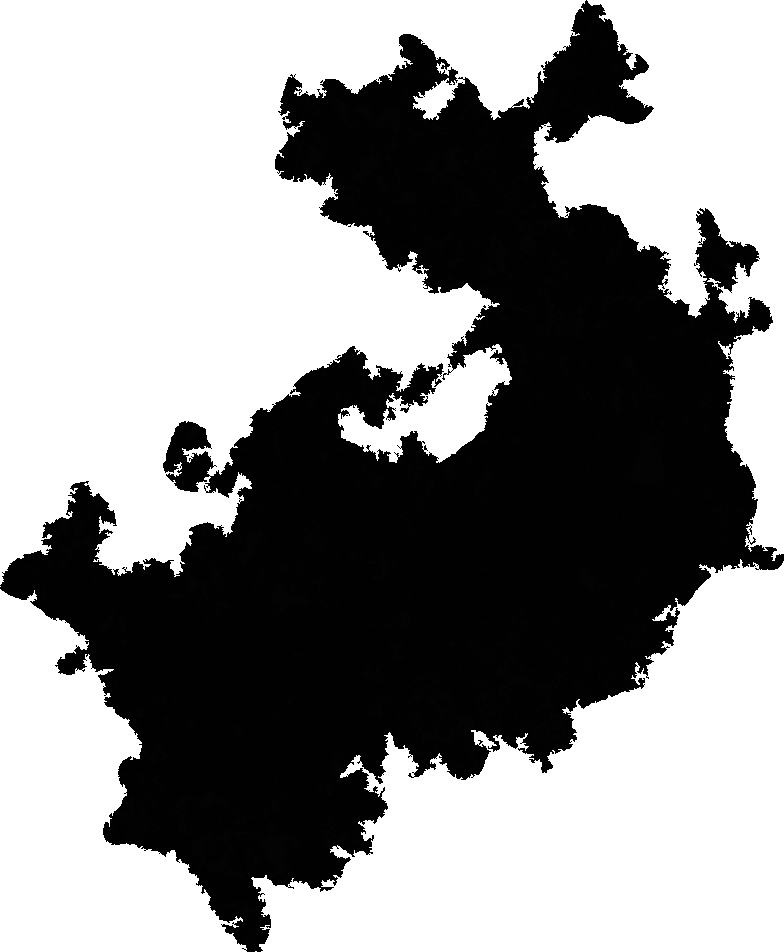

Supplement: Supplementary file 1 [file mmc1.zip › Supplementary/Landcover/Belo_Horizonte_1990.tif]

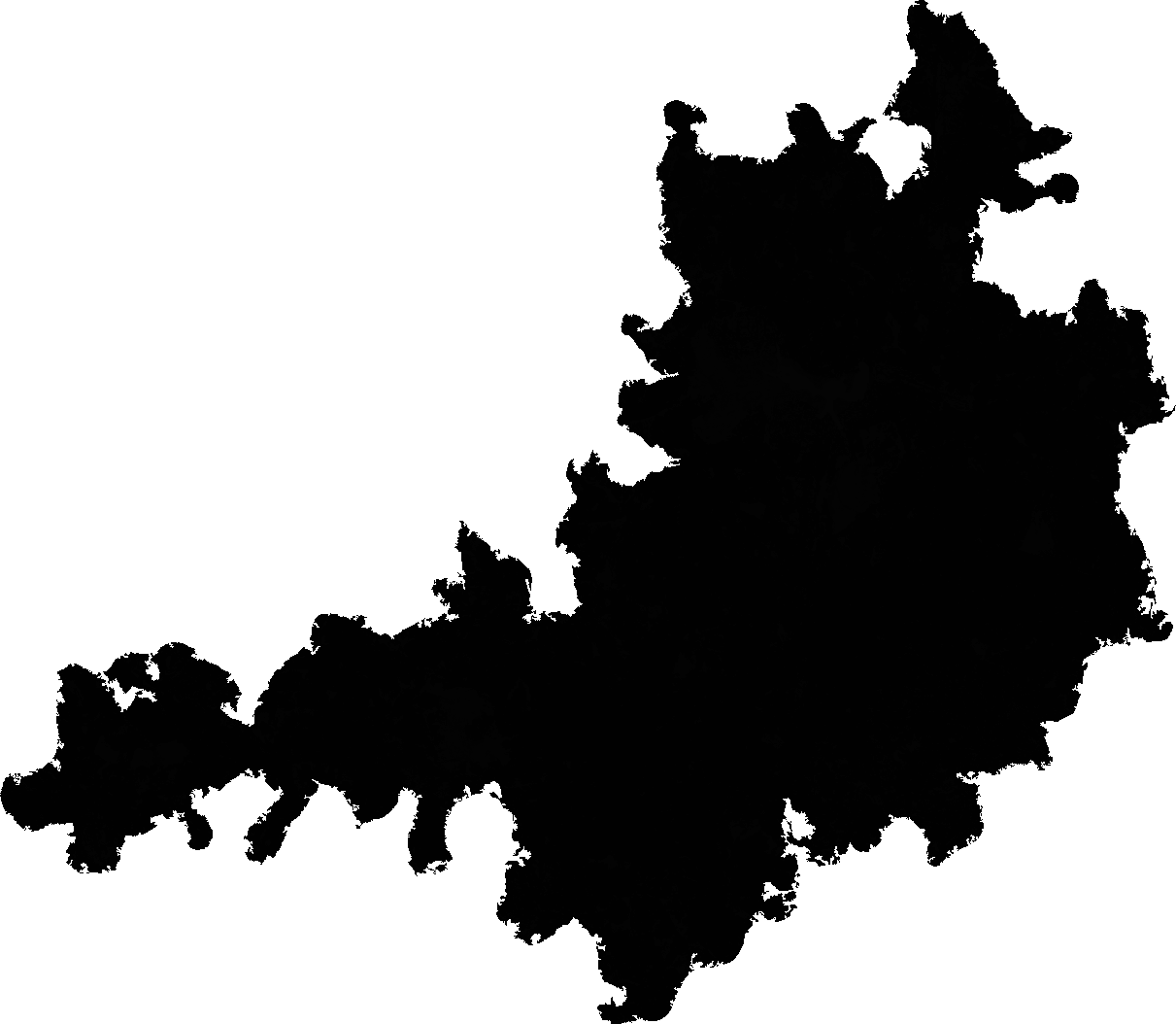

Supplement: Supplementary file 1 [file mmc1.zip › Supplementary/Landcover/Belo_Horizonte_2015.tif]

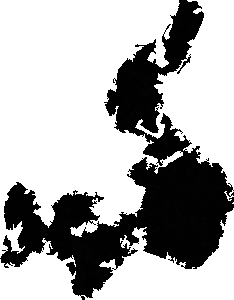

Supplement: Supplementary file 1 [file mmc1.zip › Supplementary/Landcover/Berezniki_1990.tif]

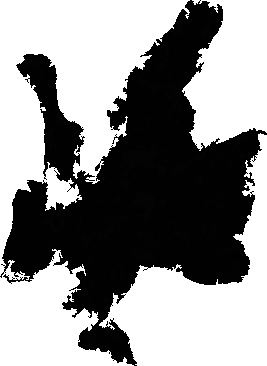

Supplement: Supplementary file 1 [file mmc1.zip › Supplementary/Landcover/Berezniki_2015.tif]

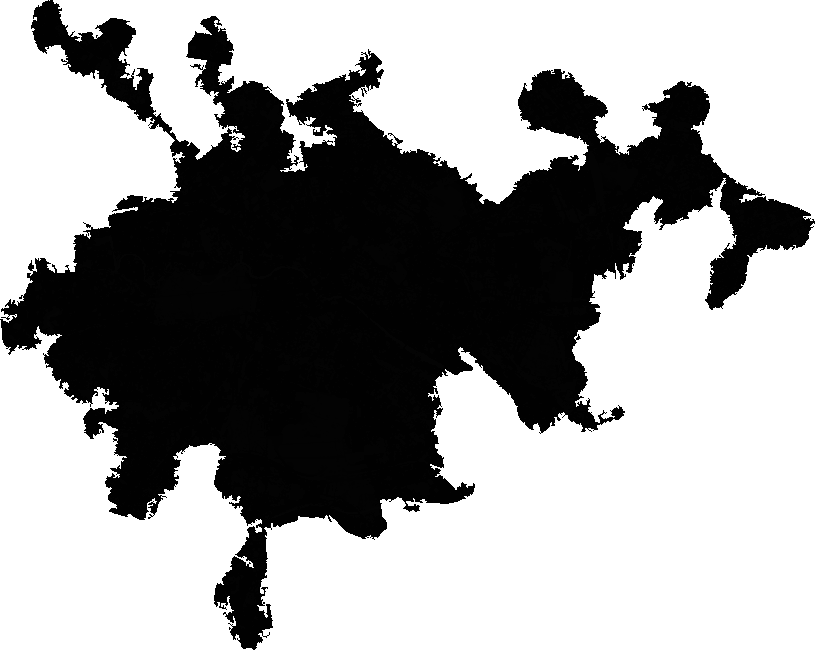

Supplement: Supplementary file 1 [file mmc1.zip › Supplementary/Landcover/Berlin_1990.tif]

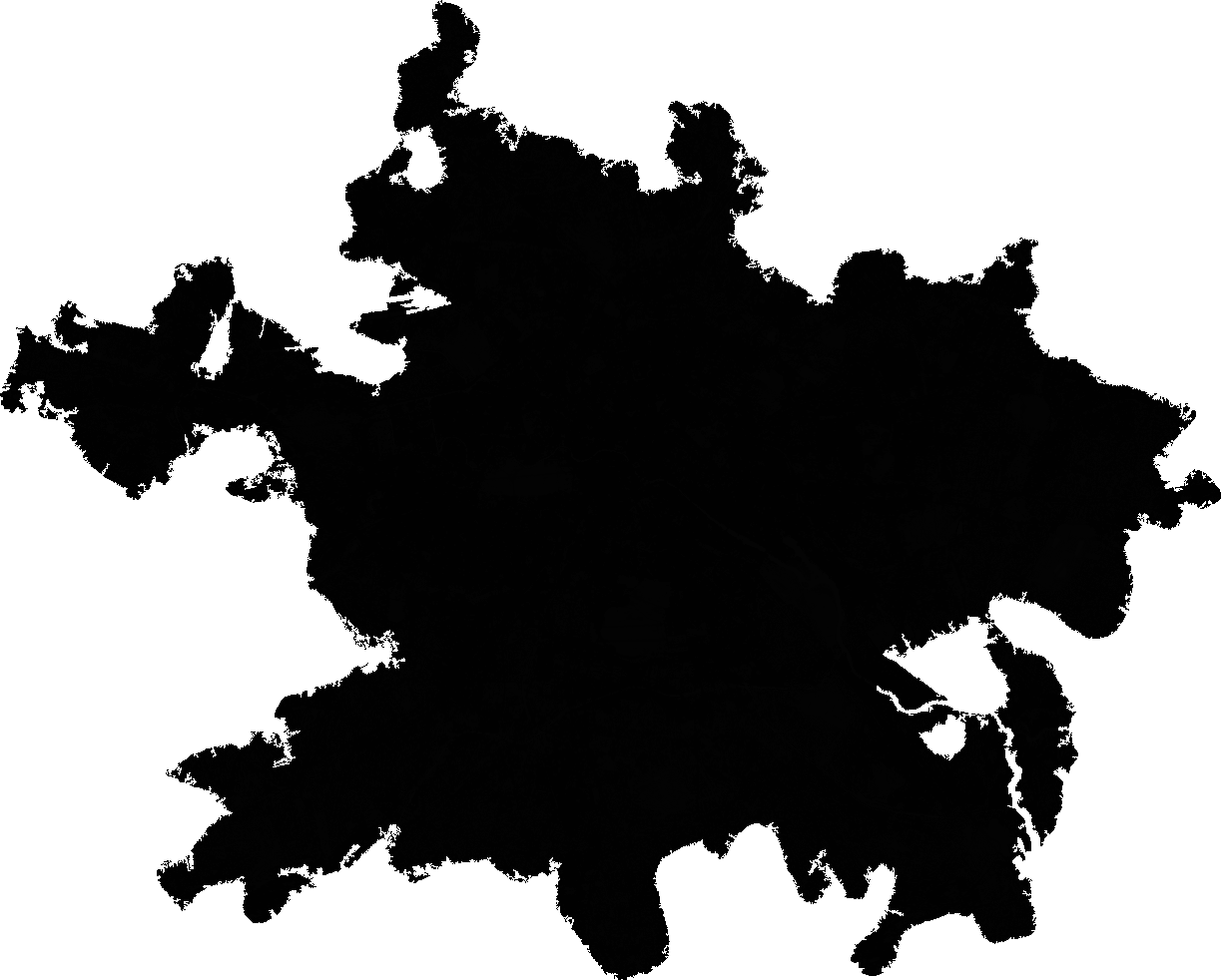

Supplement: Supplementary file 1 [file mmc1.zip › Supplementary/Landcover/Berlin_2015.tif]

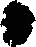

Supplement: Supplementary file 1 [file mmc1.zip › Supplementary/Landcover/Bicheng_Chongqing_1990.tif]

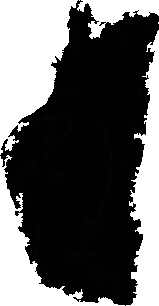

Supplement: Supplementary file 1 [file mmc1.zip › Supplementary/Landcover/Bicheng_Chongqing_2015.tif]

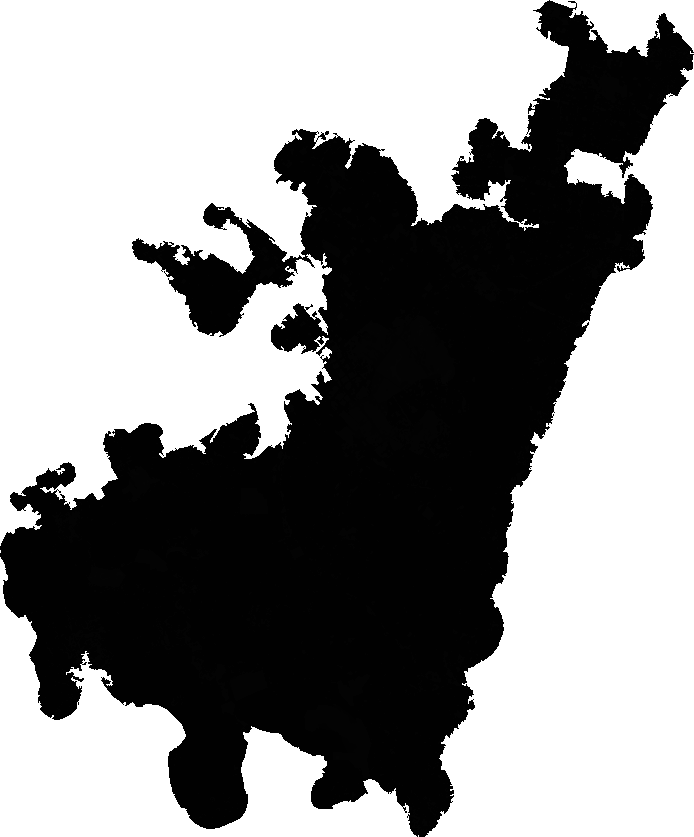

Supplement: Supplementary file 1 [file mmc1.zip › Supplementary/Landcover/Bogota_1990.tif]

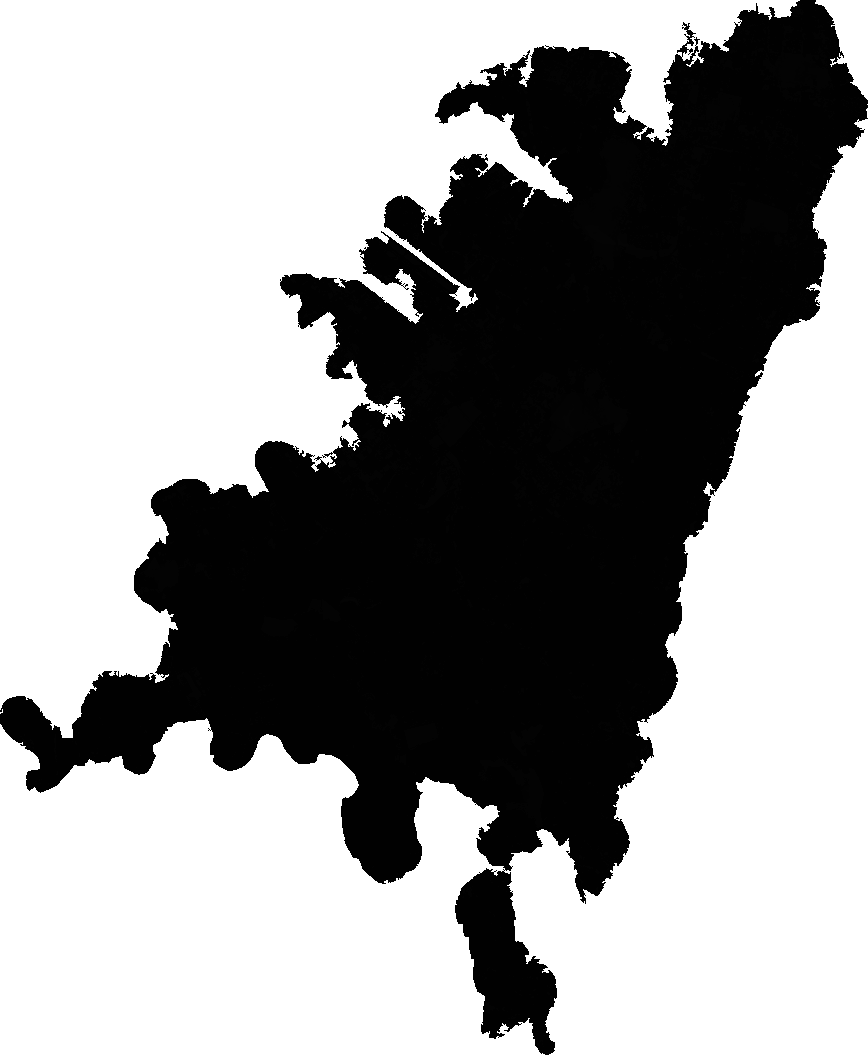

Supplement: Supplementary file 1 [file mmc1.zip › Supplementary/Landcover/Bogota_2015.tif]

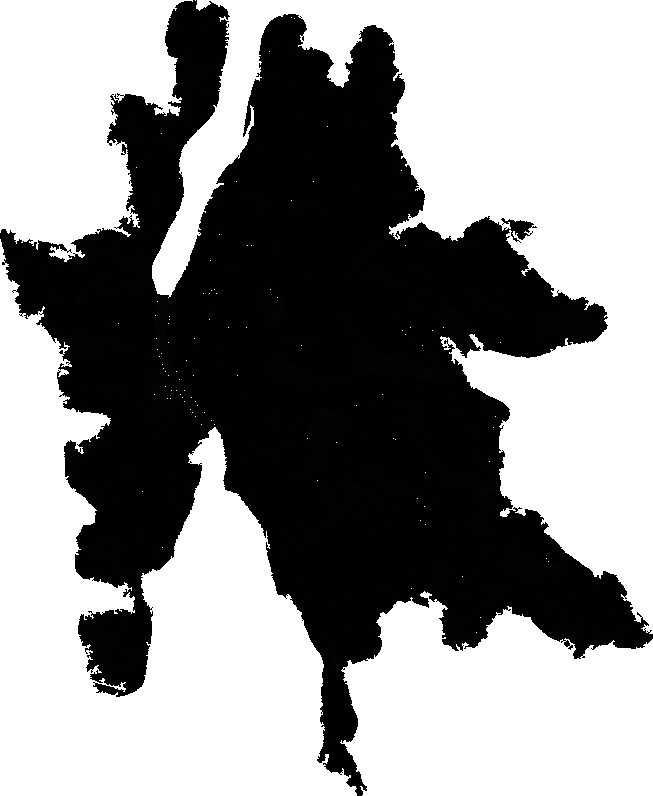

Supplement: Supplementary file 1 [file mmc1.zip › Supplementary/Landcover/Budapest_1990.tif]

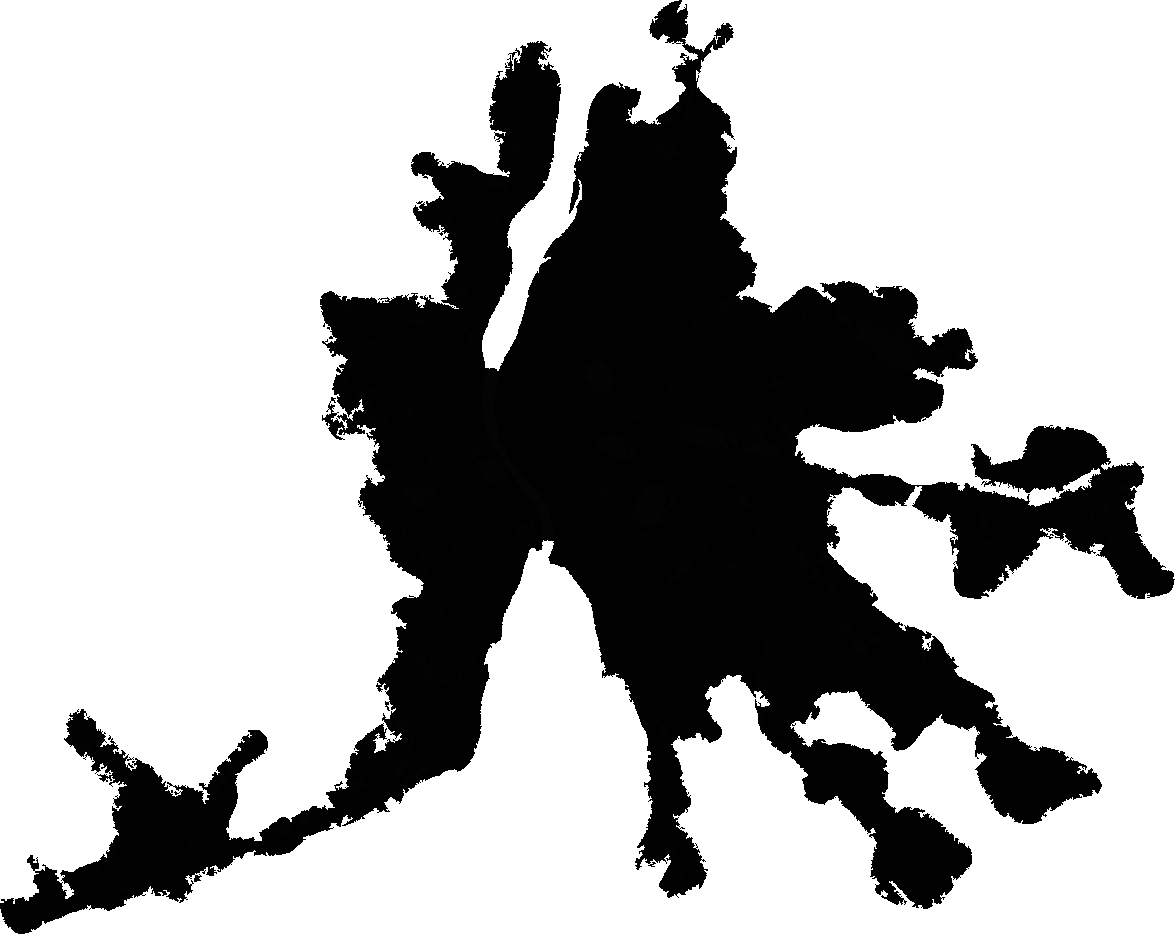

Supplement: Supplementary file 1 [file mmc1.zip › Supplementary/Landcover/Budapest_2015.tif]

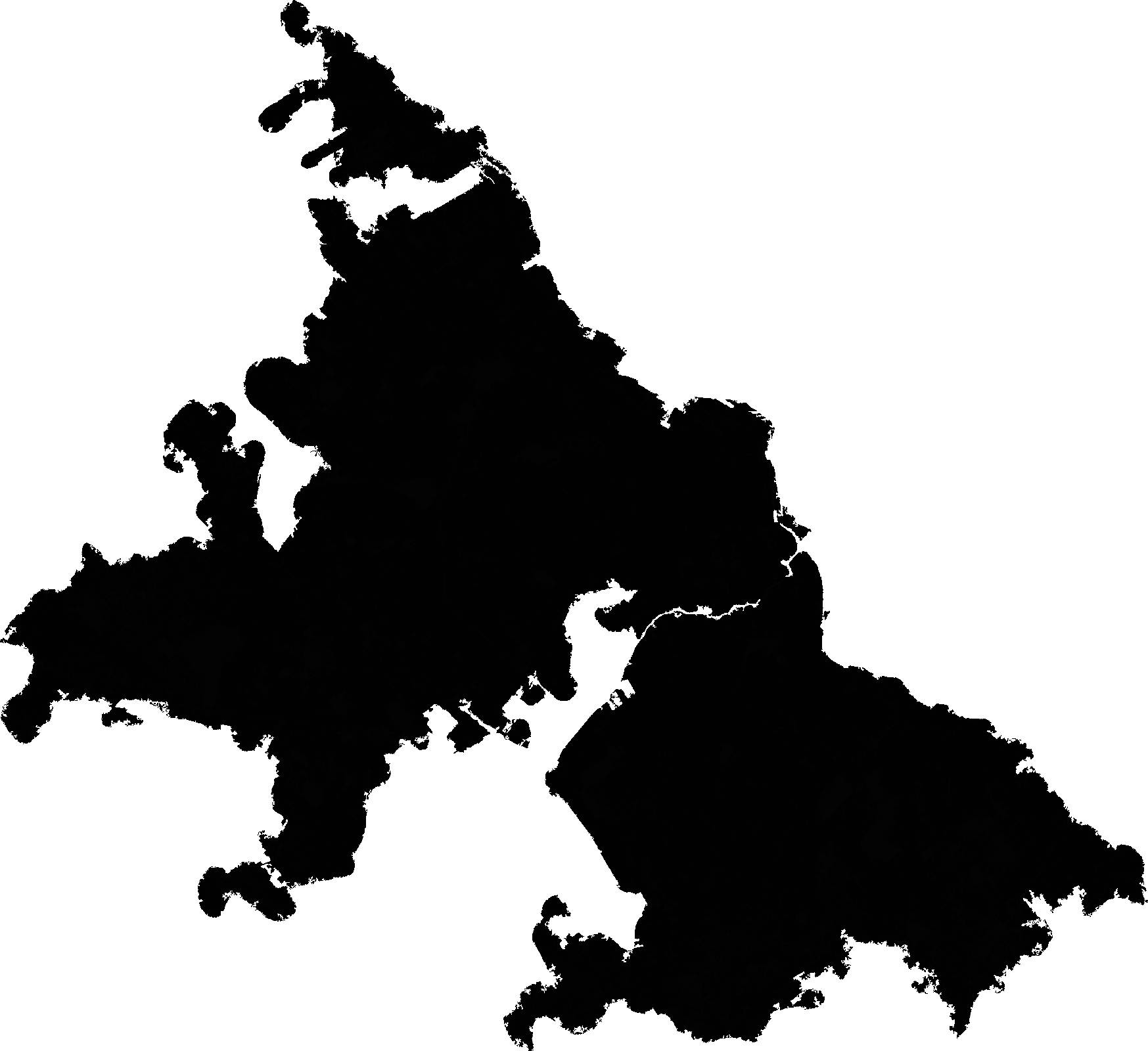

Supplement: Supplementary file 1 [file mmc1.zip › Supplementary/Landcover/Buenos_Aires_1990.tif]

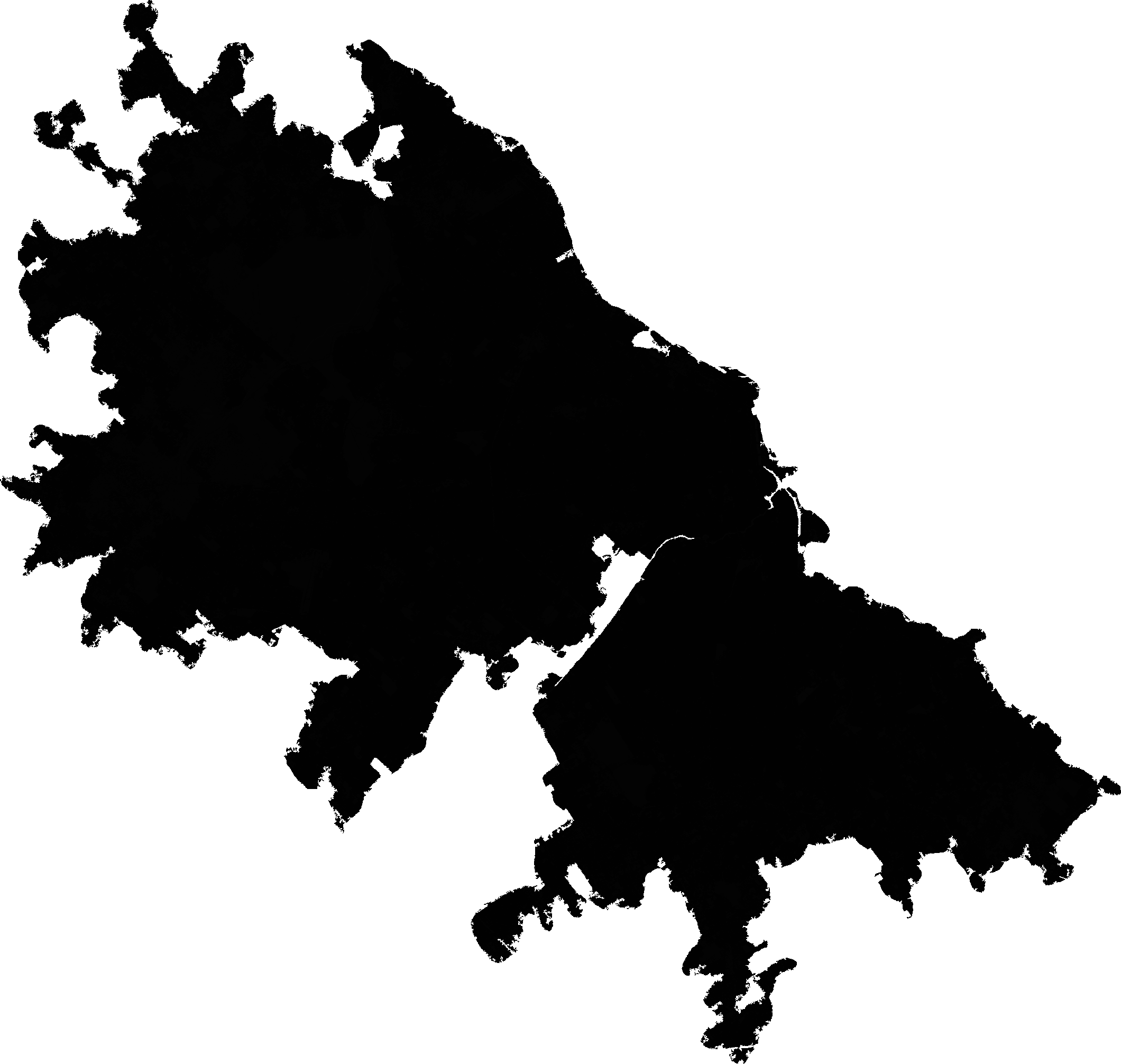

Supplement: Supplementary file 1 [file mmc1.zip › Supplementary/Landcover/Buenos_Aires_2015.tif]

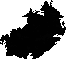

Supplement: Supplementary file 1 [file mmc1.zip › Supplementary/Landcover/Bukhara_1990.tif]

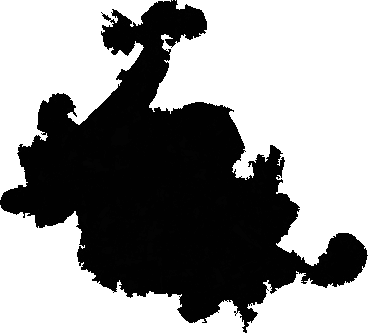

Supplement: Supplementary file 1 [file mmc1.zip › Supplementary/Landcover/Bukhara_2015.tif]

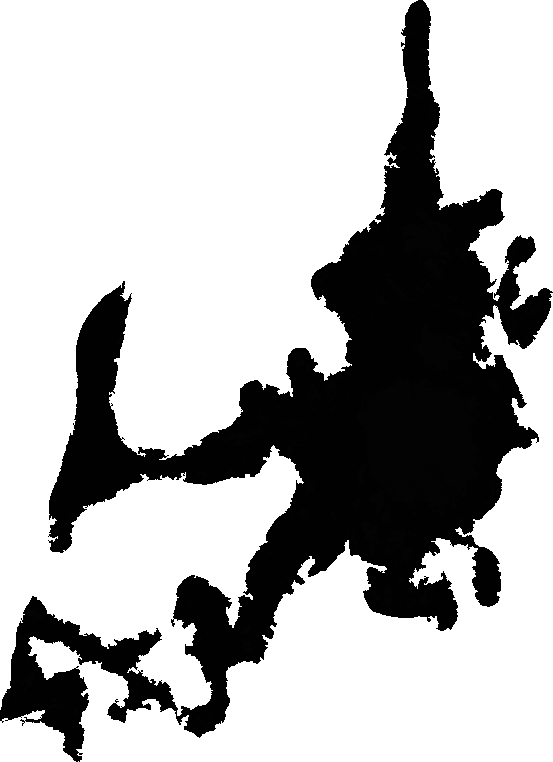

Supplement: Supplementary file 1 [file mmc1.zip › Supplementary/Landcover/Busan_1990.tif]

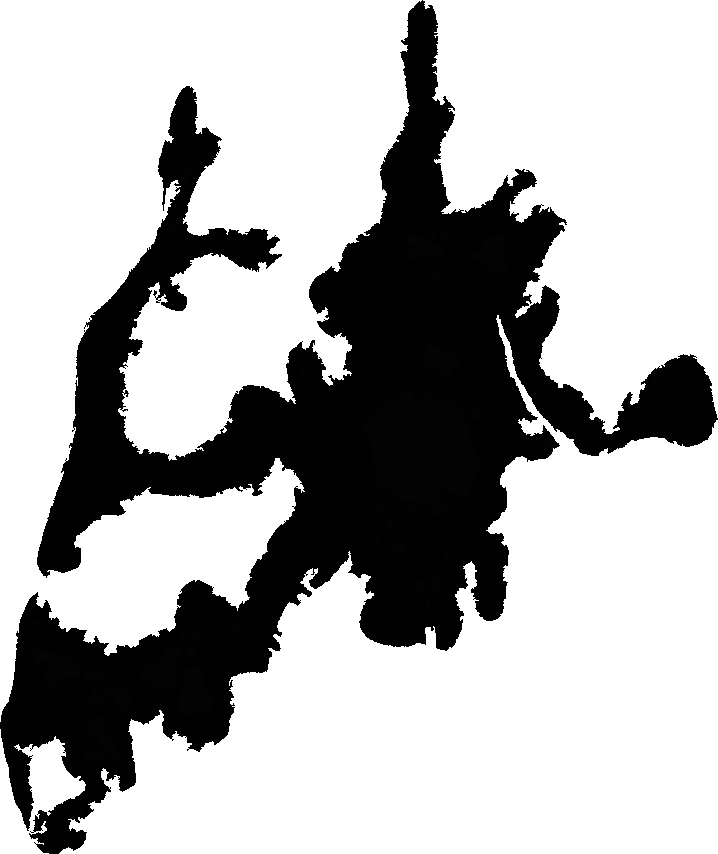

Supplement: Supplementary file 1 [file mmc1.zip › Supplementary/Landcover/Busan_2015.tif]

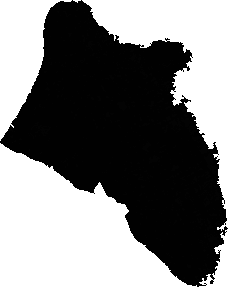

Supplement: Supplementary file 1 [file mmc1.zip › Supplementary/Landcover/Cabimas_1990.tif]

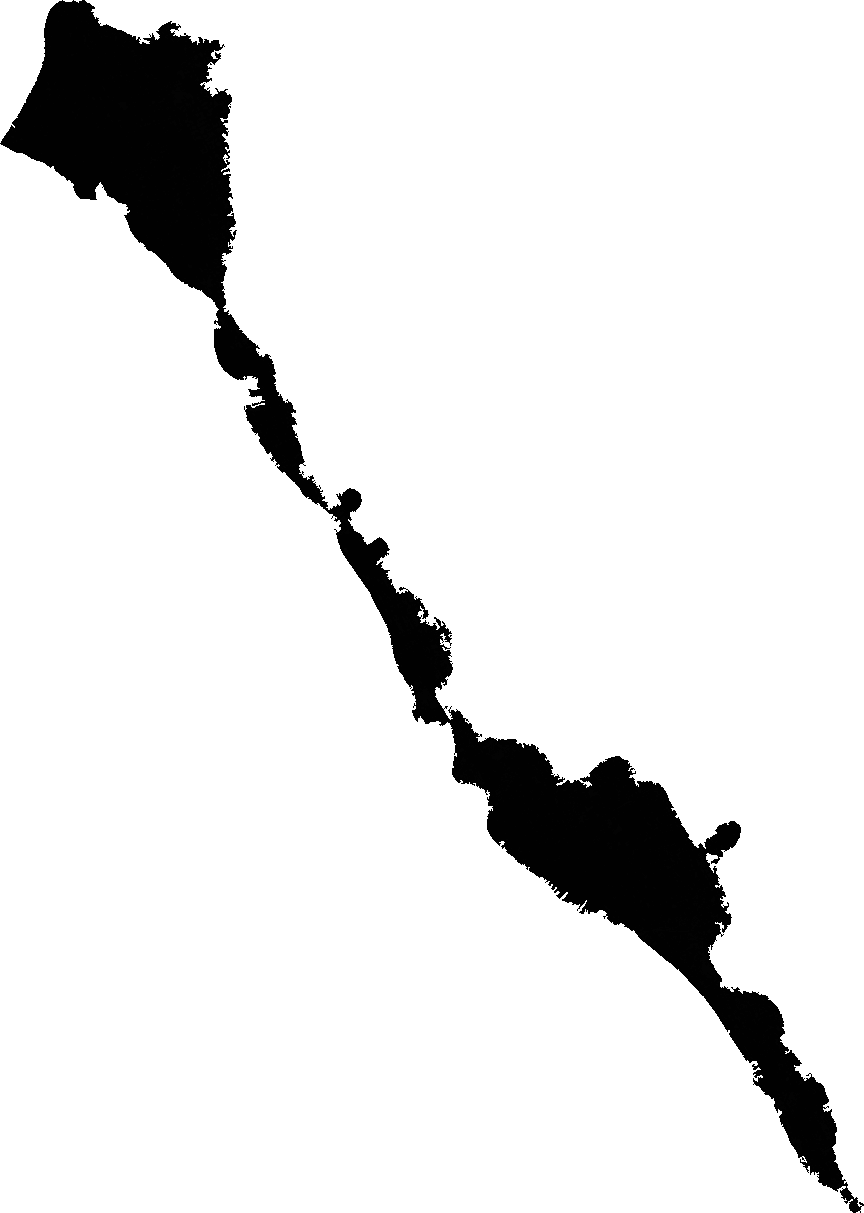

Supplement: Supplementary file 1 [file mmc1.zip › Supplementary/Landcover/Cabimas_2015.tif]

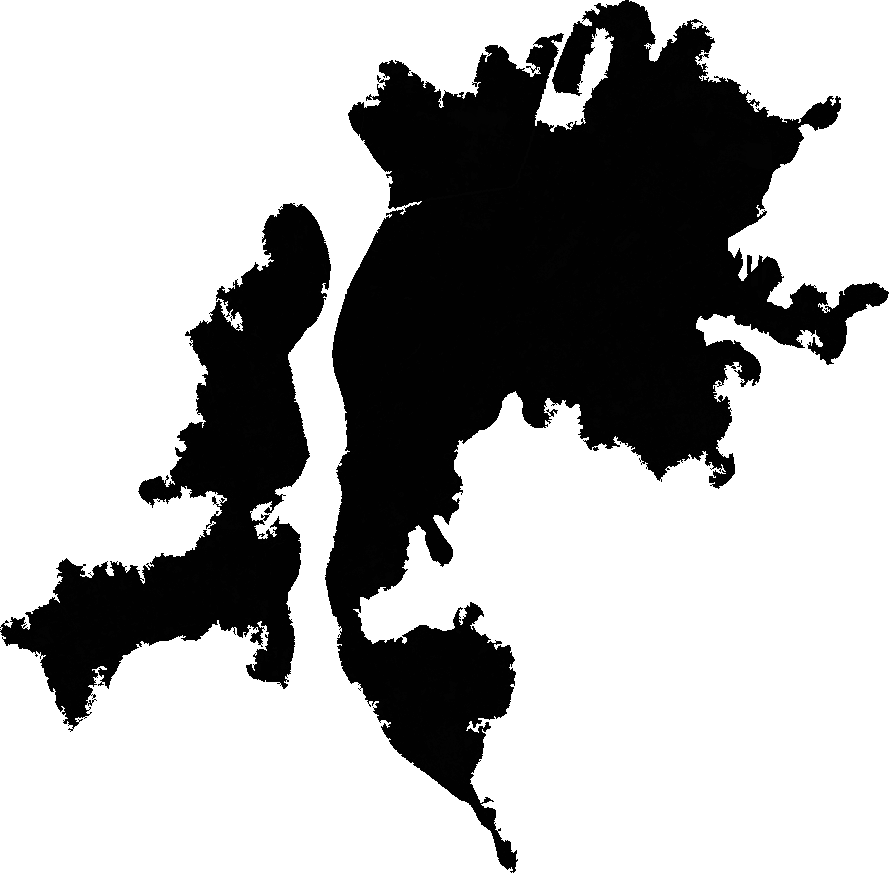

Supplement: Supplementary file 1 [file mmc1.zip › Supplementary/Landcover/Cairo_1990.tif]

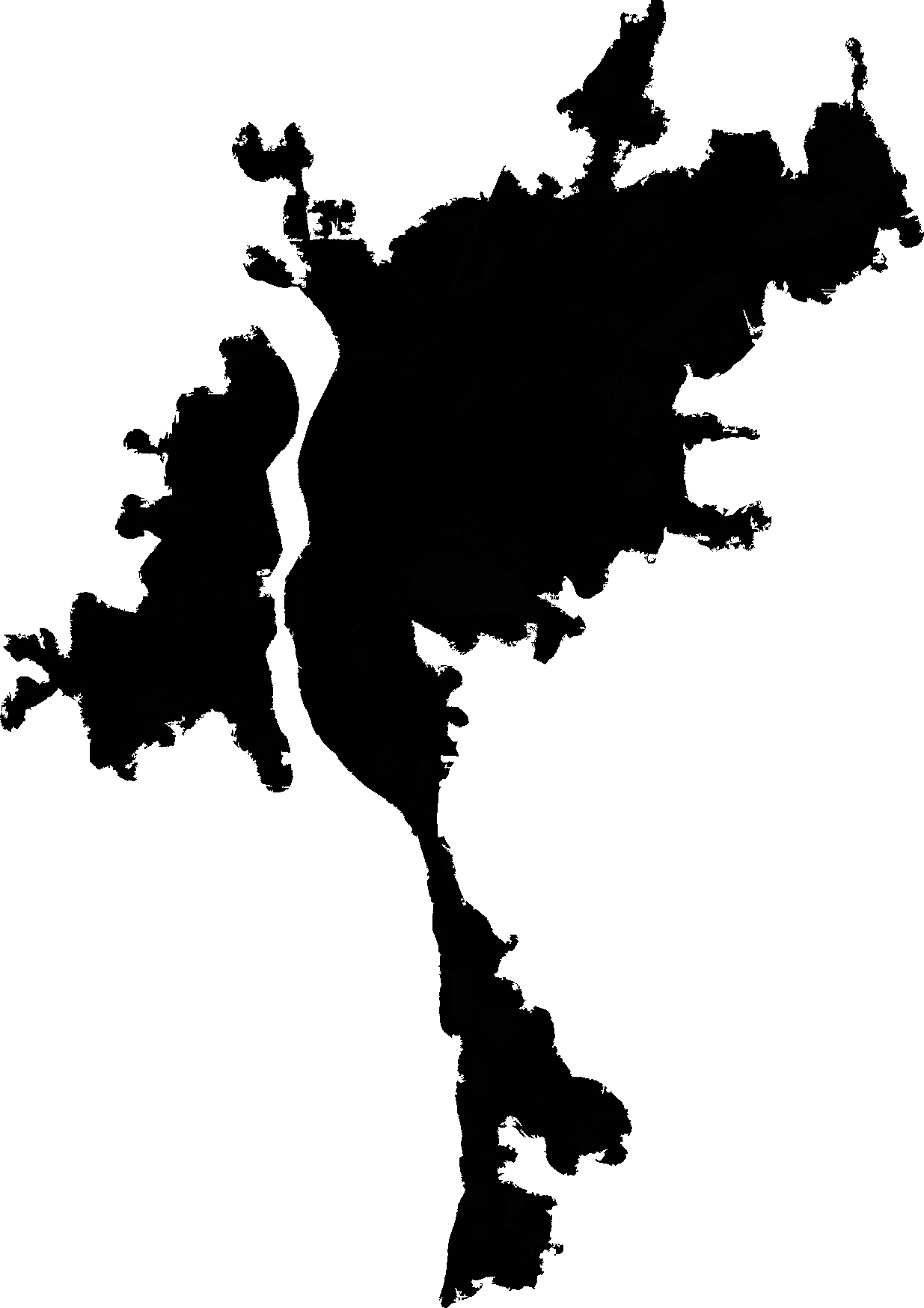

Supplement: Supplementary file 1 [file mmc1.zip › Supplementary/Landcover/Cairo_2015.tif]

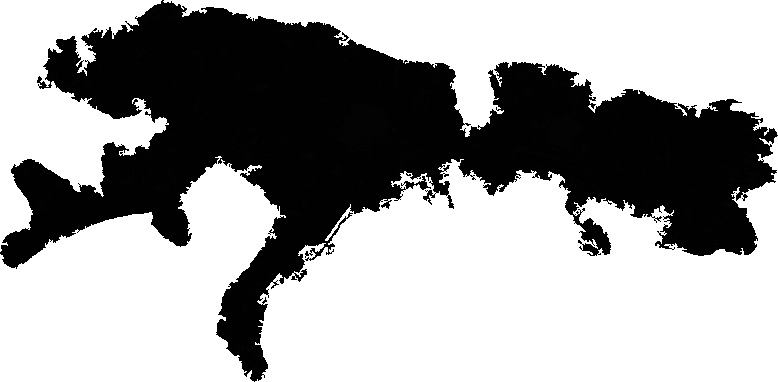

Supplement: Supplementary file 1 [file mmc1.zip › Supplementary/Landcover/Caracas_1990.tif]

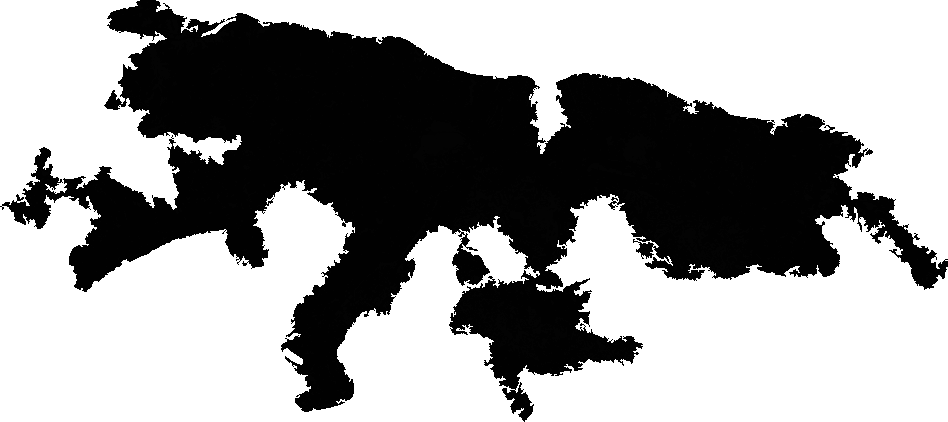

Supplement: Supplementary file 1 [file mmc1.zip › Supplementary/Landcover/Caracas_2015.tif]

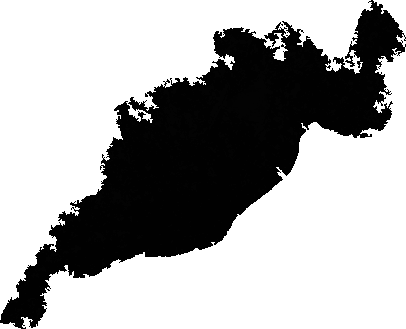

Supplement: Supplementary file 1 [file mmc1.zip › Supplementary/Landcover/Cebu_City_1990.tif]

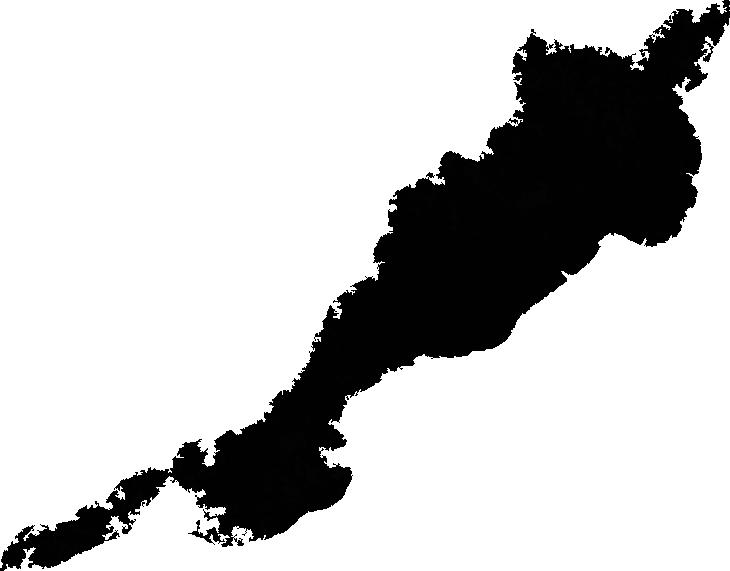

Supplement: Supplementary file 1 [file mmc1.zip › Supplementary/Landcover/Cebu_City_2015.tif]

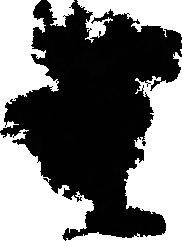

Supplement: Supplementary file 1 [file mmc1.zip › Supplementary/Landcover/Changzhi_Hunan_1990.tif]

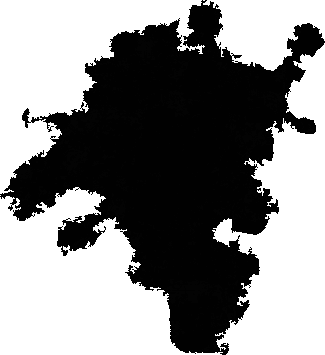

Supplement: Supplementary file 1 [file mmc1.zip › Supplementary/Landcover/Changzhi_Hunan_2015.tif]

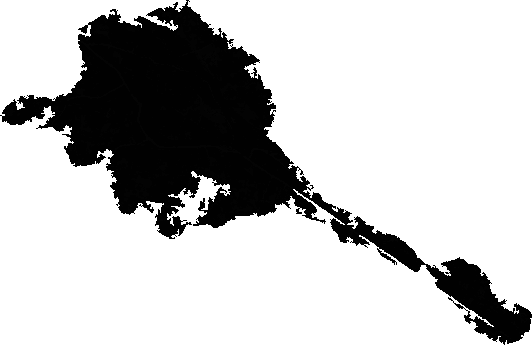

Supplement: Supplementary file 1 [file mmc1.zip › Supplementary/Landcover/Changzhou_Jingsu_1990.tif]

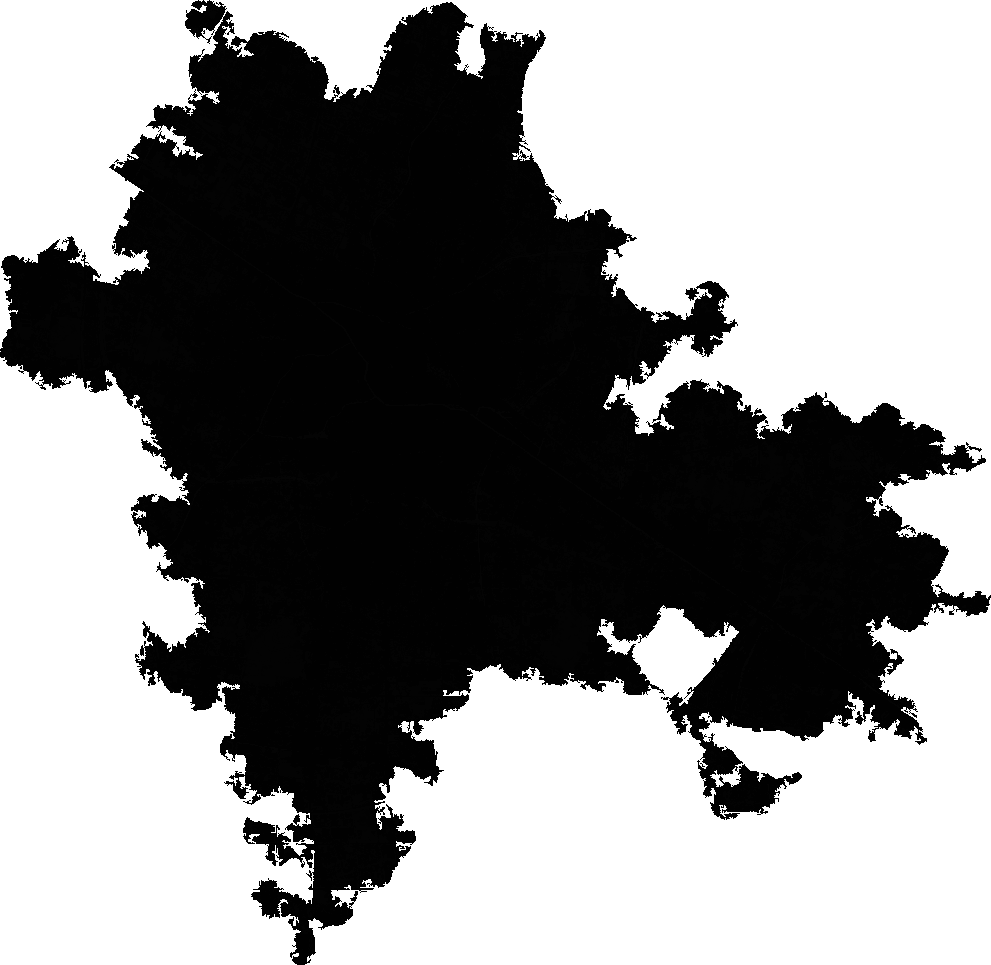

Supplement: Supplementary file 1 [file mmc1.zip › Supplementary/Landcover/Changzhou_Jingsu_2015.tif]

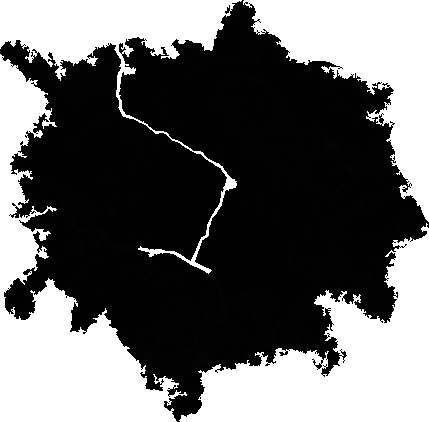

Supplement: Supplementary file 1 [file mmc1.zip › Supplementary/Landcover/Chengdu_Sichuan_1990.tif]

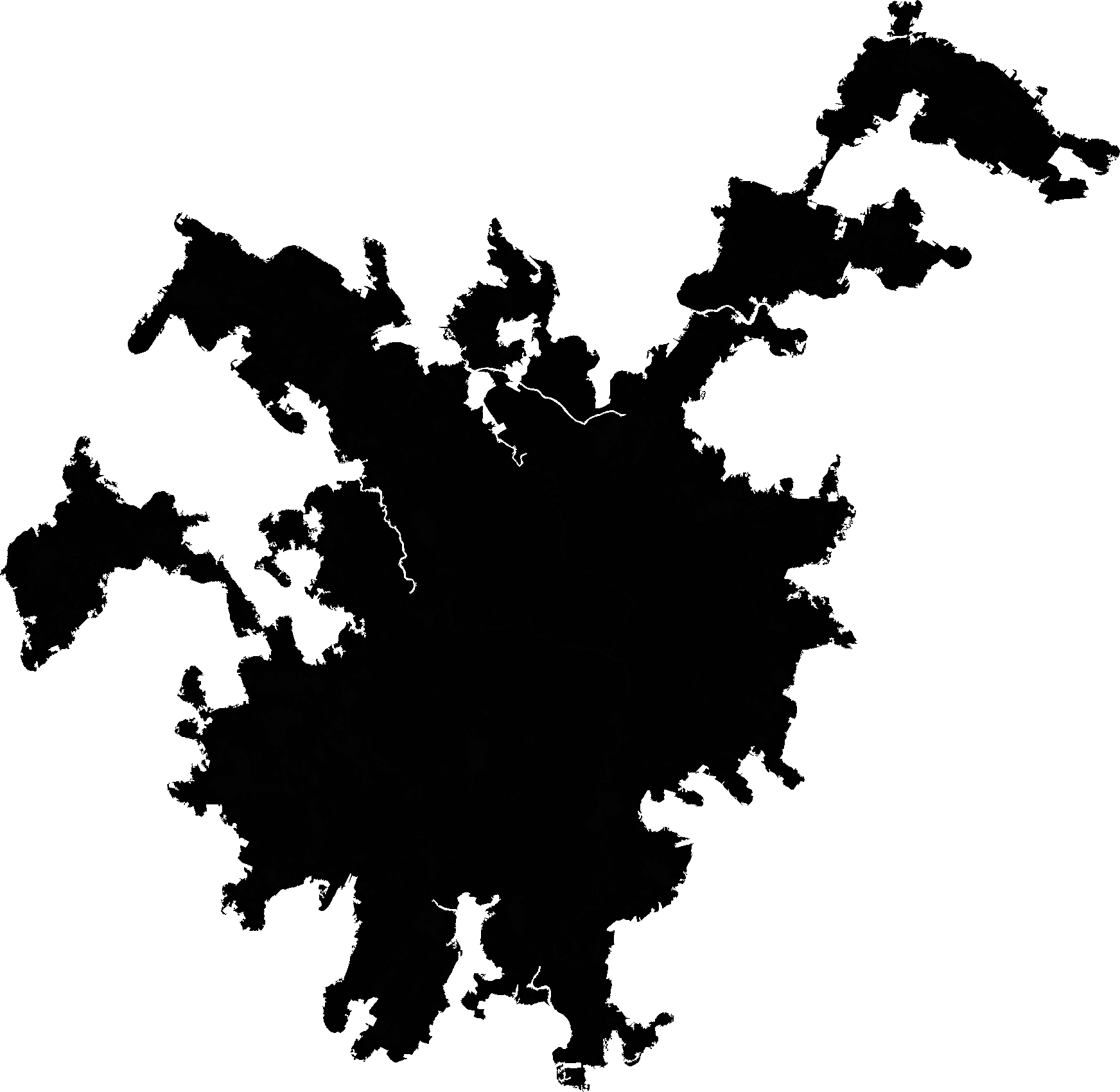

Supplement: Supplementary file 1 [file mmc1.zip › Supplementary/Landcover/Chengdu_Sichuan_2015.tif]

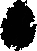

Supplement: Supplementary file 1 [file mmc1.zip › Supplementary/Landcover/Chengguan_Guizhou_1990.tif]

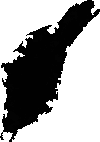

Supplement: Supplementary file 1 [file mmc1.zip › Supplementary/Landcover/Chengguan_Guizhou_2015.tif]

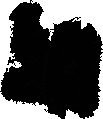

Supplement: Supplementary file 1 [file mmc1.zip › Supplementary/Landcover/Cheonan_1990.tif]

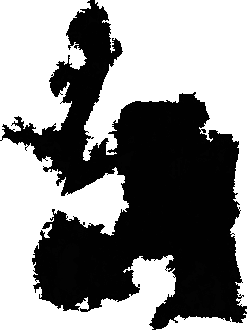

Supplement: Supplementary file 1 [file mmc1.zip › Supplementary/Landcover/Cheonan_2015.tif]

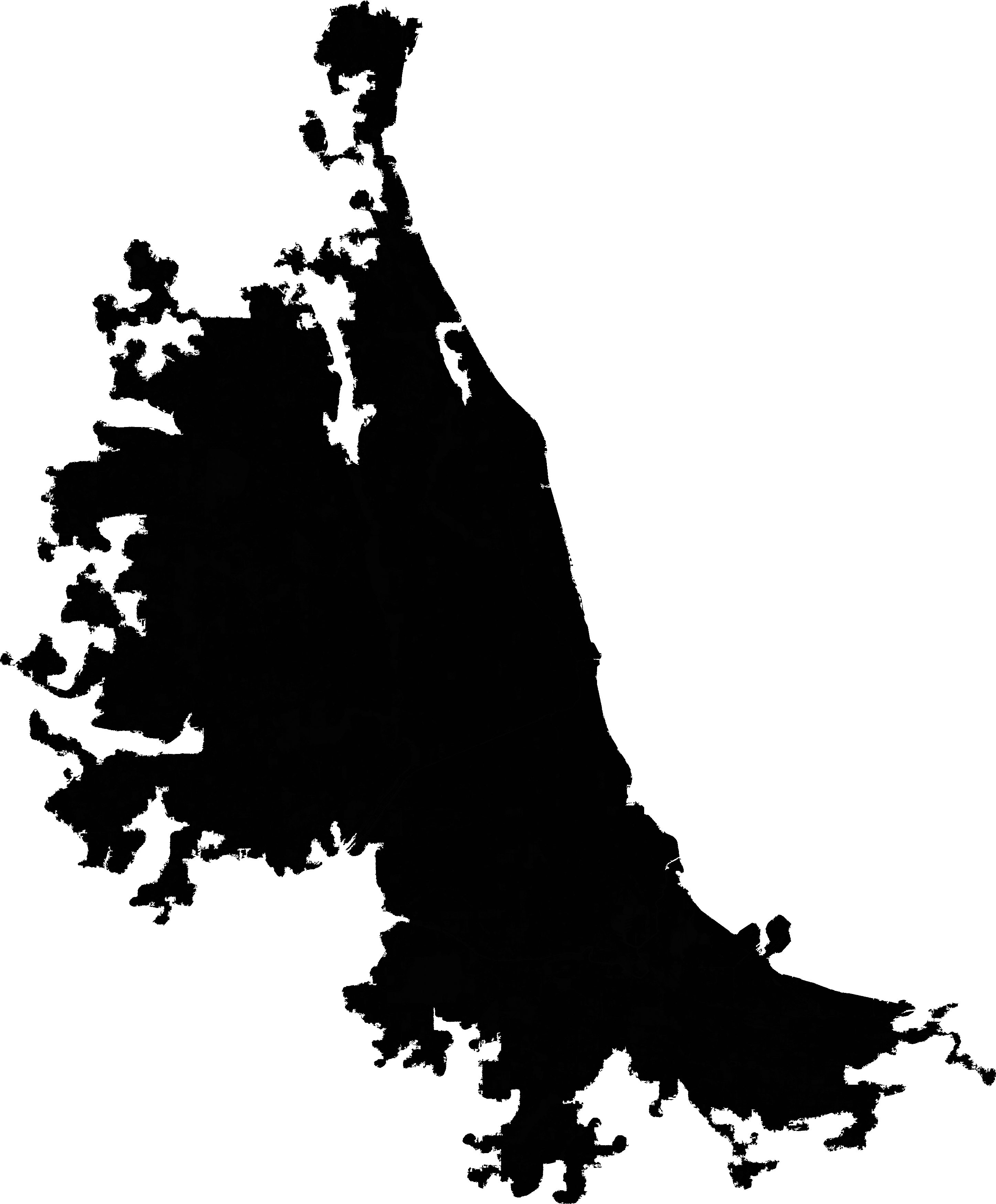

Supplement: Supplementary file 1 [file mmc1.zip › Supplementary/Landcover/Chicago_1990.tif]

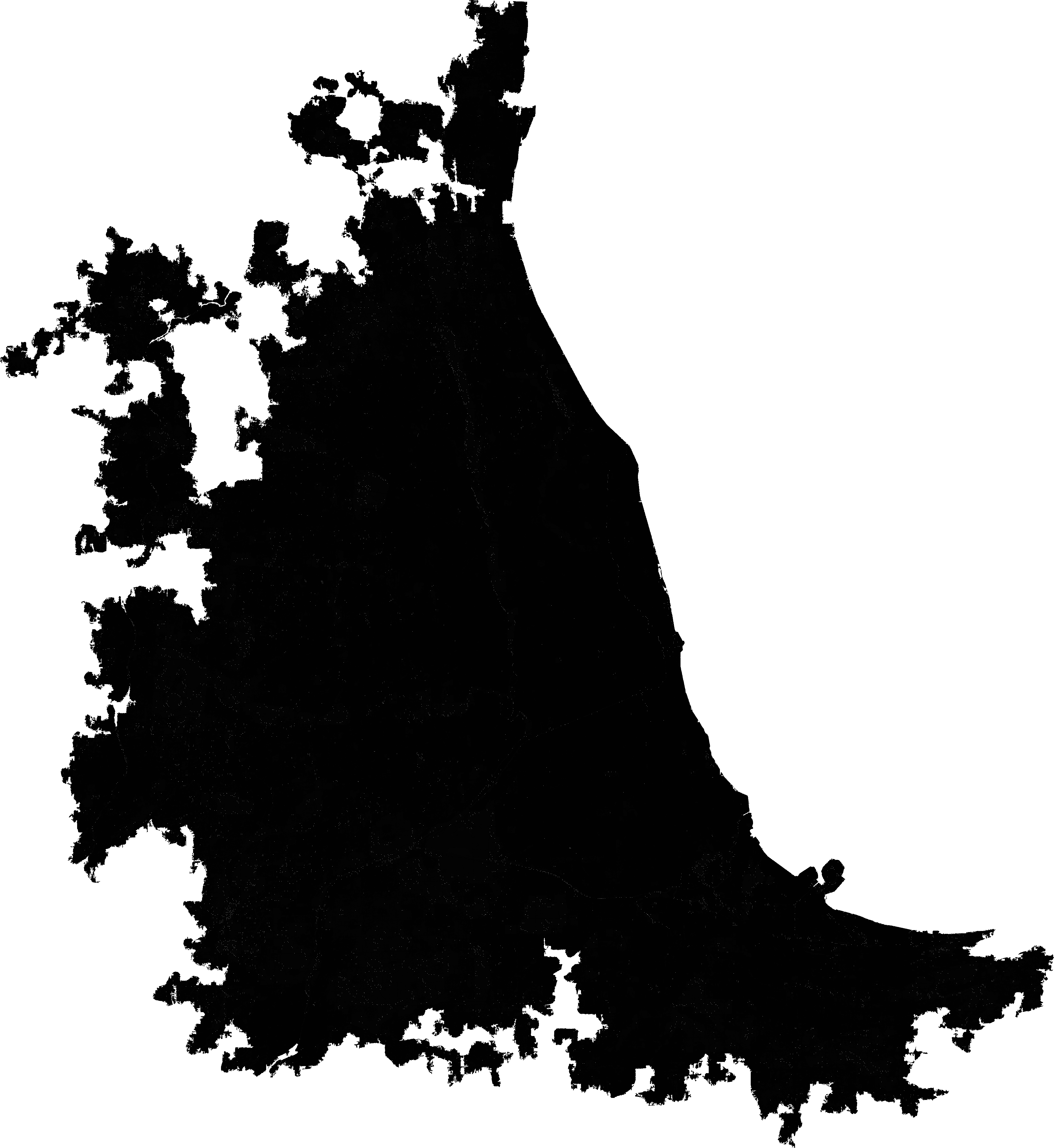

Supplement: Supplementary file 1 [file mmc1.zip › Supplementary/Landcover/Chicago_2015.tif]

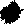

Supplement: Supplementary file 1 [file mmc1.zip › Supplementary/Landcover/Cirebon_1990.tif]

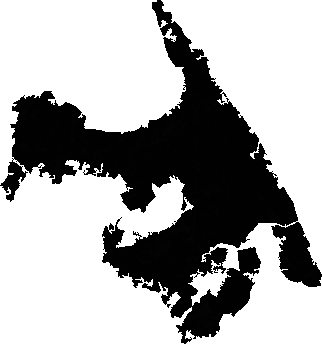

Supplement: Supplementary file 1 [file mmc1.zip › Supplementary/Landcover/Cirebon_2015.tif]

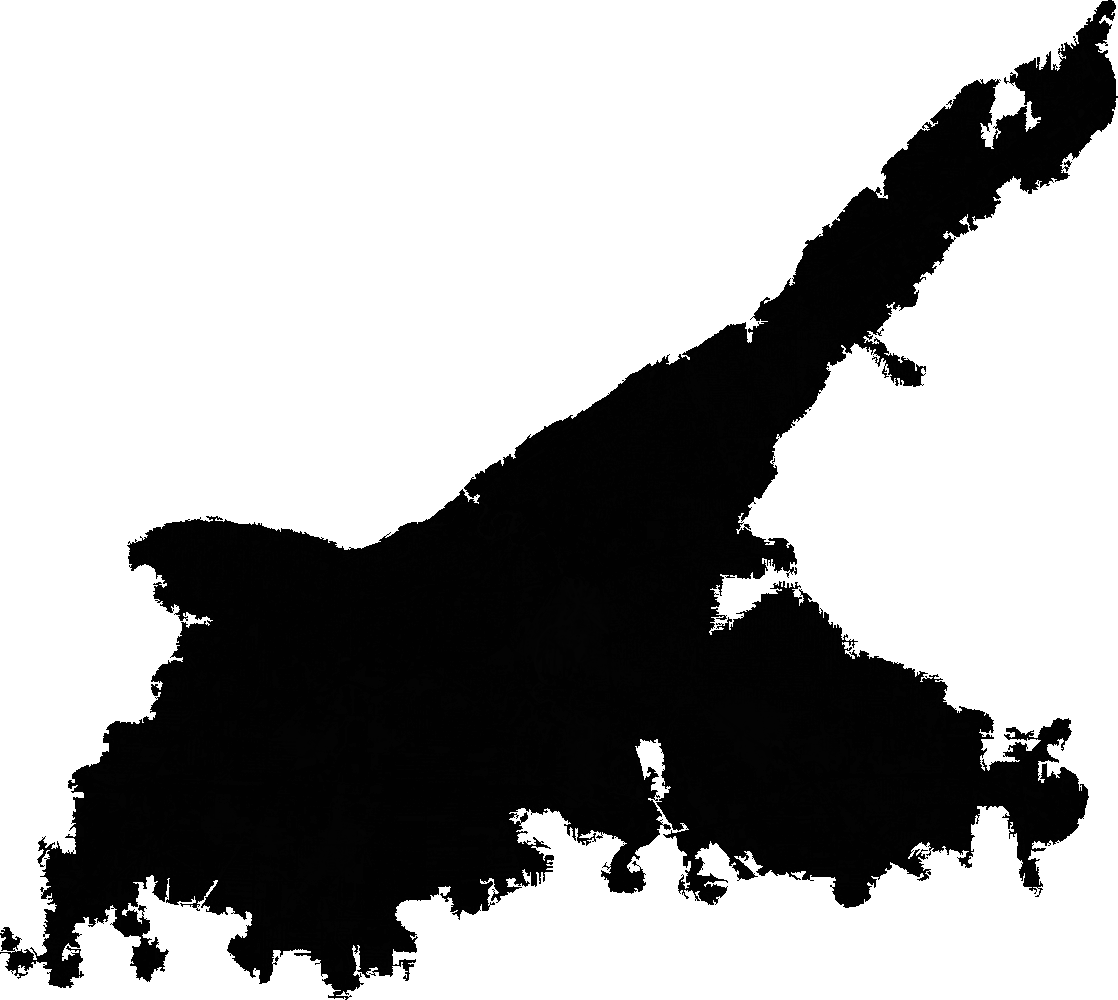

Supplement: Supplementary file 1 [file mmc1.zip › Supplementary/Landcover/Cleveland_1990.tif]

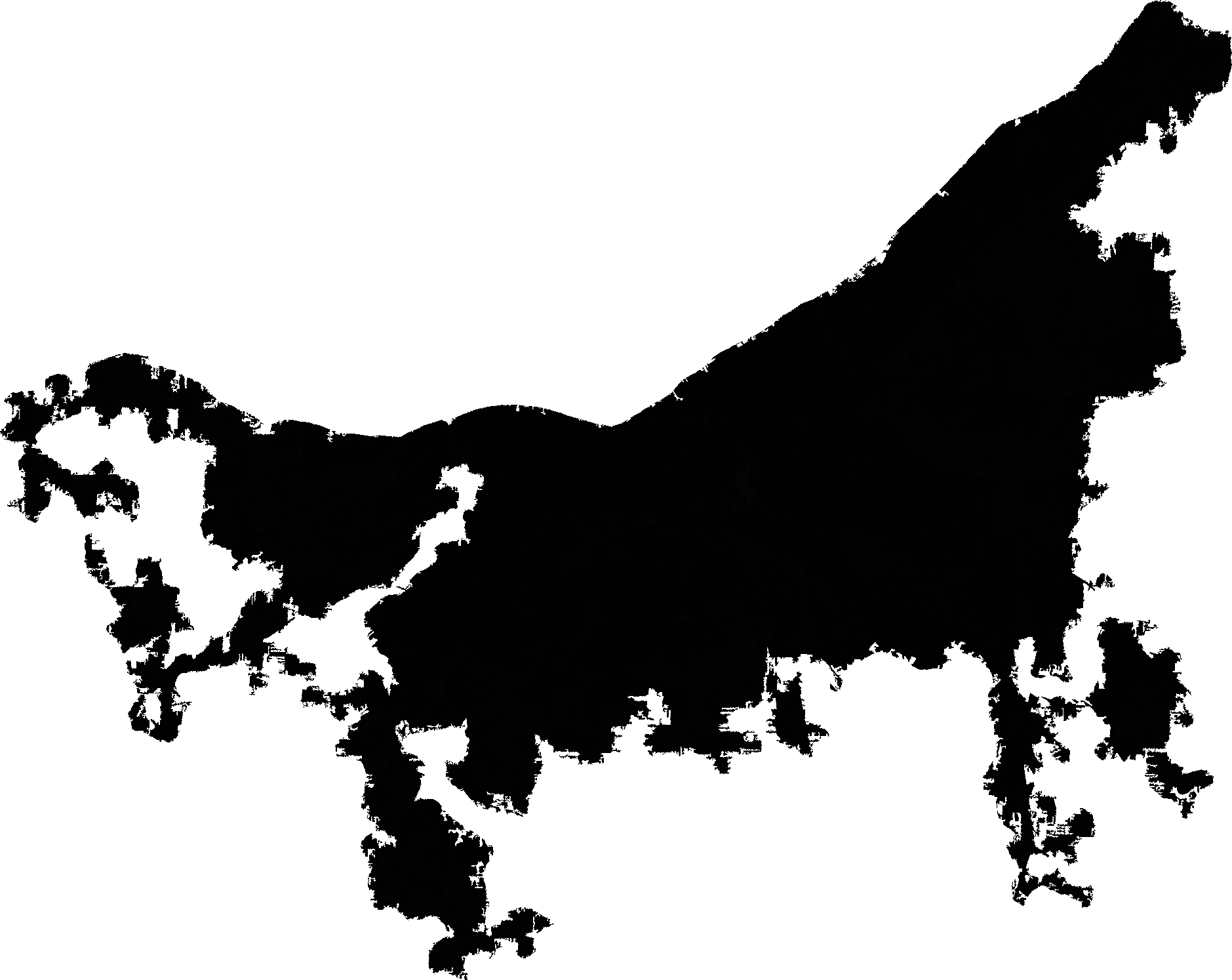

Supplement: Supplementary file 1 [file mmc1.zip › Supplementary/Landcover/Cleveland_2015.tif]

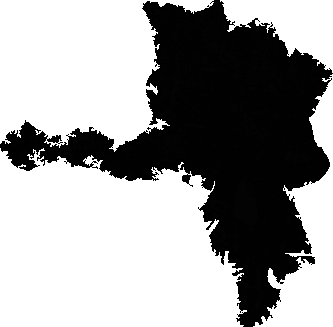

Supplement: Supplementary file 1 [file mmc1.zip › Supplementary/Landcover/Cochabamba_1990.tif]

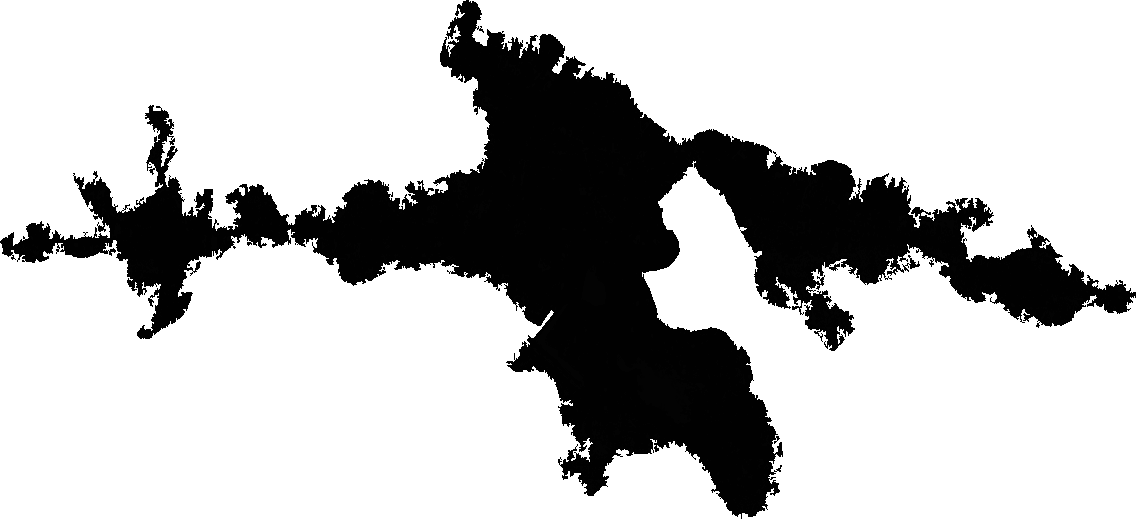

Supplement: Supplementary file 1 [file mmc1.zip › Supplementary/Landcover/Cochabamba_2015.tif]

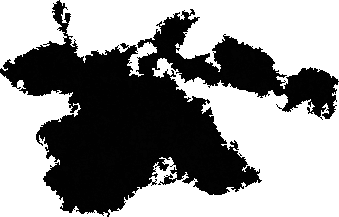

Supplement: Supplementary file 1 [file mmc1.zip › Supplementary/Landcover/Coimbatore_1990.tif]

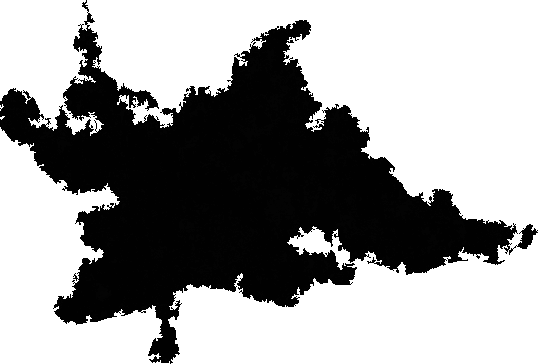

Supplement: Supplementary file 1 [file mmc1.zip › Supplementary/Landcover/Coimbatore_2015.tif]

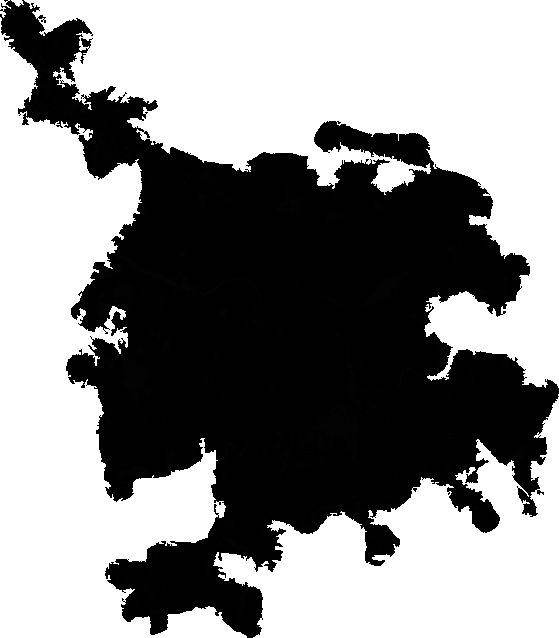

Supplement: Supplementary file 1 [file mmc1.zip › Supplementary/Landcover/Cordoba_1990.tif]

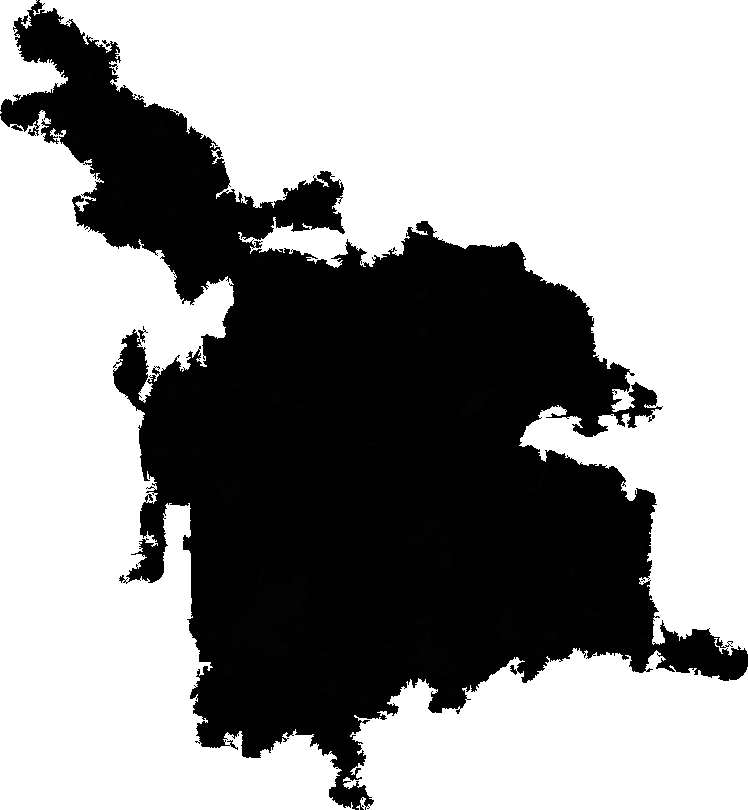

Supplement: Supplementary file 1 [file mmc1.zip › Supplementary/Landcover/Cordoba_2015.tif]

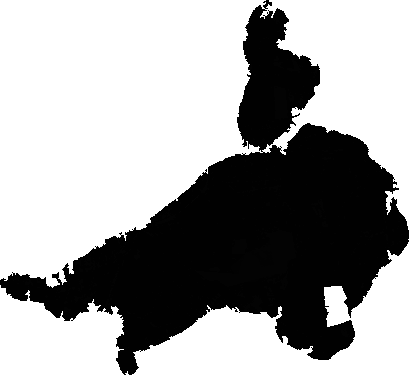

Supplement: Supplementary file 1 [file mmc1.zip › Supplementary/Landcover/Culiacan_1990.tif]

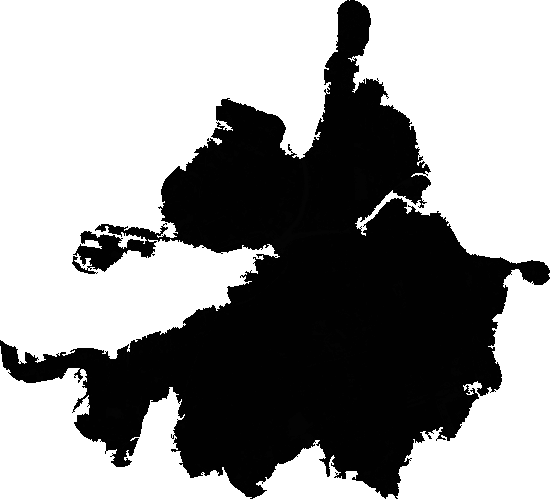

Supplement: Supplementary file 1 [file mmc1.zip › Supplementary/Landcover/Culiacan_2015.tif]

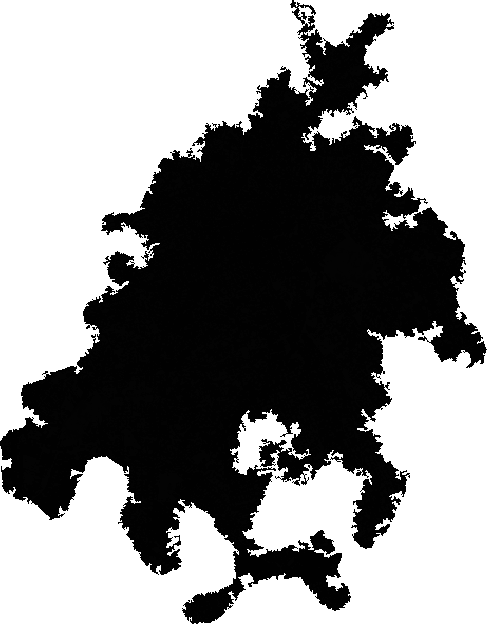

Supplement: Supplementary file 1 [file mmc1.zip › Supplementary/Landcover/Curitiba_1990.tif]

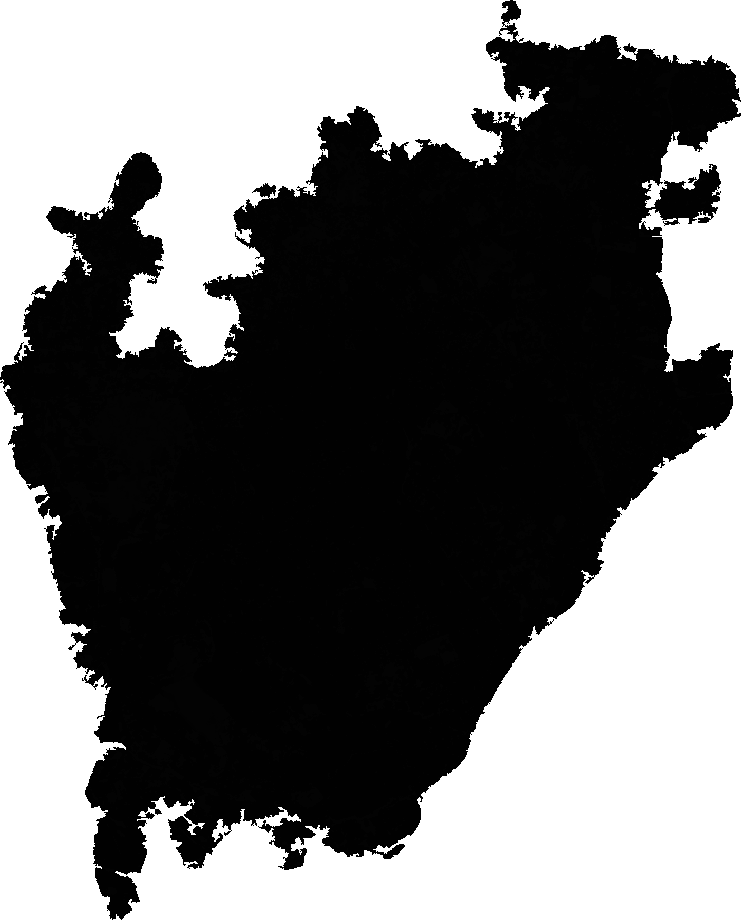

Supplement: Supplementary file 1 [file mmc1.zip › Supplementary/Landcover/Curitiba_2015.tif]

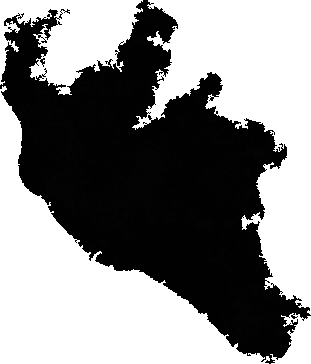

Supplement: Supplementary file 1 [file mmc1.zip › Supplementary/Landcover/Dhaka_1990.tif]

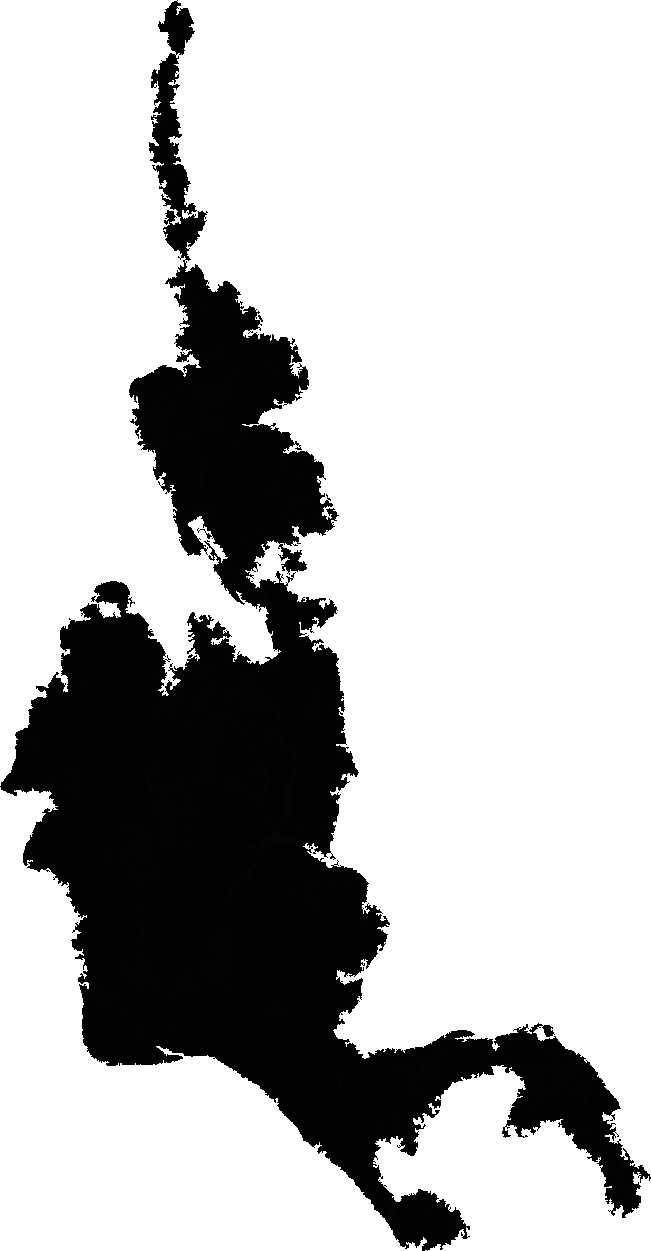

Supplement: Supplementary file 1 [file mmc1.zip › Supplementary/Landcover/Dhaka_2015.tif]

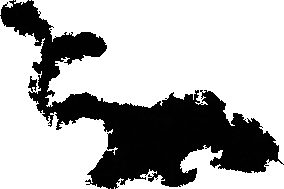

Supplement: Supplementary file 1 [file mmc1.zip › Supplementary/Landcover/Dzerzhinsk_1990.tif]

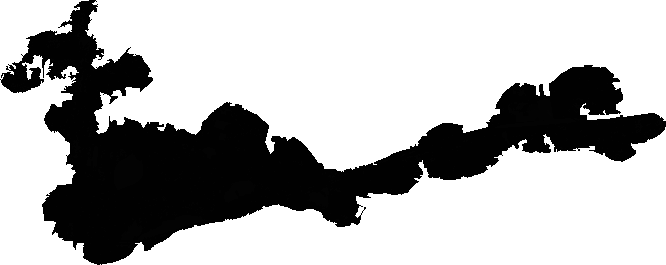

Supplement: Supplementary file 1 [file mmc1.zip › Supplementary/Landcover/Dzerzhinsk_2015.tif]

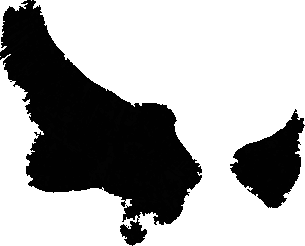

Supplement: Supplementary file 1 [file mmc1.zip › Supplementary/Landcover/Florianopolis_1990.tif]

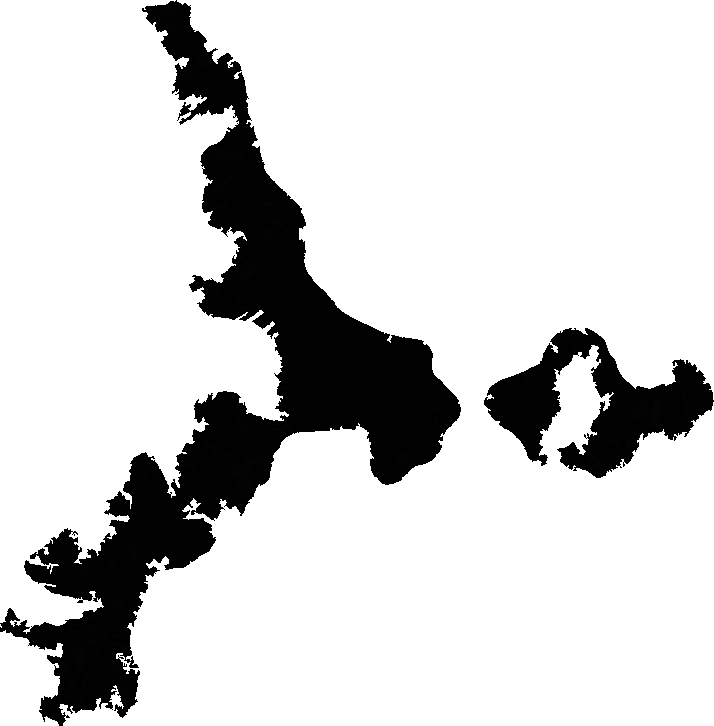

Supplement: Supplementary file 1 [file mmc1.zip › Supplementary/Landcover/Florianopolis_2015.tif]

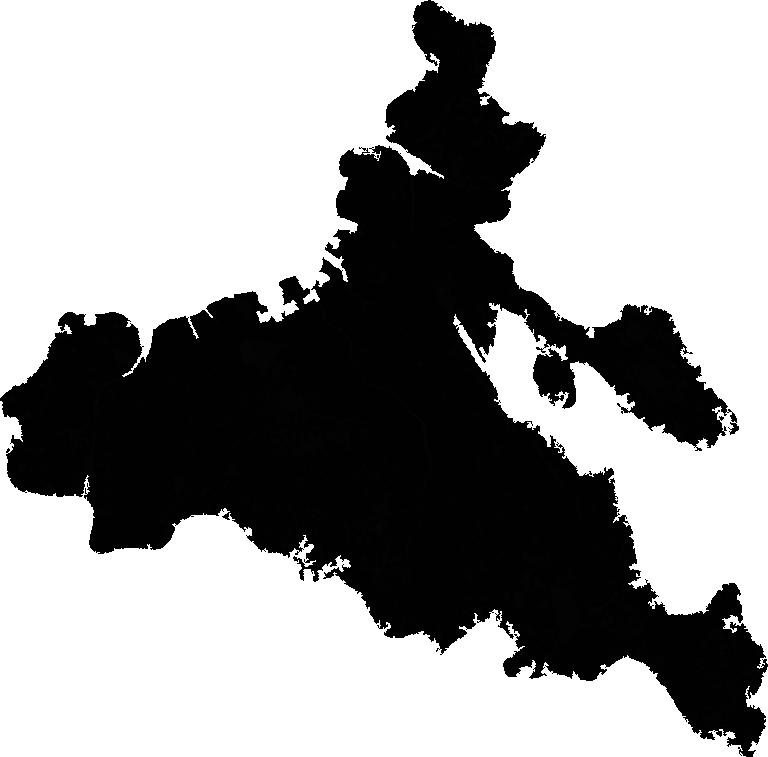

Supplement: Supplementary file 1 [file mmc1.zip › Supplementary/Landcover/Fukuoka_1990.tif]

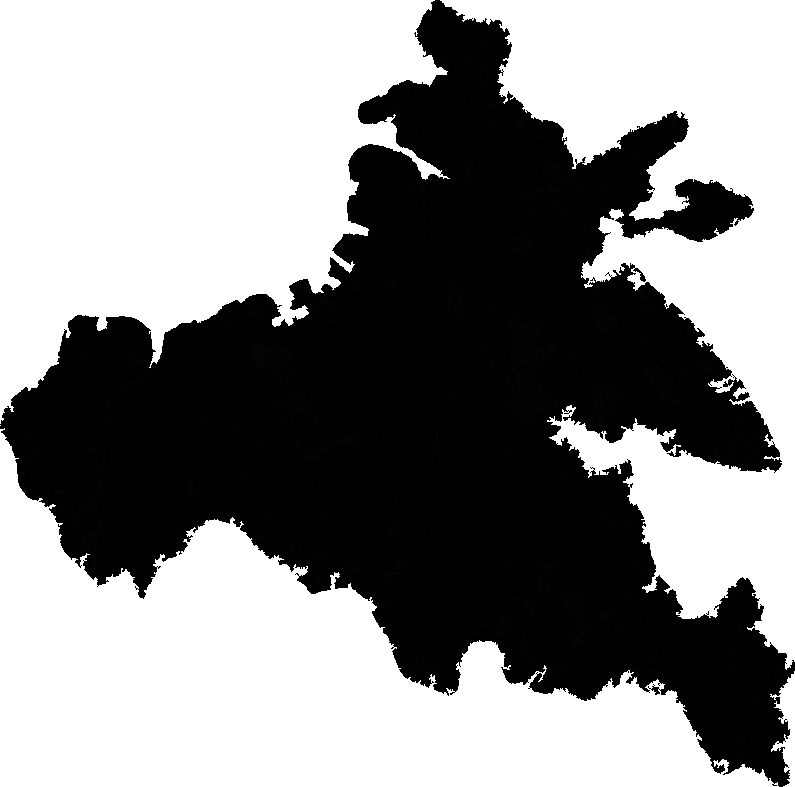

Supplement: Supplementary file 1 [file mmc1.zip › Supplementary/Landcover/Fukuoka_2015.tif]
